# Exosomal miR-196a derived from cancer-associated fibroblasts confers cisplatin resistance in head and neck cancer through targeting CDKN1B and ING5

**DOI:** 10.1186/s13059-018-1604-0

**Published:** 2019-01-14

**Authors:** Xing Qin, Haiyan Guo, Xiaoning Wang, Xueqin Zhu, Ming Yan, Xu Wang, Qin Xu, Jianbo Shi, Eryi Lu, Wantao Chen, Jianjun Zhang

**Affiliations:** 1 Department of Oral and Maxillofacial-Head & Neck Oncology, Ninth People’s Hospital, Shanghai Jiao Tong University School of Medicine, 639 Zhizaoju Road, Shanghai, 200011 People’s Republic of China; 2 Department of Clinical Laboratory, Ninth People’s Hospital, Shanghai Jiao Tong University School of Medicine, Shanghai, 201999 People’s Republic of China; 3 Shanghai Key Laboratory of Stomatology and Shanghai Research Institute of Stomatology, National Clinical Research Center of Stomatology, Shanghai, 200011 People’s Republic of China; 4 Department of Stomatology, Renji Hospital, Shanghai Jiao Tong University School of Medicine, 160 Pujian Road, Shanghai, 200127 People’s Republic of China

**Keywords:** Exosome, Cancer-associated fibroblasts, miR-196a, Cisplatin resistance, Head and neck cancer, CDKN1B, ING5

## Abstract

**Background:**

Cisplatin resistance is a major challenge for advanced head and neck cancer (HNC). Understanding the underlying mechanisms and developing effective strategies against cisplatin resistance are highly desired in the clinic. However, how tumor stroma modulates HNC growth and chemoresistance is unclear.

**Results:**

We show that cancer-associated fibroblasts (CAFs) are intrinsically resistant to cisplatin and have an active role in regulating HNC cell survival and proliferation by delivering functional miR-196a from CAFs to tumor cells via exosomes. Exosomal miR-196a then binds novel targets, CDKN1B and ING5, to endow HNC cells with cisplatin resistance. Exosome or exosomal miR-196a depletion from CAFs functionally restored HNC cisplatin sensitivity. Importantly, we found that miR-196a packaging into CAF-derived exosomes might be mediated by heterogeneous nuclear ribonucleoprotein A1 (hnRNPA1). Moreover, we also found that high levels of plasma exosomal miR-196a are clinically correlated with poor overall survival and chemoresistance.

**Conclusions:**

The present study finds that CAF-derived exosomal miR-196a confers cisplatin resistance in HNC by targeting CDKN1B and ING5, indicating miR-196a may serve as a promising predictor of and potential therapeutic target for cisplatin resistance in HNC.

**Electronic supplementary material:**

The online version of this article (10.1186/s13059-018-1604-0) contains supplementary material, which is available to authorized users.

## Background

Head and neck cancer (HNC), a common and aggressive malignant neoplasm associated with major morbidity and mortality, is still a leading global health burden [1]. Squamous cell carcinoma accounts for most HNC cases diagnosed worldwide, with an overall 5-year survival rate of approximately 50% [2]. Malignant proliferation and chemoresistance continue to be the limiting factors in HNC treatment, which leads to relapse and a poor outcome [2–4]. Despite therapeutic advancements, the overall prognosis has remained poor for the last 40 years [2]. Thus, a better understanding of the biological mechanisms of HNC is necessary for improving these dismal outcomes.

Current therapy options for HNC include surgery, radiotherapy, chemotherapy, and most recently anti-EGFR-antibody treatment [5]. For chemotherapy, cisplatin still acts as a core drug for patients with advanced HNC. However, patients always have a good initial response to cisplatin-based chemotherapy but later relapse because cisplatin resistance, either acquired or intrinsic, develops, markedly reducing clinical effectiveness [3]. Therefore, elucidating the underlying mechanisms of cisplatin resistance and discovering reliable biomarkers that predict the cisplatin response in HNC patients are urgent needs. Further studies have suggested that chemoresistance is due in part to the cell-autonomous functions of therapeutically resistant tumor cells and tumor stem cells [6]. However, the importance of the tumor microenvironment in dictating the treatment response is increasingly evident [7].

In the past decade, the role of the tumor microenvironment in tumor progression and chemoresistance has gathered great attention [8]. In our previous studies, cancer-associated fibroblasts (CAFs), a major component of the tumor stroma, were proven to play a critical role in facilitating HNC progression [9, 10]. An increasing amount of evidence has indicated that CAFs promote HNC progression by secreting growth factors, remodeling the extracellular matrix, and potentiating therapy resistance [8, 11]. Interestingly, recent studies have shown that exosomes, released from CAFs, have been found to increase tumor cell chemoresistance in many cancer types [12–16].

Exosomes (30–150 nm) are microvesicles that originate from multivesicular bodies (MVBs) and can participate in intercellular communication by transmitting intracellular cargos, including proteins, messenger RNAs (mRNAs), microRNAs (miRNAs), and long non-coding RNAs (lncRNAs) [17, 18]. Exosome-mediated miRNA delivery is widely believed to contribute to drug resistance development in many cancers [19]. In addition to tumor cell-secreted exosomal miRNAs, CAF-derived exosome-mediated miRNA delivery also plays an important role in chemoresistance. Richards et al. found that CAF-derived exosomal miR-146a promotes the survival and proliferation of pancreatic cancer cells during gemcitabine treatment [15]. In ovarian cancer, miR-21 is reportedly transferred from CAFs to cancer cells by exosomes, where it suppresses cancer cell apoptosis and confers paclitaxel resistance through targeting APAF1 [14]. Another study revealed that CAF-secreted exosomal miR-21 was proven to accelerate oxaliplatin resistance in colorectal cancer [20]. However, how CAFs react to cisplatin treatment and how CAF-derived exosomes contribute to cisplatin resistance in HNC are still unclear. Furthermore, the functional roles of these exosomal miRNAs in modulating cisplatin resistance in recipient HNC cells have not been elucidated.

Our previous studies suggested that CAFs might play a critical role in regulating cisplatin resistance in HNC cells [9, 10]. In this study, we aimed to investigate the effects of CAF-derived exosomes on the proliferation and survival of recipient HNC cells. Further experiments, including miRNA arrays, were performed to identify differential miRNA signatures in exosomes isolated from normal fibroblasts (NFs) and CAFs. Moreover, we demonstrated that miR-196a was directly transferred, through exosomes, from CAFs to HNC cells, and we also identified the molecular mechanisms by which exosomal miR-196a modulate cisplatin resistance in HNC cells.

## Results

### HNC-derived CAFs are innately chemoresistant

**Fig. 1:**
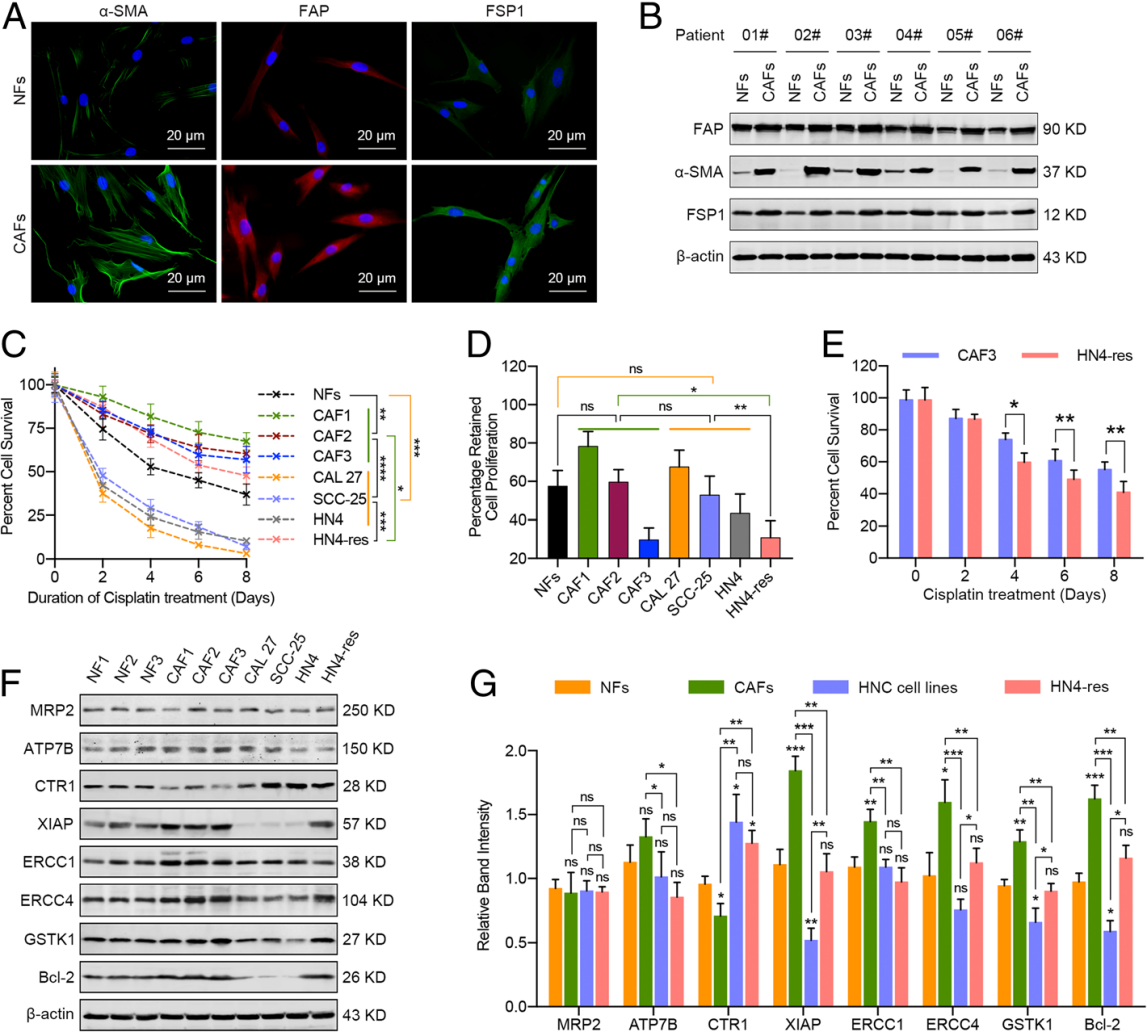
CAFs derived from HNC are innately resistant to cisplatin. **a** Immunofluorescence staining for α-SMA, FAP, and FSP1 expression of NFs and CAFs (scale bar, 20 μm). **b** Western blot analysis of α-SMA, FAP, and FSP1 protein levels in six paired NFs and CAFs. **c** NFs, CAFs, and HNC cells were treated with or without 10 μM cisplatin for 8 days, and cell viability was measured to obtain percent cell survival and was normalized to that of control cells. **d** NFs, CAFs, and HNC cells were treated with or without 10 μM cisplatin for 24 h, and cell viability was detected to calculate the percentage of proliferating cells during cisplatin treatment. **e** The percentage of surviving CAFs (CAF3) and cisplatin-resistant HN4-res cells, which exhibit similar proliferation retention rates, upon 10 μM cisplatin treatment for 8 days. **f**, **g** Western blot analysis of MRP2, ATP7B, CTR1, XIAP, ERCC1, ERCC4, GSTK1, and Bcl-2 protein levels in NFs, CAFs, CAL 27, SCC-25, HN4, and HN4-res cells. The band intensity was assessed. (ns, no significant difference; *p < 0.05; **p < 0.01; ***p < 0.001; ****p < 0.0001)

First, the innate cisplatin resistance of CAFs obtained from HNC tissues was compared with that of HNC cells. CAFs have a spindle-shaped morphology, are adherent in culture, and, compared with NFs, express higher levels of the specific fibroblast markers α-SMA, FAP, and FSP1 (Additional file 1: Figure S1a; Fig. 1a, b) [21]. As shown in Fig. 1c, survival rates were greater in CAFs than in cisplatin-resistant HN4-res and cisplatin-sensitive HNC cells (CAL 27, SCC-25, and HN4) treated with the same cisplatin dosage. Having identified that CAFs are resistant to cisplatin, we next assessed if the increased survival of CAFs exposed to cisplatin could be a result of CAFs undergoing senescence and not incorporating drug. The proliferation of cisplatin-treated CAFs and HNC cells was then analyzed. As a result, the most chemoresistant CAF cell line, CAF1, retained the most proliferation during cisplatin treatment, whereas the third leading cisplatin-resistant CAF cell line, CAF3, exhibited drastically decreased proliferation (Fig. 1d). In addition to proliferation, we attempted to investigate if other mechanisms also contributed to the cisplatin resistance of CAFs. We compared the survival rates of CAF3 and HN4-res cells with similar proliferation rates. Notably, CAF3 cells showed higher cell survival rates than HN4-res cells following cisplatin treatment (Fig. 1e). Additionally, the cisplatin IC50 was much higher in CAFs than in NFs, primary cancer cells, and HNC cell lines (Additional file 1: Figure S1b, c). The mechanisms which contributed to cisplatin resistance have been reported to include reduced cisplatin uptake (e.g., CTR1), increased drug efflux (e.g., MRP2, ATP7A, and ATP7B), increased detoxification (e.g., GSH and GST), alterations of DNA repair (e.g., ERCC1, ERCC4, MLH1, MSH2, and BRCA1/2), and alterations of antiapoptotic proteins (e.g., Bcl-2 and XIAP) [22]. Therefore, CTR1, MRP2, ATP7B, GST, ERCC1, ERCC4, Bcl-2, and XIAP, key regulators of intracellular drug resistance [6, 22], were selected for further analysis. As shown in Fig. 1f and g, the protein expression of CTR1, a plasma membrane copper transporter whose downregulation is associated with resistance to cisplatin, was lower in CAFs than in NFs or HNC cells. In addition, the protein levels of XIAP, ERCC1, ERCC4, GSTK1, and Bcl-2 were higher in CAFs than in NFs or HNC cells. Similar results were obtained when the mRNA expression levels of these genes were detected in CAFs, NFs, and HNC cells (Additional file 1: Figure S1d). ERCC1-ERCC4, a nucleotide excision repair-associated factor, was reported to be negatively correlated with survival and/or responsiveness to cisplatin-based regimens in HNC [22, 23]. Further analysis showed that silencing ERCC1 or ERCC4 by specific siRNAs markedly decreased the cisplatin resistance of CAFs, while knockdown of both ERCC1 and ERCC4 exhibited a more obvious effect (Additional file 1: Figure S1e–g). Taken together, these data revealed that CAFs are innately resistant to cisplatin.

### CAF-derived exosomes increase HNC cell proliferation and survival

**Fig. 2:**
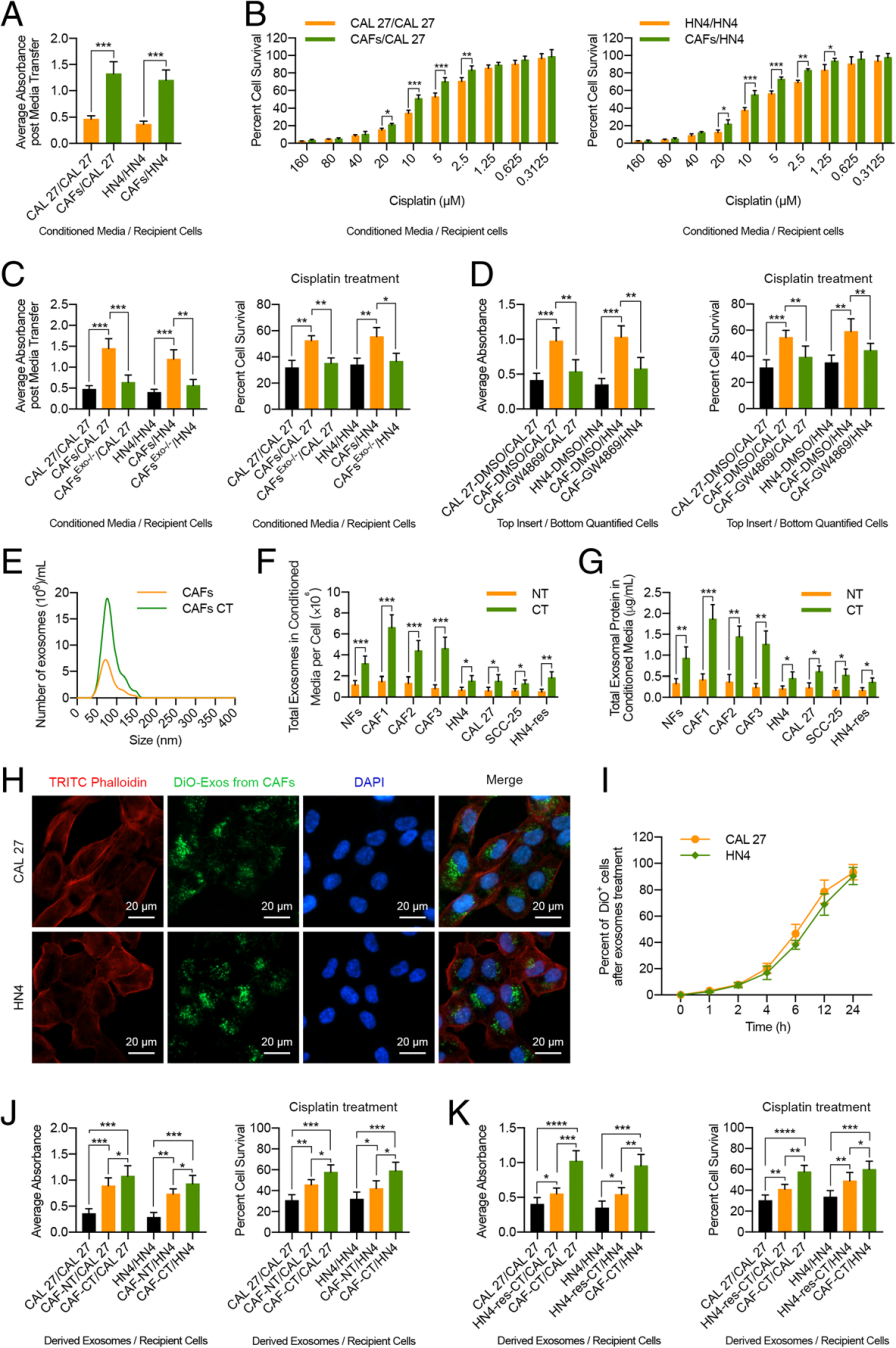
CAF-derived exosomes increase HNC cell proliferation and cisplatin resistance. **a** CAL 27 and HN4 cells were grown in CAF-CM or control CM for 6 days, and cell viability was examined. **b** CAL 27 and HN4 cells were grown in CAF-CM or control CM for 6 days, and then MTT assays were performed to detect the cisplatin response of these cells. **c** CAL 27 and HN4 cells were grown in control CM, CAF-CM, or exosome-depleted CAF-CM for 6 days, and cell viability was examined. MTT assays were performed to assess the tolerance of these cells to cisplatin. **d** HNC cells were co-cultured with DMSO-treated HNC cells, DMSO-treated CAFs, or GW4869-treated CAFs for 6 days, and then HNC cell viability was assessed. The survival percentage of co-cultured HNC cells was measured by MTT assay upon cisplatin treatment. **e** NanoSight particle-tracking analysis of size distribution and the number of exosomes from CAFs or cisplatin-treated CAFs (10 μM). **f** NanoSight particle-tracking analysis of the number of exosomes from NFs, CAFs, or HNC cells with or without cisplatin treatment (10 μM). **g** Exosomal protein concentration in CM from NFs, CAFs, or HNC cells with or without cisplatin treatment (10 μM). **h** CAL 27 and HN4 cells were incubated with DiO-labeled exosomes (25 μg/mL) from CAFs for 24 h, and the green exosome signal was detected by confocal microscopy (scale bar, 20 μm). **i** Flow cytometric analysis of DiO-positive CAL 27 or HN4 cells after incubating with DiO-labeled exosomes (25 μg/mL) from CAFs for the indicated time. **j** HNC cells were treated with exosomes (25 μg/mL) from HNC cells, CAFs, or cisplatin-treated (10 μM) CAFs for 6 days. Cell viability was measured, and MTT assays were performed to evaluate the cisplatin response of these cells. **k** HNC cells were treated with exosomes (25 μg/mL) from HNC cells, cisplatin-treated (10 μM) HN4-res cells, or cisplatin-treated (10 μM) CAFs for 6 days. The viability and survival percentage of these cells were measured by MTT assays. (NT, without cisplatin treatment; CT, cisplatin treatment; ns, no significant difference; *p < 0.05; **p < 0.01; ***p < 0.001; ****p < 0.0001)

Considering the innate chemoresistance of CAFs, we next evaluated whether functional factors secreted by CAFs affected HNC cell proliferation and survival. First, we determined the effects of CAF-conditioned medium (CM) on cisplatin-sensitive HNC cell proliferation and survival. The results revealed that compared with NF-CM and tumor cell-CM, CAF-CM significantly promoted CAL 27 and HN4 cell proliferation (Fig. 2a; Additional file 1: Figure S2a). However, CM from HN4-res cells did not elicit a significant increase in CAL 27 and HN4 cell proliferation (Additional file 1: Figure S2b), which indicated that this effect was CAF-specific. Simultaneously, we observed that cell survival was significantly increased in HNC cells grown in CAF-CM and subsequently treated with cisplatin compared with HNC cells grown in tumor cell-CM, NF-CM, or HN4-res-CM (Fig. 2b; Additional file 1: Figure S2a, b). These results revealed that CAF-CM, which contained almost all secreted factors, could affect HNC cell proliferation and cisplatin resistance.

Exosomes, nanometric membrane vesicles secreted by almost all kinds of cell types, play an important role in intercellular communication. Since CAF-CM regulates HNC cell growth and cisplatin resistance, we tried to investigate whether CAF-derived exosomes might contribute to this effect. Fortunately, we found that physically removing exosomes from CAF-CM by ultracentrifugation or pharmacologically blocking the exosome secretion of CAFs by GW4869 (Additional file 1: Figure S3) markedly reduced the ability of CAF-CM to increase CAL 27 and HN4 cell proliferation and cisplatin resistance (Fig. 2c, d). We then purified exosomes from CAF-CM through ultracentrifugation and confirmed their identity by positive exosome markers (Alix, HSP90, HSP70, CD63, CD9, and Rab5) and the negative marker GRP94 [24, 25] (Additional file 1: Figure S4a). CAF-derived exosomes were approximately 100 nm in diameter and exhibited typical cup-shaped morphology, which was confirmed using particle size analysis and transmission electron microscopy (Additional file 1: Figure S4b, c). Intriguingly, we found that compared with NFs and HNC cells, CAFs displayed the largest increase in exosome release after cisplatin treatment (Fig. 2e–g). Further analysis showed that the mRNA expression levels of CD63, nSMase2, RAB35, RAB5A, RAB9A, RAB27A, and RAB7 were increased in CAFs after cisplatin treatment (Additional file 1: Figure S5), suggesting that cisplatin might promote the biogenesis of exosomes by upregulating the expression of CD63 and nSMase2 and accelerate the transport as well as the release of exosomes via increased expression of RAB35, RAB5A, RAB9A, RAB27A, and RAB7 in CAFs [26].

To determine if CAF-derived exosomes are internalized by HNC cells, exosomes were purified from CAF-CM, labeled with 3,3′-dioctadecyloxacarbocyanine perchlorate (DiO), and incubated with HNC cells for 24 h. We observed that DiO-labeled exosomes were internalized by HNC cells (Fig. 2h, i). Functionally, compared with exosomes of NFs, CAF- or cisplatin-treated CAF-derived exosomes significantly promoted the proliferation and chemoresistance in HNC cells (Fig. 2j; Additional file 1: Figure S2c), while exosomes from chemoresistant HN4-res cells elicited a milder effect (Fig. 2k; Additional file 1: Figure S2d). Collectively, these data indicated that exosomes released by CAFs or cisplatin-treated CAFs accelerate the growth and survival of recipient HNC cells.

### Exosomal transfer of miR-196a from CAFs to HNC cells

**Fig. 3:**
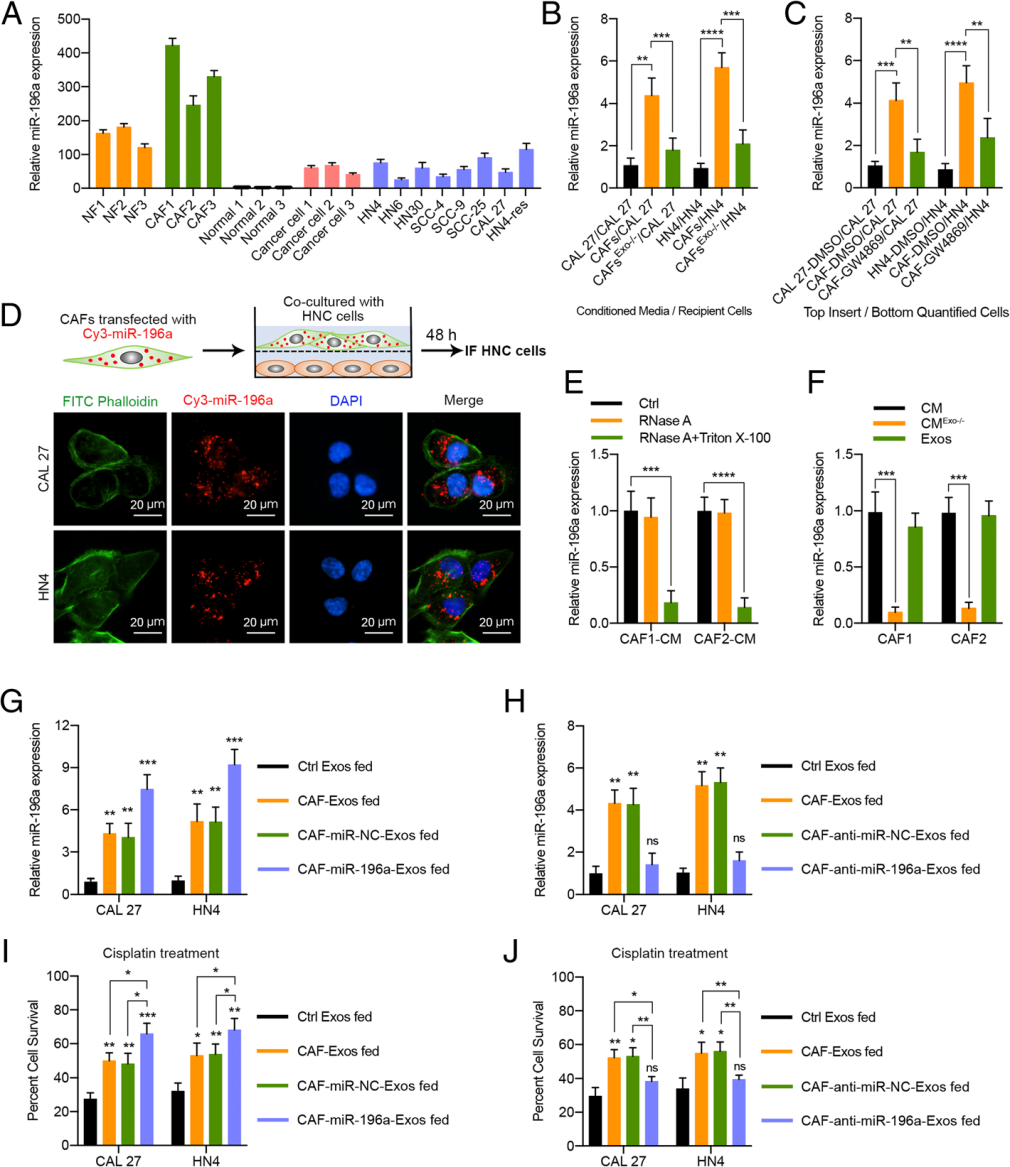
Exosomal transfer of miR-196a from CAFs to HNC cells. **a** miR-196a expression in NFs, CAFs, normal oral epithelial cells (titled normal), primary cancer cells, and HNC cell lines was analyzed using real-time PCR. **b** CAL 27 and HN4 cells were incubated with control CM, CAF-CM, or exosome-depleted CAF-CM for 24 h. The miR-196a expression level in these cells was determined using real-time PCR. **c** CAL 27 and HN4 cells were co-cultured with DMSO-treated HNC cells, DMSO-treated CAFs, or GW4869-treated CAFs for 24 h. The miR-196a expression level was then detected in HNC cells using real-time PCR. **d** CAFs transiently transfected with Cy3-tagged miR-196a (Cy3-miR-196a) were co-cultured with CAL 27 or HN4 cells for 48 h. Fluorescence microscopy was used to detect the red fluorescent signals in HNC cells (scale bar, 20 μm). **e** Real-time PCR analysis of miR-196a expression in CAF-CM treated with RNase A (2 mg/mL) alone or combined with Triton X-100 (0.1%) for 20 min. **f** Real-time PCR analysis of miR-196a expression in exosomes, exosome-depleted CM, and whole CM derived from CAFs. **g** miR-196a expression in CAL 27 and HN4 cells was detected by real-time PCR at 24 h after incubation with exosomes (25 μg/mL) from HNC cells (Ctrl Exos), CAFs, or CAFs transfected with or without miR-196a mimics. **h** miR-196a expression in CAL 27 and HN4 cells was detected by real-time PCR at 24 h after incubation with exosomes (25 μg/mL) from HNC cells (Ctrl Exos), CAFs, and CAFs transfected with or without anti-miR-196a. **i**, **j** CAL 27 and HN4 cells were treated with the indicated exosomes (25 μg/mL) for 6 days, and the cisplatin response in these cells was determined with MTT assays. (ns, no significant difference; *p < 0.05; **p < 0.01; ***p < 0.001; ****p < 0.0001)

To investigate the functional molecules responsible for the ability of CAF-derived exosomes to promote proliferation and cisplatin resistance in recipient HNC cells, the miRNA expression levels of NF- and CAF-derived exosomes were analyzed via miRNA array. We observed that CAF-derived exosomes, especially cisplatin-treated CAF-derived exosomes, showed a marked increase in miR-196a-5p (also known as miR-196a) expression (Additional file 2: Table S1; Additional file 1: Figure S6a, b). Mechanistic analysis showed that the p38 and ERK MAPK signaling pathways were involved in the regulation of miR-196a expression in cisplatin-stimulated CAFs by stimulating c-Myc binding to the miR-196a promoter (Additional file 1: Figure S7a–h). In addition, a significant increase in miR-196a expression was detected in HNC cells after incubation with exosomes from cisplatin-treated CAFs (Additional file 1: Figure S6c, d). Furthermore, the miR-196a level was upregulated in HNC tissues compared with adjacent normal tissues (Additional file 1: Figure S6e, f), and compared with HNC cells and NFs, CAFs expressed significantly higher levels of endogenous miR-196a (Fig. 3a; Additional file 1: Figure S6g). These results suggest that CAFs in the tumor microenvironment might increase miR-196a expression levels in HNC cells through direct miR-196a transfer.

To determine whether miR-196a is directly transferred from CAFs to tumor cells via exosomes, HNC cells were incubated with CAF-CM and exosome-depleted CAF-CM, respectively. We observed that HNC cells grown in CAF-CM expressed a higher level of miR-196a; however, miR-196a expression in HNC cells was substantially reduced when exosomes from CAF-CM were depleted physically or pharmacologically (Fig. 3b, c). Moreover, CAFs transiently transfected with Cy3-tagged miR-196a were co-cultured with HNC cells for 48 h. The fluorescently labeled miR-196a was observed in the cancer cells through confocal microscopy (Fig. 3d), suggesting that miR-196a was transferred from CAFs to HNC cells via exosomes. In addition, the levels of miR-196a in CAF-CM were unchanged upon RNase A treatment but significantly decreased when treated with RNase A plus Triton X-100 simultaneously (Fig. 3e), indicating that extracellular miR-196a was mainly encased within the membrane instead of directly released. Interestingly, miR-196a levels were almost equal in exosomes and whole CAF-CM (Fig. 3f). In addition, intracellular miR-196a levels were significantly increased upon incubation with exosomes from CAFs with miR-196a overexpression but not with CAFs with miR-196a knockdown (Additional file 1: Figure S8a, b; Fig. 3g, h). Functionally, HNC cells became insensitive to cisplatin when incubated with exosomes from CAFs, especially CAFs with miR-196a overexpression, whereas miR-196a knockdown in CAFs abolished this effect (Fig. 3i, j). These findings revealed that functional miR-196a could be transferred from CAFs to HNC cells via exosomes.

### miR-196a packaging into exosomes is mediated by hnRNPA1

**Fig. 4:**
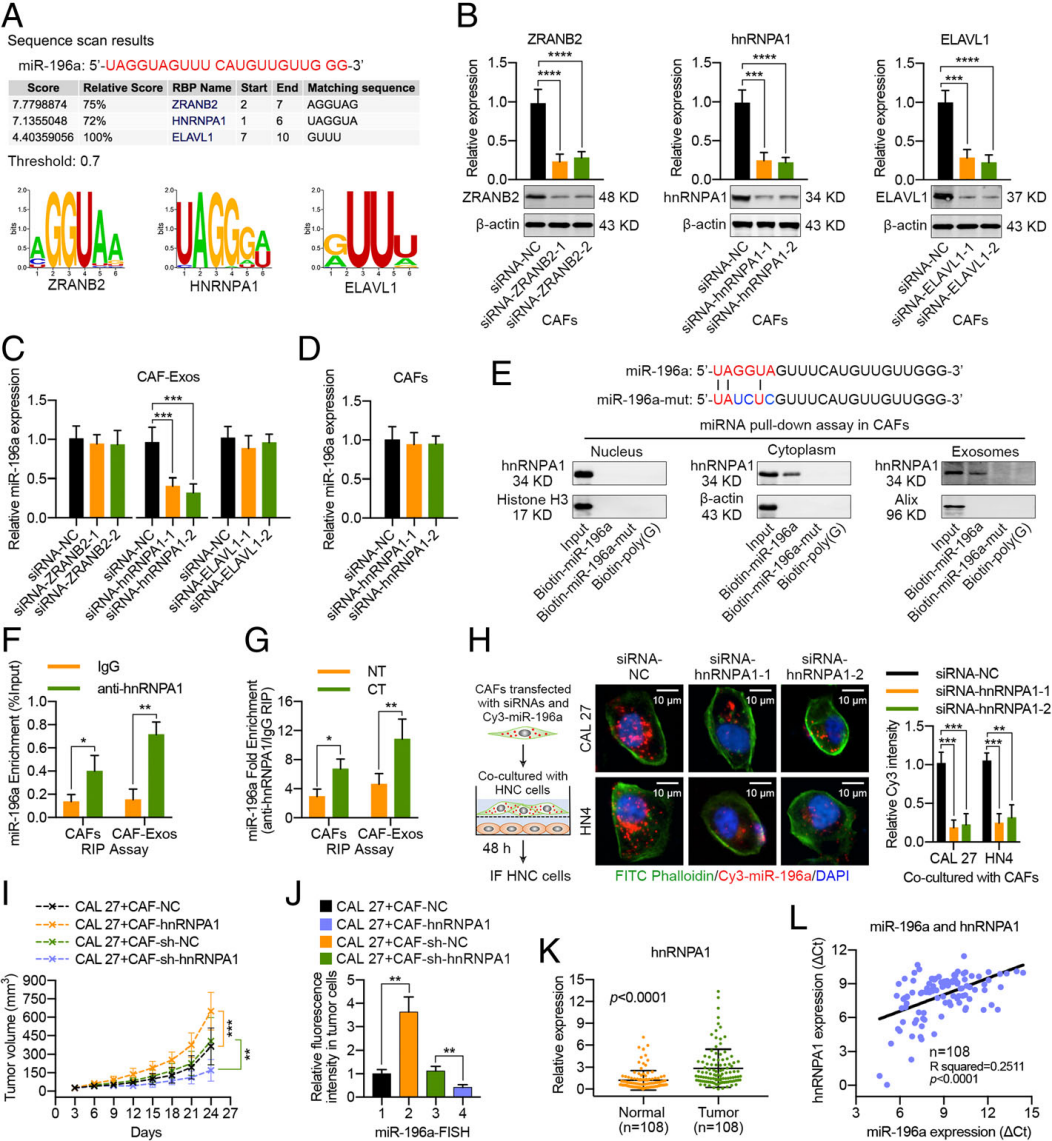
The hnRNPA1 protein mediates miR-196a packaging into CAF-derived exosomes. **a** A specific interaction between the miR-196a sequence and RBP motifs was predicted through RBPDB analysis (threshold 0.7). **b** Western blot and real-time PCR results showing ZRANB2, hnRNPA1, and ELAVL1 expression levels in CAFs at 48 h after transfection with specific siRNAs. **c** miR-196a expression in exosomes from CAFs transfected with specific siRNAs targeting ZRANB2, hnRNPA1, or ELAVL1 was measured using real-time PCR. **d** Real-time PCR analysis showing miR-196a expression in CAFs with hnRNPA1 silenced. **e** Western blot analysis of hnRNPA1 expression in samples derived by miRNA pulldowns performed with nuclear, cytoplasmic, or exosomal CAFs lysates and the indicated biotinylated miR-196a or mutated miR-196a; biotinylated poly(G) was used as a negative control. **f** RIP assays with anti-hnRNPA1 antibody (or IgG as control) were performed on the cell or exosomal lysates from CAFs. miR-196a levels in immunoprecipitated samples were determined by real-time PCR and were reported as percentages in respect to the input sample (% input). **g** RIP assay to determine hnRNPA1 enrichment on miR-196a relative to IgG in cytoplasmic or exosomal lysates of CAFs treated with or without cisplatin. **h** CAL 27 and HN4 cells were co-cultured with CAFs concurrently transfected with Cy3-miR-196a and specific siRNAs targeting hnRNPA1 for 48 h. Fluorescence microscopy was used to detect red fluorescent signals in HNC cells (scale bar, 10 μm). **i** Nude mice were subcutaneously xenografted with a mixture of CAL 27 cells plus CAFs transfected with hnRNPA1, sh-NC, or sh-hnRNPA1, and the tumor growth curve and tumor volumes are shown. **j** The distribution of miR-196a in xenograft tumors was detected using FISH assay, and miR-196a expression in tumor cells was assessed. **k** The hnRNPA1 mRNA expression level in 108 pairs of HNC samples and adjacent normal tissues. **l** Correlation analysis was performed between miR-196a expression and hnRNPA1 expression in HNC tissues (*n* = 108) (NT, without cisplatin treatment; CT, cisplatin treatment; *p < 0.05; **p < 0.01; ***p < 0.001; ****p < 0.0001)

To investigate whether miR-196a was specifically packaged into exosomes, we analyzed the specific interaction between the miR-196a sequence and motifs of RNA-binding proteins (RBPs) by using the database of RBP specificities (RBPDB, http://rbpdb.ccbr.utoronto.ca/; threshold 0.7) [27], and the results revealed that the zinc finger ran-binding domain-containing protein 2 (ZRANB2), heterogeneous nuclear ribonucleoprotein A1 (hnRNPA1), and ELAV-like protein 1 (ELAVL1) motifs had specific miR-196a binding sites (Fig. 4a). Further investigations revealed that hnRNPA1 knockdown with specific siRNAs in CAFs significantly decreased exosomal miR-196a levels, while the cellular miR-196a level was nearly unchanged (Fig. 4b–d), indicating that hnRNPA1 played a regulatory role in exosomal miR-196a levels. Moreover, miRNA pull-down assays showed that an interaction between hnRNPA1 and miR-196a was observed in the cytoplasm and exosomes but not in the nucleus. However, hnRNPA1-binding capacity was impaired when the UAGGUA sequence of miR-196a was mutated (Fig. 4e). In addition, RNA immunoprecipitation (RIP) assays within the cell and exosome lysates of CAFs were then performed, and we found that miR-196a was enriched in the hnRNPA1 antibody group compared with the IgG group (Fig. 4f). Meanwhile, compared with control group lysates, the cell and exosome lysates from cisplatin-treated CAFs showed significant miR-196a enrichment (Fig. 4g). Functionally, confocal analysis revealed that miR-196a transfer from CAFs to HNC cells via exosomes was reduced when CAFs were transfected with hnRNPA1-specific siRNAs in advance (Fig. 4h). In addition, the mRNA and protein expression levels of hnRNPA1 in CAFs remained essentially unchanged after cisplatin treatment, whereas increased hnRNPA1 protein levels were observed in the exosomes from cisplatin-stimulated CAFs (Additional file 1: Figure S7i). Because cisplatin could promote the miR-196a expression in CAFs (Additional file 1: Figure S7a–h), we speculated that cisplatin accelerated the production of exosomal miR-196a by CAFs by upregulating the cellular miR-196a expression and hnRNPA1 translocation from cells to exosomes, thereby mediating greater packaging of miR-196a into exosomes.

We subsequently identified the effective role of CAF-derived hnRNPA1 in tumor growth in vivo. The results of the xenograft assay revealed that the tumors formed by CAL 27 plus CAF-hnRNPA1 cells grew faster than those in the control group, and silencing hnRNPA1 expression in CAFs significantly reduced tumor growth (Additional file 1: Figure S9a, b; Fig. 4i). Moreover, fluorescence in situ hybridization (FISH) assays revealed that miR-196a expression in tumor cells was greater when hnRNPA1 expression was upregulated in the nearby CAFs, whereas suppressing hnRNPA1 expression in CAFs resulted in a low miR-196a signal in tumor cells (Fig. 4j; Additional file 1: Figure S9c, d). Additionally, we also found that hnRNPA1 was upregulated in HNC tissues compared with adjacent normal tissues, and hnRNPA1 expression in HNC was positively correlated with miR-196a expression (Fig. 4k, l). Further analysis revealed that the upregulation of hnRNPA1 in HNC was closely related to increased tumor size, lymph node metastasis, advanced tumor stage, and chemoresistance (Additional file 1: Figure S10).

The above data demonstrated that hnRNPA1 might play an important role in packaging miR-196a into exosomes by binding a specific motif (UAGGUA) existing at the 5′ end of miR-196a, suggesting that miR-196a is specifically sorted into CAF-derived exosomes. Importantly, the hnRNPA1-mediated miR-196a sorting mechanism might play an active role in HNC progression and chemoresistance.

### miR-196a promotes HNC cell proliferation and cisplatin resistance

**Fig. 5:**
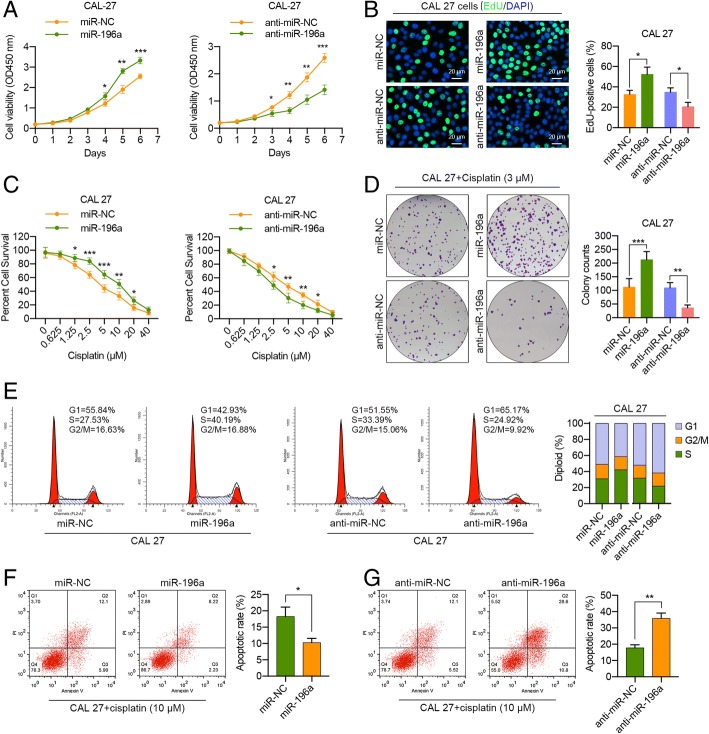
miR-196a regulates HNC cell proliferation and survival by promoting G1/S transition and apoptosis resistance. **a** Cell proliferation assays with CAL 27 cells at 48 h after transfection with miR-196a or anti-miR-196a. **b** Representative micrographs and quantification of EdU-incorporating cells at 48 h after transfection with miR-196a or anti-miR-196a. **c** MTT assay of CAL 27 cells transfected with miR-196a or anti-miR-196a for 48 h, followed by cisplatin treatment at the indicated concentration for 72 h. **d** Plate colony formation assay of CAL 27 cells transfected with miR-196a or anti-miR-196a with cisplatin treatment (3 μM). **e** The cell cycle distribution was analyzed by a flow cytometer in CAL 27 cells transfected with miR-196a or anti-miR-196a at 48 h after transfection. **f**, **g** Flow cytometric analysis of cisplatin-induced (10 μM) apoptosis in CAL 27 cells transfected with miR-196a or anti-miR-196a at 48 h after transfection. (*p < 0.05; **p < 0.01; ***p < 0.001)

Having determined that cancer cells could uptake CAF-derived exosomal miR-196a, we next sought to investigate whether miR-196a might contribute to HNC cell chemoresistance. As a result, miR-196a overexpression (Additional file 1: Figure S8a) markedly increased the growth rate of CAL 27 and HN4 cells (Fig. 5a; Additional file 1: Figure S11a), and a higher percentage of EdU-positive cells was observed in miR-196a-transfected CAL 27 and HN4 cells than control cells (Fig. 5b; Additional file 1: Figure S11b). Simultaneously, MTT and colony formation assays showed that miR-196a overexpression substantially promoted cisplatin resistance in CAL 27 and HN4 cells (Fig. 5c, d; Additional file 1: Figure S11c, d). In contrast, miR-196a knockdown in HNC cells (Additional file 1: Figure S8b) dramatically reduced tumor cell proliferation and chemoresistance (Fig. 5a–d; Additional file 1: Figure S11a–d). Since miR-196a significantly affected CAL 27 and HN4 cell proliferation and cisplatin resistance, we hypothesized that miR-196a could function by affecting the cell cycle or cell apoptosis of HNC cells. Flow cytometric analysis revealed that ectopic miR-196a expression drastically decreased the percentage of cells in the G1 peak and increased the percentage of cells in the S peak in both CAL 27 and HN4 cells compared with control cells. As expected, miR-196a knockdown dramatically increased the number of cells in the G1 peak and decreased those in S peak (Fig. 5e; Additional file 1: Figure S11e). Similarly, miR-196a overexpression inhibited the basal apoptotic rate and cisplatin-induced apoptosis in CAL 27 and HN4 cells, while miR-196a knockdown significantly increased basal apoptosis and cisplatin-induced apoptosis in HNC cells (Fig. 5f, g; Additional file 1: Figures S11f, g and S12). Hence, we concluded that miR-196a could enhance proliferation and cisplatin resistance in HNC cells by promoting G1/S cell cycle transition and inhibiting cell apoptosis.

Since other miRNAs (miR-10b-5p, miR-708-5p, miR-335-5p, miR-10a-5p, and miR-218-5p) were upregulated in the exosomes from cisplatin-treated CAFs (Additional file 1: Figures S6a–d), we attempted to determine whether also these five miRNAs were involved in cisplatin resistance or if there was a synergistic effect. We observed that the survival rates of HNC cells transfected with miR-10b-5p or miR-10a-5p were obviously increased, and miR-196a exhibited a more active role in cisplatin resistance. However, the factorial analysis showed that there was no synergistic effect of miR-196a and miR-10b-5p or miR-196a and miR-10a-5p (Additional file 1: Figures S13). These data emphasized the importance of miR-196a among these miRNAs in cisplatin resistance.

### CDKN1B and ING5 are direct targets of exosomal miR-196a in HNC

**Fig. 6:**
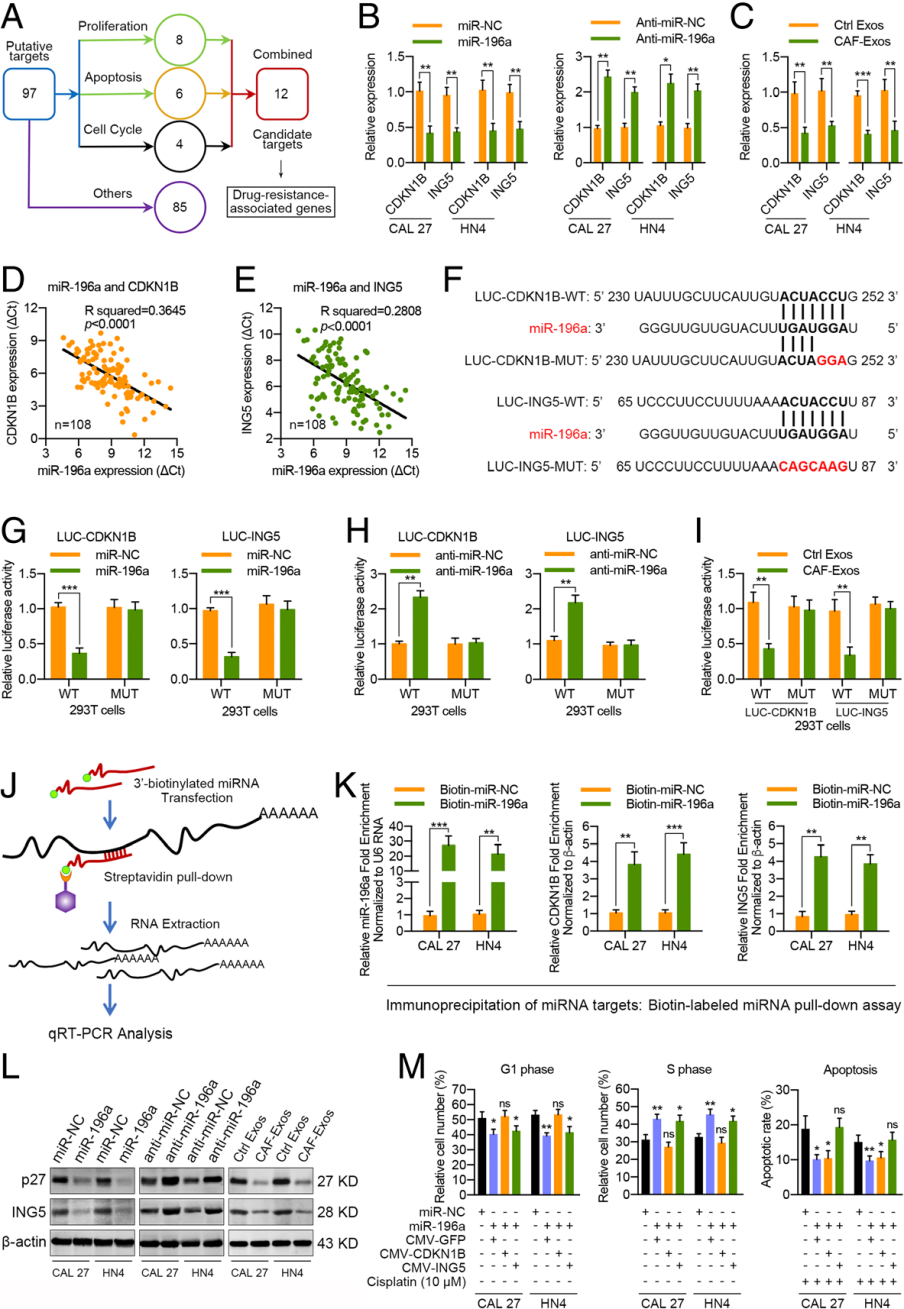
CDKN1B and ING5 are direct targets of exosomal miR-196a in HNC cells. **a** A diagram showing the predicted candidate target genes of miR-196a by Gene Ontology analysis. **b** CDKN1B and ING5 mRNA expression in CAL 27 and HN4 cells transfected with miR-196a or anti-miR-196a at 48 h after transfection. **c** CDKN1B and ING5 mRNA levels in CAL 27 and HN4 cells at 48 h after incubation with exosomes (25 μg/mL) from HNC cells (Ctrl Exos) and CAFs. **d**, **e** Correlation analysis was performed between miR-196a expression and CDKN1B or ING5 expression in HNC tissues (*n* = 108). **f** Predicted miR-196a target sequences in the 3′ UTRs of CDKN1B and ING5 genes. **g** Relative CDKN1B or ING5 reporter activities in 293T cells co-transfected with miR-196a and luciferase reporters. **h** The effects of anti-miR-196a on CDKN1B or ING5 reporter luciferase activity in 293T cells. **i** The effects of CAF-derived exosomes (25 μg/mL) on CDKN1B or ING5 reporter luciferase activity in 293T cells. **j** A diagram showing the program for the immunoprecipitation of miRNA targets. **k** Interaction of target transcripts with miR-196a. CAL 27 or HN4 cells were transfected with biotinylated miR-NC or miR-196a for 48 h. Levels of CDKN1B and ING5 mRNA in the materials pulled down by biotin-miR-196a were analyzed by real-time PCR and normalized to β-actin. **l** CAL 27 and HN4 cells were transfected with miR-196a or anti-miR-196a or were incubated with CAF-derived exosomes (25 μg/mL) for 48 h. p27 and ING5 expression in the indicated cells was detected by western blot analysis. **m** The results from the cell cycle and cisplatin-induced cell apoptosis analyses in HNC cells transfected with miR-NC, miR-196a plus control vector, miR-196a plus CDKN1B plasmid, or miR-196a plus ING5 plasmid at 48 h after transfection. (ns, no significant difference; *p < 0.05; **p < 0.01; ***p < 0.001)

To identify the target genes of miR-196a regulation, we used a commonly used algorithm, miRecords (http://c1.accurascience.com/miRecords/), which is an integrated resource for animal miRNA-target interactions. To increase the accuracy of this prediction, genes that were predicted by at least 5 of 11 databases in miRecords were selected as putative targets. Among these putative targets, gene ontology analysis (http://david.abcc.ncifcrf.gov/) revealed 12 candidate genes whose altered expression could contribute to proliferative or chemoresistant phenotypes (Fig. 6a; Additional file 3: Table S2). Thus, we selected these 12 genes as potential downstream miR-196a target genes. Real-time PCR analysis revealed that miR-196a overexpression significantly inhibited CDKN1B and ING5 expression in CAL 27 and HN4 cells, while anti-miR-196a exhibited the opposite effect (Fig. 6b; Additional file 1: Figure S14a, b). As expected, CAF-derived exosomal miR-196a markedly inhibited CDKN1B and ING5 expression in CAL 27 and HN4 cells (Fig. 6c; Additional file 1: Figure S15a). In HNC samples, we found an inverse correlation between CDKN1B and miR-196a expression, and miR-196a overexpression was also associated with ING5 downregulation (Additional file 1: Figure S14c–e; Fig. 6d, e). The above data demonstrated that CDKN1B and ING5 were putative direct miR-196a targets. Using 3′ UTR luciferase reporter assays, we found that miR-196a overexpression significantly inhibited luciferase activity in 293T or HNC cells expressing wild-type CDKN1B and ING5 3′ UTR reporters, whereas anti-miR-196a specifically abolished this suppression. Moreover, mutations in the miR-196a-binding seed region of the CDKN1B and ING5 3′ UTR abrogated the repressive effect of miR-196a (Fig. 6f–h; Additional file 1: Figure S16a, b). These putative binding sites for miR-196a in the 3′ UTR sequence of CDKN1B and ING5 mRNA are evolutionally conserved among vertebrates (Additional file 1: Figure S17). In addition, CAF-secreted exosomal miR-196a also effectively decreased luciferase activity in 293T or HNC cells expressing wild-type CDKN1B and ING5 3′ UTR reporters (Fig. 6i; Additional file 1: Figures S15b; S16c). To test for association of miR-196a with its respective target transcripts (CDKN1B and ING5), we adopted a recently described method for capturing miRNA-mRNA complexes using streptavidin-coated beads from cells transfected with biotinylated forms of the miRNA mimics [28]. Interestingly, the mRNA levels of CDKN1B and ING5 were markedly enriched in the pull-down material isolated from the HNC cells transfected with biotin-labeled miR-196a (Fig. 6j, k). As shown in Fig. 6l, ectopic miR-196a expression or treatment with CAF-derived exosomes decreased p27 (coded by CDKN1B) and ING5 protein expression in CAL 27 and HN4 cells, whereas miR-196a knockdown increased these expression levels. Further experiments revealed that CAF-derived exosomal miR-196a could functionally regulate p27 and ING5 protein expression in HNC cells (Additional file 1: Figure S15c). These data suggested that CDKN1B and ING5 are genuine targets of exosomal miR-196a.

### CDKN1B and ING5 exhibit different functions in miR-196a-mediated cisplatin resistance

After identifying CDKN1B and ING5 as direct targets of miR-196a, we analyzed the functional roles of CDKN1B and ING5 in chemoresistance. Silencing CDKN1B or ING5 by specific siRNAs markedly promoted HNC cell proliferation and cisplatin resistance (Additional file 1: Figure S18a–c), whereas CDKN1B or ING5 overexpression dramatically decreased the growth and cisplatin resistance of these cells (Additional file 1: Figure S18d–f). Interestingly, we observed that CDKN1B dysregulation was primarily associated with G1/S cell cycle transition, while the ING5 gene mainly regulated cisplatin-induced cell apoptosis (Additional file 1: Figure S18g–j). Moreover, the miR-196a-mediated G1/S cell cycle transition could be rescued by ectopic CDKN1B expression but not ING5 expression. Similarly, inhibited miR-196a-mediated apoptosis could be rescued by exogenous ING5 expression rather than CDKN1B expression (Fig. 6m). In addition, CAF-derived exosomal miR-196a could modulate the cell cycle and cisplatin-induced apoptosis in HNC cells by downregulating p27 or ING5 protein expression (Additional file 1: Figure S15c, d). Thus, these results demonstrated that CDKN1B and ING5 exerted different functions during miR-196a-mediated cisplatin resistance in HNC cells.

### Exosomal miR-196a promotes cisplatin resistance in HNC cells through CDKN1B and ING5 downregulation

**Fig. 7:**
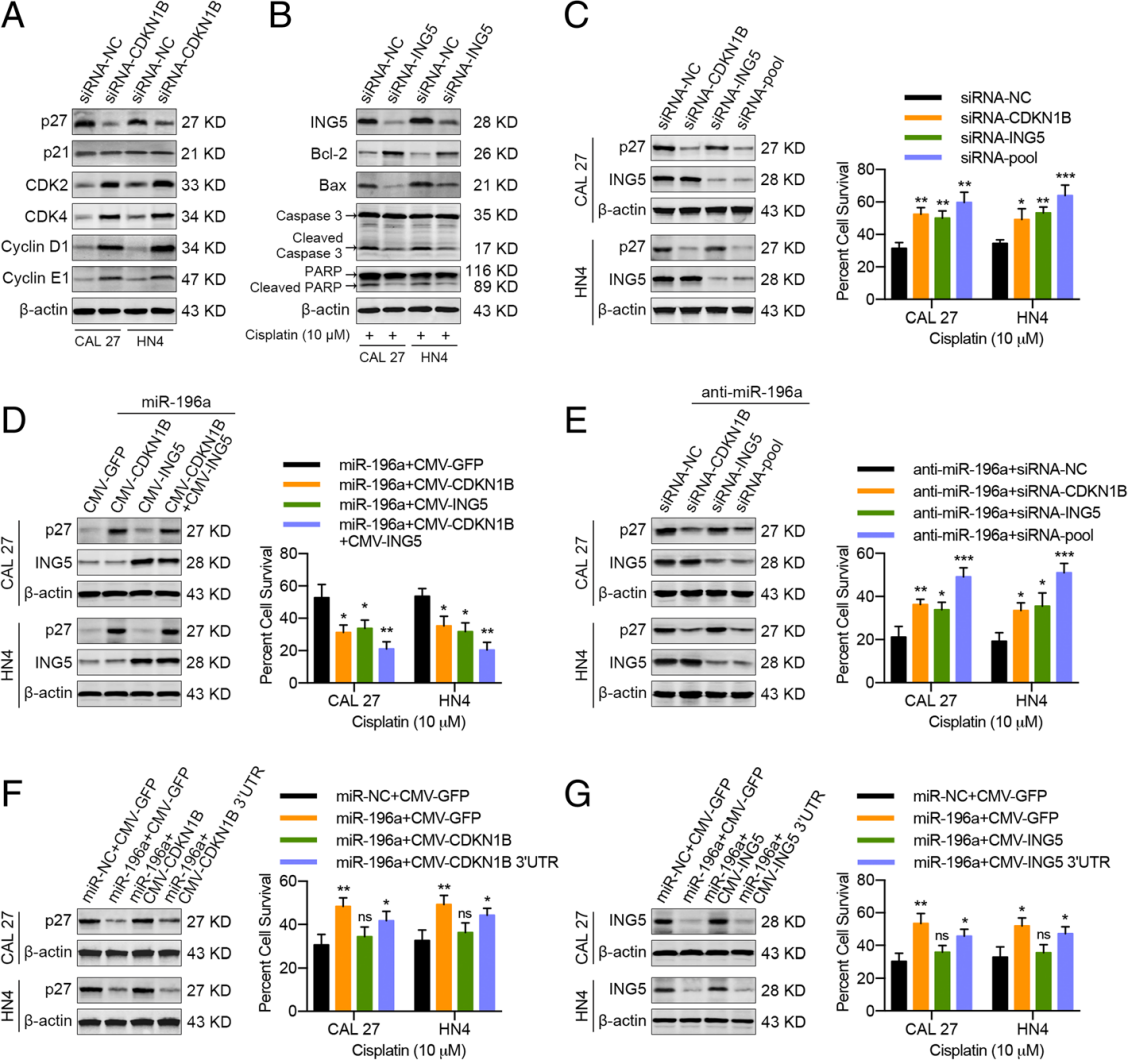
miR-196a accelerates cisplatin resistance in HNC cells via CDKN1B and ING5 downregulation. **a** Western blot showing p27, p21, CDK2, CDK4, Cyclin D1, and Cyclin E1 protein levels in CAL 27 and HN4 cells at 48 h after transfection with siRNA specific for CDKN1B. **b** Western blot showing ING5, Bcl-2, Bax, full-length Caspase 3, cleaved Caspase 3, full-length PARP, and cleaved PARP protein levels in CAL 27 and HN4 cells at 48 h after transfection with siRNA specific for ING5. **c** Left: western blot showing p27 and ING5 protein levels in CAL 27 and HN4 cells at 48 h after transfection with siRNAs specific for CDKN1B or ING5. Right: MTT assay showing the cisplatin response of CAL 27 and HN4 cells at 48 h after transfection, as indicated. **d** Left: western blot showing p27 and ING5 protein levels in CAL 27 and HN4 cells at 48 h after concurrent transfection with miR-196a and an exogenous expression vector (CDKN1B or ING5). Right: cisplatin response in CAL 27 and HN4 cells at 48 h after transfection, as indicated. **e** Left: western blot showing p27 and ING5 protein levels in CAL 27 and HN4 cells at 48 h after transfection with anti-miR-196a and siRNAs specific for CDKN1B or ING5. Right: cisplatin response of CAL 27 and HN4 cells at 48 h after transfection, as indicated. **f** Left: western blot showing p27 protein levels in CAL 27 and HN4 cells at 48 h after transfection with miR-196a and an exogenous expression vector (CDKN1B or CDKN1B 3′ UTR). Right: cisplatin resistance in CAL 27 and HN4 cells at 48 h after transfection, as indicated. **g** Left: western blot showing ING5 protein levels in CAL 27 and HN4 cells at 48 h after transfection with miR-196a and an exogenous expression vector (ING5 or ING5 3′ UTR). Right: cisplatin resistance in CAL 27 and HN4 cells at 48 h after transfection, as indicated. (ns, no significant difference; *p < 0.05; **p < 0.01; ***p < 0.001)

To further elucidate the functional role of the CDKN1B gene in miR-196a-mediated cell cycle transition, CAL 27 and HN4 cells were transiently transfected with specific siRNA targeting CDKN1B, and then western blotting analysis was performed. As a result, the p27 protein level was decreased, and CDK2, CDK4, Cyclin D1, and Cyclin E1 levels were increased in transfected HNC cells (Fig. 7a). Furthermore, we also analyzed the effective role of the ING5 gene in cell apoptosis via the transfection of ING5-targeting siRNA into HNC cells. As shown in Fig. 7b, ING5, Bax, Cleaved Caspase 3, and Cleaved PARP protein levels were drastically decreased, and the Bcl-2 level was significantly upregulated in transfected HNC cells. Similar results were obtained when CAL 27 and HN4 cells were treated with CAF-derived exosomes (Additional file 1: Figure S19a, b). Meanwhile, p27 or ING5 protein knockdown by siRNA significantly endowed parental cells with cisplatin resistance (Fig. 7c). Moreover, simultaneous transfection with miR-196a and plasmids allowing the expression of complementary DNA lacking the endogenous 3′ UTR (CMV-CDKN1B and CMV-ING5) significantly reduced the miR-196a-mediated promotion of cisplatin resistance (Fig. 7d). Similar results were obtained when HNC cells were transfected with CDKN1B- or ING5-expressing plasmids without 3′ UTR and then treated with CAF-derived exosomes (Additional file 1: Figure S19c). Conversely, concurrent CDKN1B and ING5 knockdown in HNC cells abolished anti-miR-196a-induced chemoresistance inhibition, while knockdown of each protein alone had a limited effect (Fig. 7e). To further elucidate the function of miR-196a-induced cisplatin resistance through CDKN1B repression, CMV-CDKN1B and CMV-CDKN1B 3′ UTRs were transfected into miR-196a-overexpressing cells. As expected, western blotting revealed that CDKN1B was significantly downregulated upon miR-196a transfection but was rescued after transfection with CMV-CDKN1B but not CMV-CDKN1B 3′ UTR. Functionally, the promoting effects of miR-196a on cisplatin resistance were abolished by ectopic CMV-CDKN1B expression but were not substantially affected by CMV-CDKN1B 3′ UTR transfection (Fig. 7f). In addition, a similar phenomenon was observed when investigating the function of miR-196a-induced cisplatin resistance through ING5 repression (Fig. 7g). Collectively, these results suggested that exosomal miR-196a promoted cisplatin resistance in HNC cells through CDKN1B and ING5 downregulation.

### CAF-derived exosomal miR-196a promotes HNC cell cisplatin resistance in vivo

**Fig. 8:**
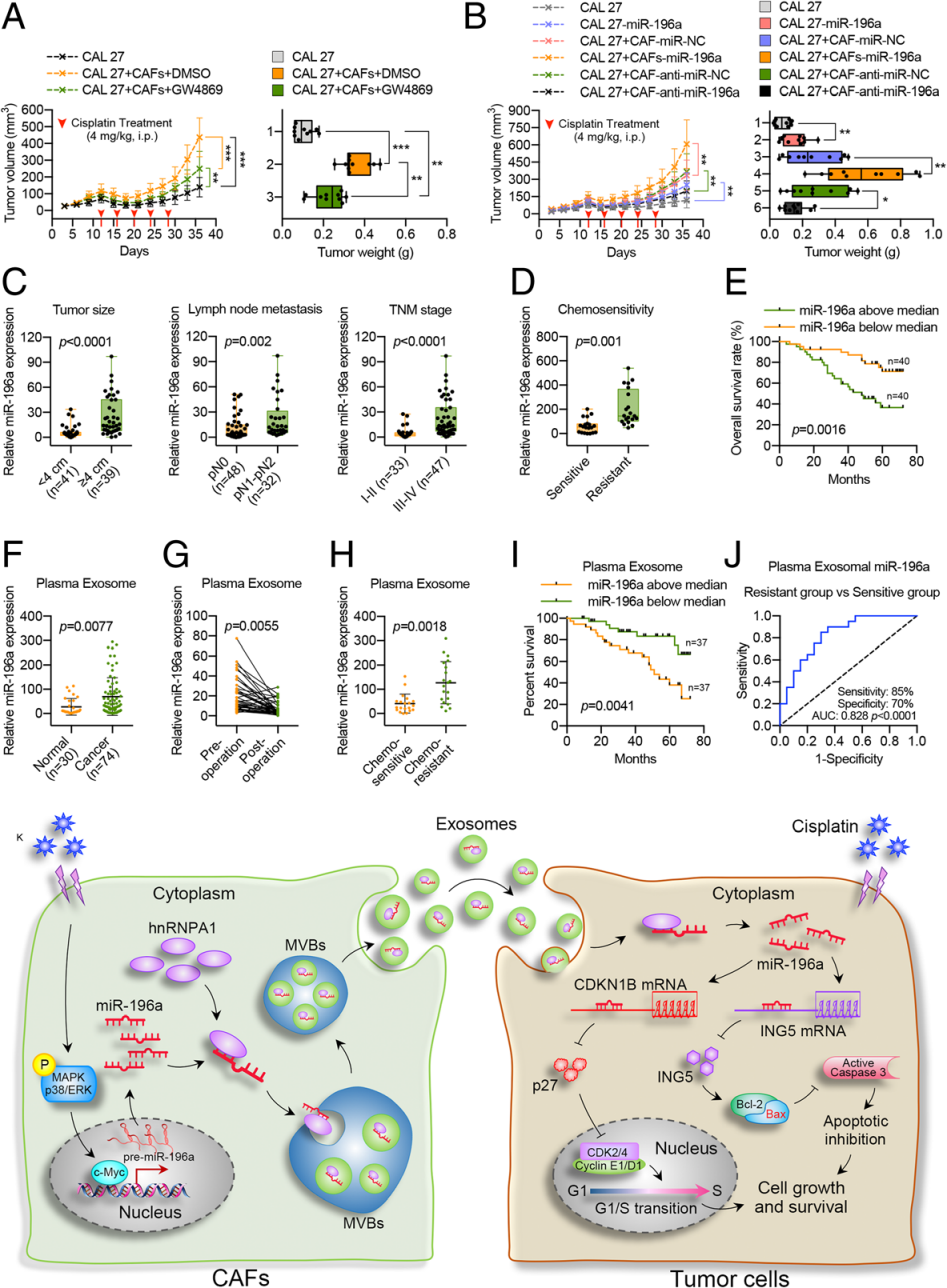
CAF-derived exosomal miR-196a enhances cisplatin resistance in HNC cells in vivo. **a** CAL 27 cells with or without CAFs were used to establish a xenograft mouse model. The mice were intraperitoneally injected with cisplatin (4 mg/kg, every 4 days) with or without GW4869 (2 mg/kg, every 2 days). The tumor growth curve, tumor volumes, and tumor weights are shown. **b** CAL 27 cells were stably transfected with or without miR-196a, and CAFs were stably transfected with miR-196a or anti-miR-196a. Nude mice were subcutaneously xenografted with pre-transfected CAL 27 cells or CAFs as indicated. The mice were intraperitoneally injected 5 times with cisplatin (4 mg/kg, every 4 days). The tumor growth curve, tumor volumes, and tumor weights are shown. **c** Upregulated miR-196a levels were correlated with increased tumor size, lymph node metastasis, and advanced tumor stage in HNC tissues. **d** Real-time PCR analysis showing miR-196a expression in HNC tissues from chemosensitive patients (*n* = 20) and chemoresistant patients (*n* = 20). **e** Kaplan-Meier analysis of overall survival. Compared with patients with low miR-196a expression, patients with high miR-196a expression had a significantly lower overall survival rate. **f** Plasma exosomal miR-196a levels were detected using real-time PCR with plasma from HNC patients and healthy donors. **g** Real-time PCR analysis of exosomal miR-196a in matched plasma from HNC patients pre- and postoperation (*n* = 40). **h** Real-time PCR analysis of exosomal miR-196a expression in the plasma of chemosensitive patients (*n* = 20) and chemoresistant patients (*n* = 20). **i** Kaplan-Meier analysis of overall survival in 74 HNC patients from high and low miR-196a groups, according to the median exosomal miR-196a level in pre-therapy plasma. **j** ROC curve analysis of plasma exosomal miR-196a expression for discriminating the chemoresistant group (*n* = 20) from the chemosensitive group (*n* = 20). AUC, area under the curve. **k** A proposed model illustrating the modulatory role of CAF-derived exosomal miR-196a in regulating HNC cell proliferation and cisplatin resistance. (*p < 0.05; **p < 0.01; ***p < 0.001; ****p < 0.0001)

To determine whether CAF-derived exosomal miR-196a affected HNC cell tumorigenicity and cisplatin resistance in vivo, we established a xenograft model by subcutaneously injecting CAL 27 cells transfected with or without miR-196a and a mixture of CAFs and CAL 27 cells into the buttocks of nude mice. Twelve days after the injections, all mice received five intraperitoneal injections of 4 mg/kg cisplatin at 4-day intervals. The tumors formed by CAL 27 plus CAFs grew faster than those in the CAL 27 group upon cisplatin treatment, and GW4869 substantially reduced CAF-mediated cisplatin resistance in CAL 27 cells (Fig. 8a; Additional file 1: Figure S20a). These results revealed that CAF-derived exosomes were involved in CAF-mediated cisplatin resistance in HNC. Moreover, miR-196a-overexpressing CAL 27 cells were more tolerant to cisplatin treatment than CAL 27 cells (Fig. 8b; Additional file 1: Figure S20b), indicating that miR-196a could endow CAL 27 cells with cisplatin resistance in vivo. In addition, overexpression of miR-196a in CAFs promoted CAF-mediated cisplatin resistance in CAL 27 cells, whereas knockdown of miR-196a expression in CAFs reversed this effect (Fig. 8b; Additional file 1: Figure S20b). The above data demonstrated that CAF-derived exosomal miR-196a conferred cisplatin resistance in HNC. Interestingly, CDKN1B and ING5 expression were negatively correlated with miR-196a expression in xenograft tissues. As detected by real-time PCR, western blotting, and immunohistochemical staining, miR-196a expression was significantly upregulated in the CAL 27-miR-196a and CAL 27+CAFs groups and was partly reduced in the CAL 27+CAFs+GW4869 and CAL 27+CAF-anti-miR-196a groups. However, both CDKN1B and ING5 mRNA and protein levels exhibited the opposite trend as that of miR-196a (Additional file 1: Figure S21). Functionally, both miR-196a overexpression and incubation with CAFs increased the proportion of Ki-67-positive cells and reduced the number of apoptotic cells upon cisplatin treatment in xenograft tumors, while depletion of CAF-derived exosomes or exosomal miR-196a reversed these effects (Additional file 1: Figure S21c, d). These data suggested that CAF-secreted exosomal miR-196a could endow HNC cells with cisplatin resistance by downregulating CDKN1B and ING5 levels in vivo.

### miR-196a upregulation in tumor tissues and plasma indicates malignant transformation and correlates with chemoresistance in HNC patients

In addition to cisplatin resistance, our data also revealed that CAF-derived exosomal miR-196a endowed HNC cells with refractoriness to both adriamycin and paclitaxel (Additional file 1: Figure S22), suggesting that exosomal miR-196a played an important role in chemoresistance.

To examine whether miR-196a accounts for malignant transformation and chemoresistance in HNC, correlations between miR-196a expression and clinical features of HNC patients were analyzed. As shown in Additional file 1: Figure S6e and f, miR-196a levels were upregulated in HNC tissues compared with adjacent normal tissues. Further analysis revealed that miR-196a upregulation in HNC tissues was correlated with increased tumor size, lymph node metastasis, advanced tumor stage, and chemoresistance (Fig. 8c, d; Additional file 4: Table S3). Moreover, Kaplan-Meier and Cox regression analyses indicated that high miR-196a levels were significantly correlated with reduced overall survival in 80 HNC patients (Fig. 8e; Additional file 4: Table S4). In addition, CDKN1B and ING5 downregulation in HNC (Additional file 1: Figure S14c–e) was closely related to increased tumor size, lymph node metastasis, advanced tumor stage, and chemoresistance, which eventually contributed to the poor prognosis in HNC patients (Additional file 1: Figures S23, S24). Interestingly, the expression of CDKN1B or ING5 was negatively correlated with hnRNPA1 expression in HNC (Additional file 1: Figures S10e, f).

Since exosomal miR-196a was detected in the CAF-CM (Fig. 3e), we then explored whether exosomal miR-196a was present in HNC patient plasma. Fortunately, miR-196a was detected in plasma exosomal RNA. Plasma exosomal miR-196a levels were increased in HNC patients compared with healthy donors (Fig. 8f). Furthermore, plasma exosomal miR-196a levels decreased after tumor resection (Fig. 8g), indicating that plasma exosomal miR-196a was mainly produced by tumor tissue. Exosomal miR-196a levels were significantly higher in the plasma of chemoresistant patients than in that of chemosensitive patients (Fig. 8h). Similarly, positive associations were also identified between plasma exosomal miR-196a levels and increased tumor size, lymph node metastasis, and advanced tumor stage (Additional file 5: Table S5). Moreover, Kaplan-Meier analysis showed that high exosomal miR-196a levels in HNC patient plasma were correlated with reduced overall survival, and Cox proportional hazards regression analysis further demonstrated that high plasma exosomal miR-196a expression was an important prognostic factor in HNC patients (Fig. 8i; Additional file 5: Table S6). Importantly, receiver operating characteristic (ROC) curve analysis demonstrated that the ability to discriminate between the chemoresistant and chemosensitive group with the plasma exosomal miR-196a level was acceptably accurate (Fig. 8j). These data suggested that plasma exosomal miR-196a could serve as a valuable prognostic factor as well as an independent predictor for chemoresistance in HNC patients.

## Discussion

Patients suffering from HNC have an overall poor prognosis, owing to proliferation and chemoresistance. Chemotherapy has been widely accepted as a major therapy in advanced cancers, and cisplatin is still one of the first-line drugs for chemotherapeutic administration in HNC. Unfortunately, cisplatin resistance is becoming increasingly severe and remains one of the major impairments in HNC clinical therapy [3]. Therefore, investigating the molecular mechanisms underlying cisplatin resistance may be of great significance for improving HNC patient outcome. In addition to multiple reported mechanisms, such as drug efflux, increased DNA repair, and insensitivity to drug-induced apoptosis [3], recent studies have suggested that mechanisms of therapy resistance are largely conferred by alterations in the tumor microenvironment, not tumor cells [13, 14, 29, 30]. CAFs, the most abundant cells in tumor stroma, are not passive elements but key players that could potentially respond to and affect chemotherapy [29–31]. However, how CAFs modulate chemotherapy resistance in cancers, especially HNC, is not well understood. Focusing on CAFs as stromal drivers of chemoresistance, we aimed to investigate the mechanism underlying CAF-mediated cisplatin resistance in HNC. In this study, we showed that HNC-derived CAFs are innately resistant to cisplatin and determined that CAF-CM supported HNC cell growth and survival during cisplatin treatment. Moreover, HNC cells became insensitive to cisplatin after co-culturing with CAFs. Similar results were also obtained when HNC cells were treated with adriamycin or paclitaxel. Hence, we concluded that CAFs could endow HNC cells with refractoriness to chemotherapeutic drugs, but the detailed mechanisms remain elusive.

The mechanisms by which CAFs could regulate malignant behaviors, by secreting chemokines, cytokines, growth factors, etc., have already been described [8–10, 32]. Recent studies reveal that exosomes promote tumor progression and increase drug resistance in cancer cells by delivering oncogenic DNA, protein, and non-coding RNA [12, 19]. Most recent reports show that CAF-derived exosomes play a critical role in promoting chemoresistance in many cancers [19, 33], which is not be extensively evaluated in HNC. In our study, we proved that CAF-secreted exosomes promoted cisplatin resistance in HNC cells, and treating CAFs with cisplatin increased the release of chemoresistance-promoting exosomes. Considering the evidence that the HNC tumor mass is comprised mostly of stromal cells, it is possible that exosome-based therapies that target CAFs may have better curative effects. Our data indicated that the exosome secretion inhibitor GW4869 significantly alleviated CAF-mediated cisplatin resistance in HNC cells in vitro and suppressed tumor growth in vivo. However, further in vivo experiments should be implemented to fully explore the potential benefit of using GW4869 to increase the efficacy of combined treatments.

The development of chemotherapy resistance in tumor cells can be promoted by exosomes through a variety of mechanisms. Tumor-derived exosomes can transfer multidrug resistance-associated proteins, miRNAs, and lncRNAs to recipient cells [34, 35]. Recent evidence revealed that exosome-mediated miR-21 and miR-146a delivery from CAFs to tumor cells conferred chemoresistance in recipient tumor cells [14, 15, 20]. miRNAs are short (19–25 nucleotides) non-coding RNAs that negatively regulate target gene expression and participate in a variety of biological processes [33, 36]. Importantly, miRNA deregulation can promote drug resistance in tumors with different origins [19, 33]. Unsurprisingly, exosomal miRNAs may play a role in mediating resistance transfer from CAFs to tumor cells. In this study, we identified CAF-derived exosomal miR-196a as the functional molecule that conferred cisplatin resistance to HNC cells. Furthermore, we also found that miR-196a could promote cell growth and inhibit cell apoptosis upon cisplatin treatment in HNC. miR-196a upregulation is reportedly correlated with increased proliferation and chemoresistance in other cancers (such as cervical cancer, gastric cancer, and bladder cancer) [37–40], which is in accordance with our data. Importantly, we further investigated how miR-196a was packed into exosomes. In exosomes, RBPs such as hnRNPQ and hnRNPA2B1 have been shown to be involved in exosomal miRNA export by binding specific motifs [41, 42]. Apart from hnRNPs, a relevant function in miRNA trafficking has been proven for Ago2 and Y-box protein 1 [43, 44]. Interestingly, a third regulatory mechanism of exosomal miRNA export has been identified as lncRNA-miRNA interactions [35], which are similar to miRNA sponges. In the present study, we revealed that hnRNPA1 specifically bound miR-196a through a specific sequence (UAGGUA) on the 5′ end of miR-196a, directed miR-196a packaging into exosomes, which might provide a mechanism for eliminating miR-196a in the HNC stroma niche during cancer therapy. However, some other functional factors existing in exosomes may also contribute to cisplatin resistance in HNC cells, and how miR-196a is packaged into exosomes has not been fully elucidated. Therefore, in the future, further studies should be carried out to better illustrate these problems.

Exosome transport is believed to be an effective means to modulate cell signaling and biological function in recipient cells. Exosomal miR-196a functionally regulates proliferative and apoptotic abilities by targeting CDKN1B and ING5 after being delivered from CAFs to HNC cells. CDKN1B protein, also known as p27, is a potent inhibitor of cyclin-dependent kinases that drive G1/S transition. The p27 protein has been suggested to be a tumor suppressor with a haploinsufficiency phenotype, and in the absence of p27, CDK2 activity was increased [45]. Reduced p27 expression is prevalent in a wide range of human tumors, which is associated with poor prognosis and drug resistance [46]. ING5, which acts as a class II tumor suppressor, was downregulated in many cancers and was able to suppress cell growth and proliferation, induce apoptosis, and negatively modulate drug resistance by regulating multiple signaling pathways [47, 48]. ING5 proteins are involved in transcriptional regulation of genes, such as the p53-inducible genes p21 and Bax [48]. ING5 has also been found to affect post-translational modifications and help maintain genomic stability upon radiation or chemotherapy [49]. Consistent with these results, our results revealed that CDKN1B and ING5 levels were downregulated in HNC, which were correlated with tumor growth, lymph node metastasis, advanced tumor stage, and chemoresistance. Furthermore, we also demonstrated that the miR-196a-mediated G1/S cell cycle transition was regulated mainly by p27, whereas ING5 predominantly performed its function through modulating cell apoptosis. Considering the sophisticated functions of ING5, more efforts should be taken to elucidate the precise mechanism underlying ING5-mediated chemoresistance, aside from apoptosis inhibition, in future studies.

Liquid biopsy, which refers to the molecular analysis of disease genetic phenotypes based on circulating genetic material in body fluids, is already used to monitor the disease response and to track mechanisms of drug resistance in solid tumors, including HNC [50–52]. Exosomes act as key mediators of intercellular communication by transporting functional cargo among neighboring cells or by traveling to distant cells through biological fluids, where exosomes can be detected [52]. Plasma exosomal miRNAs, a new liquid biopsy source in free form, are promising diagnostic and prognostic biomarkers for many diseases, especially cancers [51, 52]. In this study, we confirmed that miR-196a overexpression in HNC tissues contributed to exosomal miR-196a upregulation in HNC patient plasma. Similar to miR-196a levels in HNC tissues, the upregulation of plasma exosomal miR-196a levels was positively correlated with malignant phenotypes, including chemoresistance, in HNC. Moreover, plasma exosomal miR-196a also exhibited favorable accuracy in prognosis and predicting chemoresistance. Therefore, we concluded that plasma exosomal miR-196a might serve as a valuable prognostic factor and an independent predictor for chemoresistance in HNC patients. However, the clinical prognostic and predictive efficiency of plasma exosomal miR-196a needs to be further verified in expanded HNC cohorts, and further studies should be implemented to investigate if any other exosomal miRNAs in the plasma also predict chemoresistance in HNC.

## Conclusions

Our results reveal that CAFs are innately resistant to cisplatin, and exosomes from CAFs exposed to cisplatin can confer chemoresistance and an aggressive phenotype in HNC cells through the transfer of functional miR-196a. Importantly, our data also revealed that the hnRNPA1 protein mediates miR-196a packaging into exosomes in CAFs. Moreover, our study highlights the role of CAF-derived exosomal miR-196a in promoting cell proliferation and inhibiting cell apoptosis by targeting CDKN1B and ING5 in the HNC microenvironment. Simultaneously, exosome or exosomal miR-196a depletion from CAFs could functionally restore the cisplatin response in HNC cells. In addition, we identified that plasma exosomal miR-196a is correlated with poor overall survival and drug sensitivity in the clinic. Therefore, miR-196a may serve as a promising predictor and potential therapeutic target for cisplatin resistance in HNC.

## Methods

### Ethics

The Ethics Committee of Shanghai Jiao Tong University ratified our study. Written informed consents were provided by the participants prior to enrollment. All experimental methods abided by the Helsinki Declaration. All animal studies were undertaken in accordance with the NIH Guide for the Care and Use of Laboratory Animals, with the approval of the Shanghai Jiao Tong University Institute Animal Care and Use Committee, and the experimental mice were raised in the Shanghai Jiao Tong University School of Medicine animal facilities.

### Patients and specimens

All the clinical samples were collected from the Department of Oral and Maxillofacial-Head and Neck Oncology, Ninth People’s Hospital, Shanghai Jiao Tong University School of Medicine (Shanghai, China). More specifically, 80 pairs of tumor and adjacent normal tissues were obtained from the patients who were diagnosed with primary HNC and underwent initial surgery between September 2011 and June 2015, and another 28 pairs were collected between July 2018 and September 2018 (no complete prognostic information). These patients had not received any systemic treatment before sampling. Half of each sample was quickly frozen in liquid nitrogen until the extraction of total RNA and protein, and the other half was sent for pathologic diagnosis. Between June 2011 and August 2016, we recruited 74 patients with primary HNC, collected their plasma samples before surgery, and stored at − 80 °C until further processing. In another 40 HNC patients, plasma samples were collected 1 day before surgery and 3 days after tumor resection. Moreover, plasma samples from 30 donors of physical examination in the Ninth People’s Hospital were selected as healthy controls. In this study, none of these HNC patients received any preoperative cancer treatment and were histologically diagnosed as HNC after the operation. In parallel, tumor tissues were collected from biopsy or surgical specimens of advanced HNC patients who were sensitive (*n* = 20) or resistant (*n* = 20) to chemotherapy. We also retrospectively reviewed the medical records and follow-up data of these HNC patients. Additionally, pathological differentiation and clinical stage were respectively determined according to World Health Organization Classification of Tumors and the tumor node metastasis (TNM) staging system (2010) from the Union for International Cancer Control (UICC).

### Cell cultures

SCC-4, SCC-9, SCC-25, CAL 27, and 293T cells were purchased from the American Type Culture Collection (ATCC, USA), and the human HNC cell lines HN4, HN6, and HN30 were kindly provided by the University of Maryland Dental School, USA. The cisplatin-resistant cell line HN4-res was established from HN4 cells by incubating with a concentration gradient of cisplatin for 1 year and maintained in a culture medium containing 15 μM cisplatin (Sigma-Aldrich, USA). As previously described [9], CAFs and primary cancer cells were isolated from HNC tissues by primary culture while NFs were derived from the paired adjacent normal tissues. The paired NFs and CAFs were further identified by the presence of CAF-specific markers (α-SMA, FAP, and FSP1). SCC-4, SCC-9, and SCC-25 cells were cultured in Dulbecco’s modified Eagle medium/nutrient mixture F-12 (DMEM/F-12; GIBCO-BRL, USA) supplemented with 10% fetal bovine serum (FBS; GIBCO-BRL, USA), penicillin (100 units/mL), and streptomycin (100 μg/mL) at 37 °C in a humidified 5% CO_2_ atmosphere, whereas other cells were maintained in DMEM medium containing the same additives. In addition, normal primary oral epithelial cells were cultured in keratinocyte serum-free medium (KSF; GIBCO-BRL, USA) with 0.2 ng/mL recombinant epidermal growth factor (rEGF; Invitrogen, USA).

### Immunofluorescence

Cells (3 × 10^4^ cells per well) grown on cover slips were fixed with 4% paraformaldehyde, permeabilized with 0.1% Triton X-100, blocked in 3% BSA, and incubated with primary antibodies overnight at 4 °C. Thereafter, the samples were incubated with an Alexa Fluor 488-conjugated anti-mouse IgG F(ab′)2 fragment (Invitrogen, USA; 1:200), an Alexa Fluor 549-conjugated anti-rabbit IgG F(ab′)2 fragment (Invitrogen, USA; 1:200), or an Alexa Fluor 488-conjugated anti-rabbit IgG F(ab′)2 fragment (Invitrogen, USA; 1:200) at room temperature for 30 min in the dark, and were further developed by treating with 4′,6-diamidino-2-phenylindole (DAPI; Invitrogen, USA; 1:300) to detect nuclei. Cells were observed and imaged using a TCS SP2 laser-scanning confocal microscope (Leica Microsystems, Germany), see Additional file 6: Table S7 for antibodies used.

### Western blot analysis

Cells or exosomes were harvested at the indicated times, prepared with SDS lysis buffer (Beyotime, China), and centrifuged at 14,000×*g* for 10 min at 4 °C. Proteins (30 μg) were separated using 10% or 15% polyacrylamide gels and transferred onto 0.22-μm PVDF membranes (Merck Millipore, USA). The blots were blocked with 5% BSA for 1 h at room temperature and incubated with primary antibodies overnight at 4 °C. The protein β-actin was used throughout as a loading control. Thereafter, the membranes were probed by IR Dye-labeled secondary antibodies and the signals were observed using an Odyssey Infrared Imaging System (Biosciences, USA), see Additional file 6: Table S7 for antibodies used.

### MTT assay

Cell viability was assessed by MTT assay. For drug response of HNC cells, tumor cells were pretreated as indicated and then seeded in a 96-well plate at a density of 3000 cells in each well in sextuplicate. Twelve hours later, the cells were incubated with a gradient concentration of therapeutic drugs for 72 h. The cells were incubated with 100 μL of 0.5 mg/mL 3-(4,5-dimethylthiazol-2-yl)-2,5-diphenyltetrazolium bromide (MTT; Sigma-Aldrich, USA) in DMEM medium for 4 h. The formazan that formed was then solubilized by adding 150 mL of dimethyl sulfoxide (DMSO). Absorbance was read at 490 nm in a multi-well plate reader (Bio-Rad Laboratories, Hercules, CA, USA). The degree of drug response for tumor cells was estimated by dividing the half maximal inhibitory concentration (IC50).

For the cell proliferative ability of HNC cells, tumor cells were seeded in a 96-well plate at a density of 1000 cells in each well in triplicate after pretreatment. During the co-culture period, the tumor cell growth was monitored daily by reading the absorbance at 490 nm.

### CM preparation

About 2 × 10^6^ donor cells were plated in a culture dish with a diameter of 10 cm. Twenty-four hours later, the culture medium was replaced with serum-free DMEM and incubated for 48 h. For the CM from cisplatin-treated cells, the cells were incubated with serum-free DMEM containing 10 μM cisplatin. The donor medium was spun down at 3000×*g* for 10 min and stored at 4 °C. For long-term treatment of cells, the prepared CM was supplemented with 2% exosome-free FBS (SBI, USA). To obtain exosome-free CM, the CM was spun down successively at 300×*g* for 20 min, 2000×*g* for 20 min, and 12,000×*g* for 70 min to deplete exosomes from the media.

### Exosome isolation

For exosome purification, CM was pre-cleared by filtration through a 0.22 μm PVDF filter (Millipore, USA). Exosomes were isolated from the CM by differential centrifugation steps as previously described [53]. The size and concentration of the exosomes were quantified using NanoSight NS300 instrument (Malvern Instruments Ltd., UK) equipped with NTA 3.0 analytical software (Malvern Instruments Ltd., UK). In addition, the plasma exosomes were isolated using ExoQuick Plasma prep and Exosome precipitation kit (SBI, USA). For exosomal RNA and protein extraction, exosomes were pretreated with RNase or Proteinase K, respectively. The exosome fraction protein content was assessed by Pierce® BCA Protein Assay kit (Thermo Scientific, USA).

### Transmission electron microscopy

For electron microscopy analysis, exosomes were dropped onto the copper grid and negatively stained by 2% phosphotungstic acid for 2 min. After air-drying for 15 min, the samples were observed under a transmission electron microscope (FEI Tecnai G2 Spirit, Thermo Scientific, USA) at 80 kV.

### Fluorescent labeling and transfer of exosomes

The isolated exosomes were labeled with the lipophilic dye DiO (Thermo Scientific, USA) at a working concentration of 10 μM and incubated for 20 min at 37 °C. Labeled exosomes were washed with PBS and centrifuged again at 12000×*g* for 70 min at 4 °C. The HNC cells (3 × 10^4^ cells per well) grown on cover slips were incubated with DiO-labeled exosomes for 24 h at a concentration of 25 μg/mL in a 24-well format. To identify the transfer of exosomal miR-196a, Cy3-labeled miR-196a was transfected to CAFs. The Cy3-miR-196a-expressing CAFs were then co-cultured with HNC cells for 48 h using a transwell chamber in a 24-well format. The cells were then prepared for immunofluorescence as described above. In addition, the cytoskeleton of HNC cells was selectively stained with TRITC Phalloidin (YEASEN, China) or FITC Phalloidin (YEASEN, China). Finally, the internalization of exosomes or exosomal miR-196a was measured by a confocal microscope.

For flow cytometry, HNC cells (2 × 10^5^ cells per well) were treated with DiO-labeled exosomes at a concentration of 25 μg/mL in a 6-well format. The cell samples were collected at 0 h, 1 h, 2 h, 4 h, 6 h, 12 h, and 24 h; resuspended in 500 μL PBS; and stained with DAPI. The presence of exosomes in HNC cells was assessed by capturing DiO signal using a flow cytometer (FACS Calibur, BD Biosciences, USA).

### Co-culture assay

The co-culture assay was established using transwell membranes (pores 0.4 μm, Merck Millipore, USA) in a 12-well format. CAFs were pretreated with GW4869 (Selleck, USA; 20 μM) or DMSO for 24 h in advance. The co-culture was performed for 3–6 days whereas CAFs (5 × 10^4^ cells) on the permeable membranes were treated with GW4869 (20 μM) or DMSO, and then HNC cells (5 × 10^4^ cells at the beginning) below the membranes were ready for RNA extraction or further cytological experiments.

### Plasmid construction

The cDNA of hnRNPA1, CDKN1B, CDKN1B 3′ UTR (with 3′ UTR), ING5, and ING5 3′ UTR (with 3′ UTR) was amplified by primeSTAR HS DNA polymerase (Takara, Japan) and subcloned into the EcoR I and Xho I sites of PGMLV-6395 vector (Genomeditech, China). To obtain the luciferase reporters, PCR-derived fragments from the 3′ UTR of CDKN1B and ING5 were inserted into the Xba I and BglI I sites of PGL3-CMV-LUC-MCS vector (Genomeditech, China). A PCR-based site-directed mutagenesis was performed to generate the mutant luciferase reporters. In addition, the miR-196a, anti-miR-196a, and hnRNPA1 shRNA plasmids were purchased from Genomeditech (China). Sequences of primers used for plasmid construction in this study were listed in Additional file 7: Table S8.

### Cell transfection

Transfection of plasmids was performed using Lipofectamine 3000 reagent (Invitrogen, USA) according to the manufacturer’s instructions. Transfection of siRNA (Genomeditech, China) or miRNA mimics or inhibitors (Ribobio, China) was performed using Lipofectamine RNAiMAX (Invitrogen, USA) at a final concentration of 100 nM. Sequences of siRNA against specific targets in this study were listed in Additional file 7: Table S9.

### Biotin miRNA pull-down assay

The detailed protocol was described in a previous study [42]. The NE-PERTM nuclear and cytoplasmic extraction reagents (Thermo Scientific, USA) were used to separate and prepare cytoplasmic and nuclear extracts from CAFs. Briefly, the nuclear, cytoplasmic, or exosomal lysates of CAFs were incubated overnight at 4 °C with 100 pmol of synthetic single-stranded miR-196a or mutated miR-196a oligonucleotides containing a biotin modification attached to the 5′ end via a spacer arm (Sigma-Aldrich, USA). Washed streptavidin agarose beads (Invitrogen, USA) were added to each binding reaction, which was further incubated at 4 °C for 4 h. Precipitates were washed five times and boiled in SDS buffer, followed by western blotting analysis. Biotinylated poly(G) (5′-GGGGGGGGGGGGGGGGGGGGG-3′) was used as a negative control.

### RNA extraction and real-time PCR analysis

Total RNA of cells or tissues was extracted by TRIzol reagent (Invitrogen, USA) and reverse-transcribed into cDNA using Prime Script RT reagent Kit (Takara, Japan). A miRcute Plus miRNA First-Strand cDNA Synthesis Kit (TIANGEN, China) was used to synthesize miRNA cDNA from total RNA of cells and tissues. The synthesized exogenous reference cel-miR-39 (1 pmol per sample; TIANGEN, China) was added into the culture medium (350 μL) or exosomes (100 μg) in advance, and miRNA in these samples was extracted by mirVana™ PARIS™ Kit (Ambion, USA). The real-time PCR for mRNA was performed using SYBR Premix Ex Taq Reagent Kit (Takara, Japan) with ABI StepOne Real-Time PCR System (Applied Biosystems, USA), whereas real-time PCR for miRNA was carried out by using miRcute Plus miRNA qPCR Detection Kit (TIANGEN, China). In cell and tissue lysates, mRNA levels were normalized against β-actin and miRNA levels were normalized against U6. Moreover, the miRNA levels in culture medium and exosomes were normalized against the exogenous reference cel-miR-39. Sequences of primers used for real-time PCR in this study were listed in Additional file 7: Table S10.

### RIP assay

RIP assays were performed using an EZ-Magna RIP RNA-Binding Protein Immunoprecipitation kit (Millipore, USA). Briefly, cells were collected and lysed in ice-cold lysis buffer supplemented with protease inhibitors, RNase inhibitors, and 1 mM PMSF. Lysates were centrifuged at 14,000×*g* for 15 min, and 50 μL of lysate was saved as input. The protein extract (1 mg) was incubated with 3 μg of rabbit anti-hnRNPA1 antibody (Cell Signaling Technology, USA) or rabbit IgG (Proteintech, USA) overnight at 4 °C with end-over-end rotation. Approximately 30 μL of A/G protein magnetic beads was then added and incubated at 4 °C for 4 h. Thereafter, the magnetic beads were washed five times, and coimmunoprecipitated miRNAs were extracted using a mirVana™ PARIS™ Kit (Ambion, USA). The isolated miRNA was reverse transcribed and then analyzed by real-time PCR. In addition, miRNA fold enrichment in immunoprecipitated samples is presented as percent input and compared with IgG isotypic control.

### Luciferase analysis

Briefly, 293T or tumor cells (3 × 10^4^ cells per well) grown in the 24-well plate were co-transfected with luciferase reporter (200 ng per well), miR-196a or anti-miR-196a plasmid (200 ng per well), and 10 ng Renilla luciferase vector (pRL-CMV; Genomeditech, China) using Lipofectamine™ 3000 (Invitrogen, USA). About 48 h later, a Dual-Luciferase Reporter Assay kit (Promega, USA) was used to measure the luciferase and renilla activity of these samples according to the manufacturer’s instructions.

### Immunoprecipitation of miRNA targets

Biotinylated miR-196a (Genomeditech, China) pull-down assay with target mRNAs was performed as described earlier [28]. Briefly, 5 × 10^6^ cancer cells cultured in 10-cm plates were transfected with 3′ biotin-labeled miR-NC or miR-196a for 48 h. The transfected cells were lysed with lysis buffer (20 mM Tris-HCl (pH 7.5), 100 mM KCl, 5 mM MgCl_2_, 0.3% IGEPAL CA-630, 60 U/mL Superase, 1× Protease Inhibitor) and harvested. A 50-μl aliquot of supernatant was taken as an input. Sample lysates were incubated with Streptavidin-Dyna beads (50 μl each sample; Invitrogen, USA) overnight at 4 °C on a rotator. Then, the beads were collected and washed with ice-cold lysis buffer five times. RNAs in the input and pull-down samples were isolated with a mirVana™ PARIS™ Kit (Ambion, USA), reverse transcribed, and then subjected to qRT-PCR. The analysis was done as follows: A = miRNA pull-down/control pull-down, B = miRNA input/control input, and fold enrichment = A/B.

### Tumorigenicity assay in vivo

A xenograft assay was performed to identify the effective role of hnRNPA1 in CAF-mediated HNC growth in vivo. A tumor-bearing model was constructed in BALB/C athymic nude mice (4 weeks old) by subcutaneously injecting a mixture of CAL 27 cells with hnRNPA1-expressing or hnRNPA1-silencing CAFs (5 × 10^5^ tumor cells plus 3 × 10^5^ CAFs per injection) into the left and right buttocks of the mice (five mice per group). Tumor volume and body weight were measured every 3 days. Approximately 24 days later, the mice were sacrificed, and the transplanted tumors were removed for subsequent analysis. The tumor growth curve was plotted using the tumor volume on the vertical axis and seeding day on the horizontal axis.

To evaluate the functional role of CAF-derived exosomal miR-196a in vivo, the experimental mice were separated into nine groups (*n* = 5): CAL 27 group, CAL 27+CAFs+DMSO group, CAL 27+CAFs+GW4869 group, CAL 27-miR-NC group, CAL 27-miR-196a group, CAL 27+CAF-miR-NC group, CAL 27+CAF-miR-196a group, CAL 27+CAF-anti-miR-NC group, and CAL 27+CAF-anti-miR-196a group. Thereafter, tumor cells (1 × 10^6^ cells per point) or mixed cells (5 × 10^5^ tumor cells plus 3 × 10^5^ CAFs per point) in serum-free DMEM were subcutaneously injected into the left and right buttocks of the mice. For the CAL 27+CAFs+GW4869 group, CAFs were pretreated with GW4869 (20 μM) for 24 h before injection, and mice were intraperitoneally injected with GW4869 (2 mg/kg) every 2 days. Twelve days after injection, the mice received five intraperitoneal injections of 4 mg/kg cisplatin at 4-day intervals, and the mice were sacrificed at the 32nd day for further analysis.

### Statistical analyses

Statistical analyses in this study were performed with SPSS 19.0 software. The significance between two or more groups was analyzed selectively by Student’s *t* test or one-way ANOVA. Mann-Whitney *U* tests and Kruskal-Wallis tests were used to analyze the association between gene (miR-196a, hnRNPA1, CDKN1B, and ING5) levels and clinical parameters. Pearson correlation analysis was performed to determine the correlation between two variables. The patients were divided into high expression group and low expression group according to the median of gene expression (miR-196a, hnRNPA1, CDKN1B, and ING5), and Kaplan-Meier survival analysis was implemented to compare HNC patient survival based on dichotomized gene expression by log-rank test. Cox proportional hazards regression analysis was utilized to evaluate the effect of clinical variables on patient survival. A general linear model was constructed to assess the synergistic effects of the different miRNAs. In general, data are shown as the means ± SD of three independent experiments in vitro, and *p* < 0.05 was considered statistically significant.

The detailed methods for the immunohistochemical analysis, lentivirus packaging, miRNA array analysis, EdU labeling assay, plate colony formation assay, cell cycle analysis, cell apoptosis analysis, CHIP assay, FISH assay and TUNEL assay are described in Additional file 8: Supplementary Methods.

## Additional files

Additional file 1: **Figures S1–S24.** with figure legends. (PDF 14700 kb)

Additional file 2: **Table S1.** Dysregulated miRNAs in CAF-derived exosomes compared with NFs. (DOC 53 kb)

Additional file 3: **Table S2.** Gene ontology analysis revealed 12 candidate genes for miR-196a whose altered expression could contribute to the chemoresistant phenotype. (DOC 40 kb)

Additional file 4: **Table S3.** Relationship between tissue miR-196a level and clinicopathologic features in HNC; Table S4. Cox proportional hazards regression models for estimating overall survival related to Table S3. (DOC 86 kb)

Additional file 5: **Table S5.** Relationship between plasma exosomal miR-196a level and clinicopathologic features in HNC; Table S6. Cox proportional hazards regression models for estimating overall survival related to Table S5. (DOC 86 kb)

Additional file 6: **Table S7.** Primary antibodies used in this study. (DOC 58 kb)

Additional file 7: **Tables S8–S10.** Sequences of primers and siRNAs used in this study. (DOC 152 kb)

Additional file 8: Supplementary methods. (DOC 54 kb)

## Abbreviations

CAFs: Cancer-associated fibroblasts
CM: Conditioned medium
DAPI: 4′,6-Diamidino-2-phenylindole
DiO: 3,3′-Dioctadecyloxacarbocyanine perchlorate
DMEM: Dulbecco’s modified Eagle medium
DMSO: Dimethyl sulfoxide
ELAVL1: ELAV-like protein 1
HNC: Head and neck cancer
hnRNPA1: Heterogeneous nuclear ribonucleoprotein A1
IC50: Half maximal inhibitory concentration
lncRNAs: Long non-coding RNAs
miRNAs: MicroRNAs
mRNAs: Messenger RNAs
MTT: 3-(4,5-Dimethylthiazol-2-yl)-2,5-diphenyltetrazolium bromide
MVBs: Multivesicular bodies
NFs: Normal fibroblasts
RBPs: RNA-binding proteins
RIP: RNA immunoprecipitation
ROC: Receiver operating characteristic
ZRANB2: Zinc finger ran-binding domain-containing protein 2
