# Supplementary material for: Exosomal miR-196a derived from cancer-associated fibroblasts confers cisplatin resistance in head and neck cancer through targeting CDKN1B and ING5
Source: Genome Biol. 2019 Jan 14;20:12. doi: 10.1186/s13059-018-1604-0 (PMC6332863; doi:10.1186/s13059-018-1604-0)
Supplement: Supplementary file 1 — Figures S1–S24. with figure legends. (PDF 14700 kb) [file 13059_2018_1604_MOESM1_ESM.pdf]

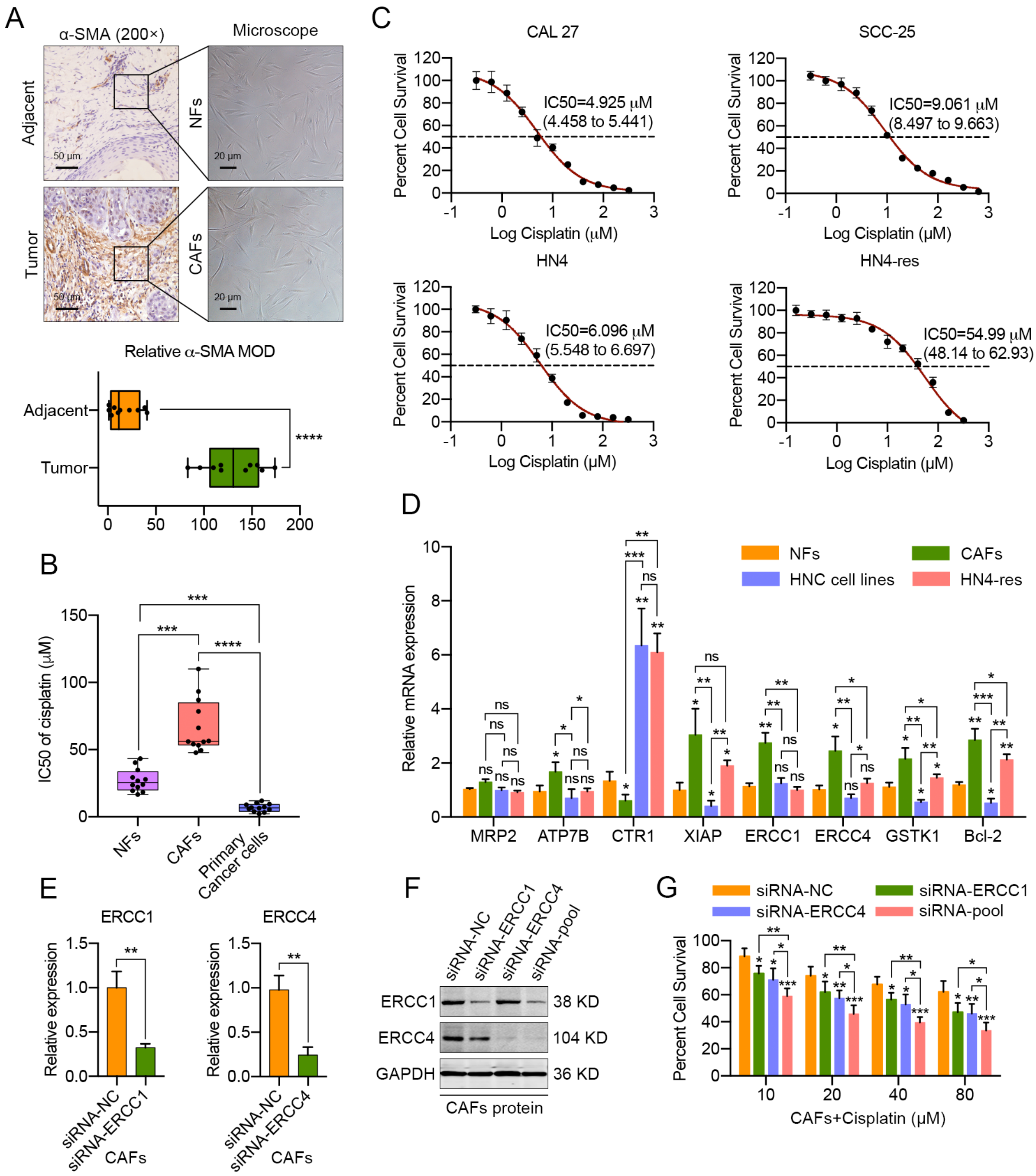

Supplementary Figure S1

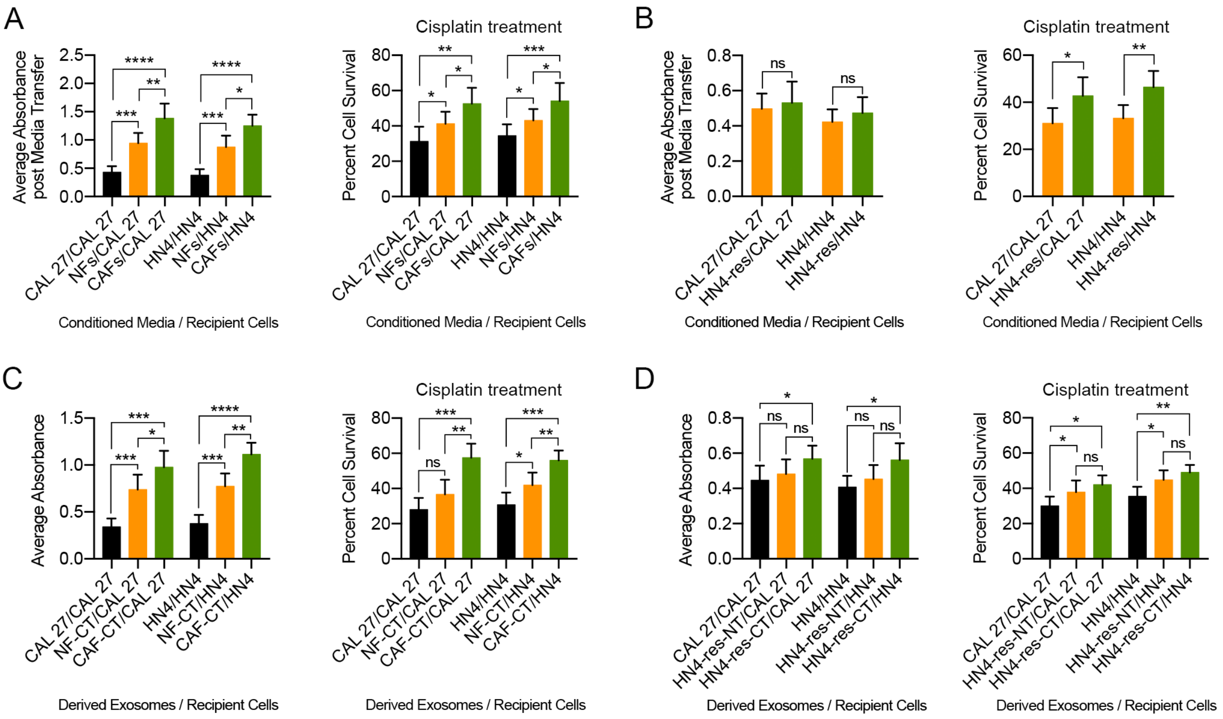

Supplementary Figure S2

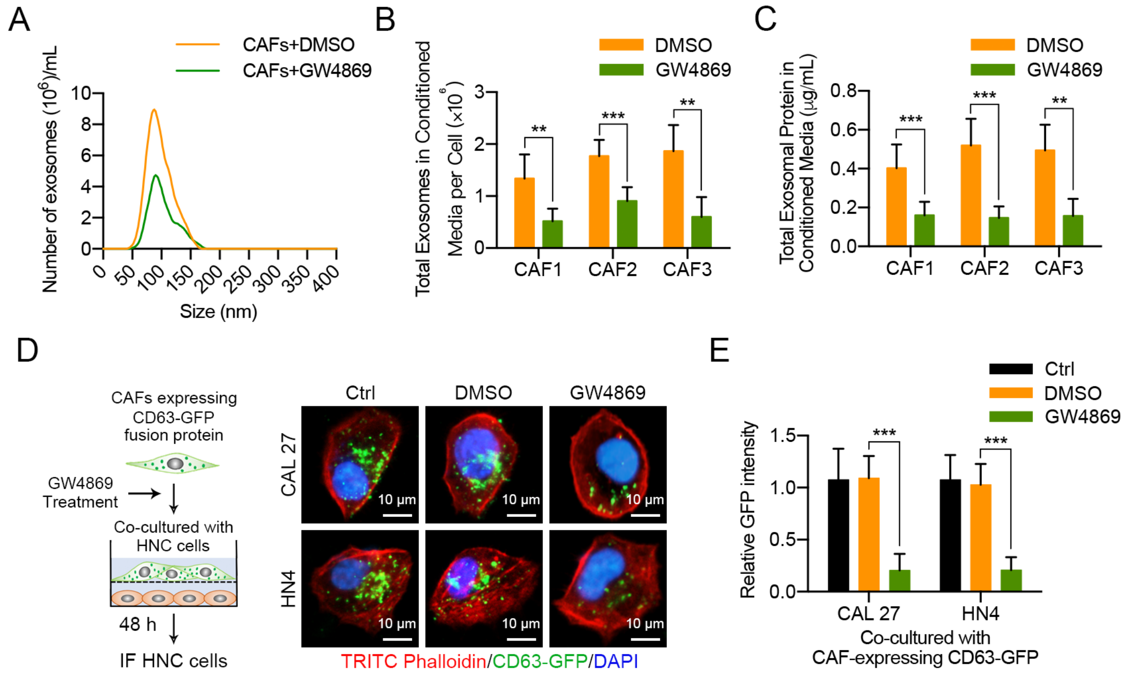

Supplementary Figure S3

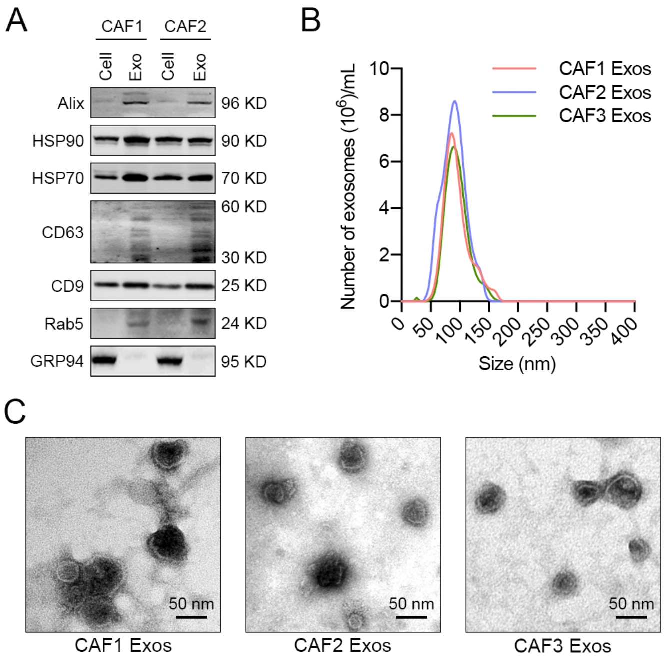

Supplementary Figure S4

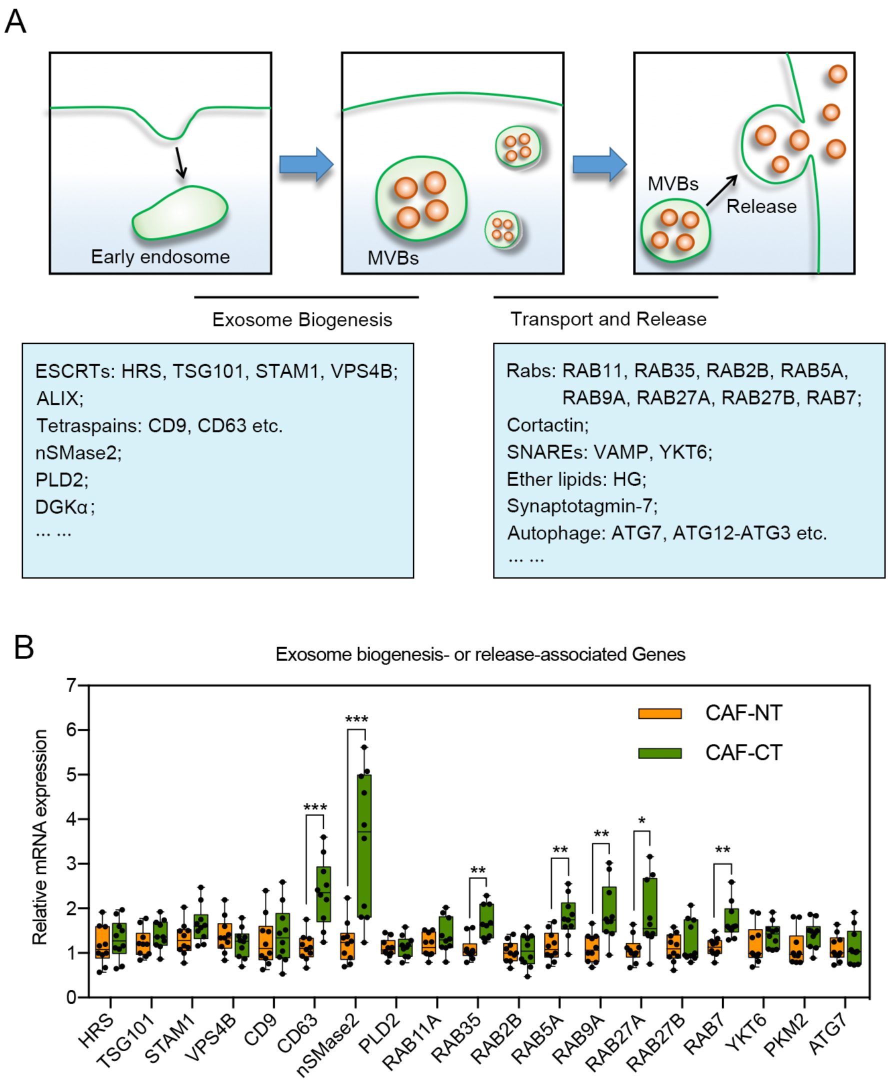

Supplementary Figure S5

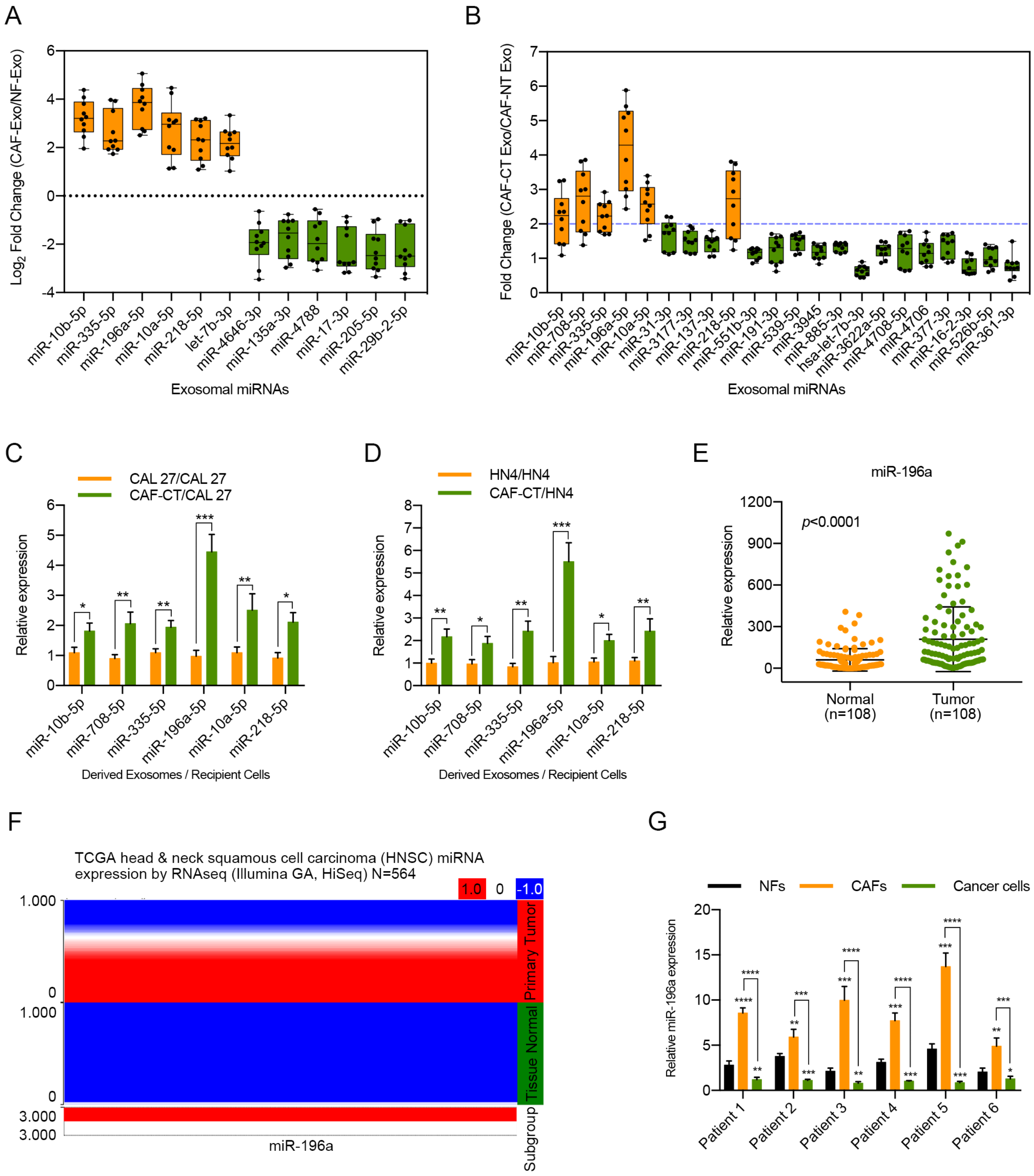

Supplementary Figure S6

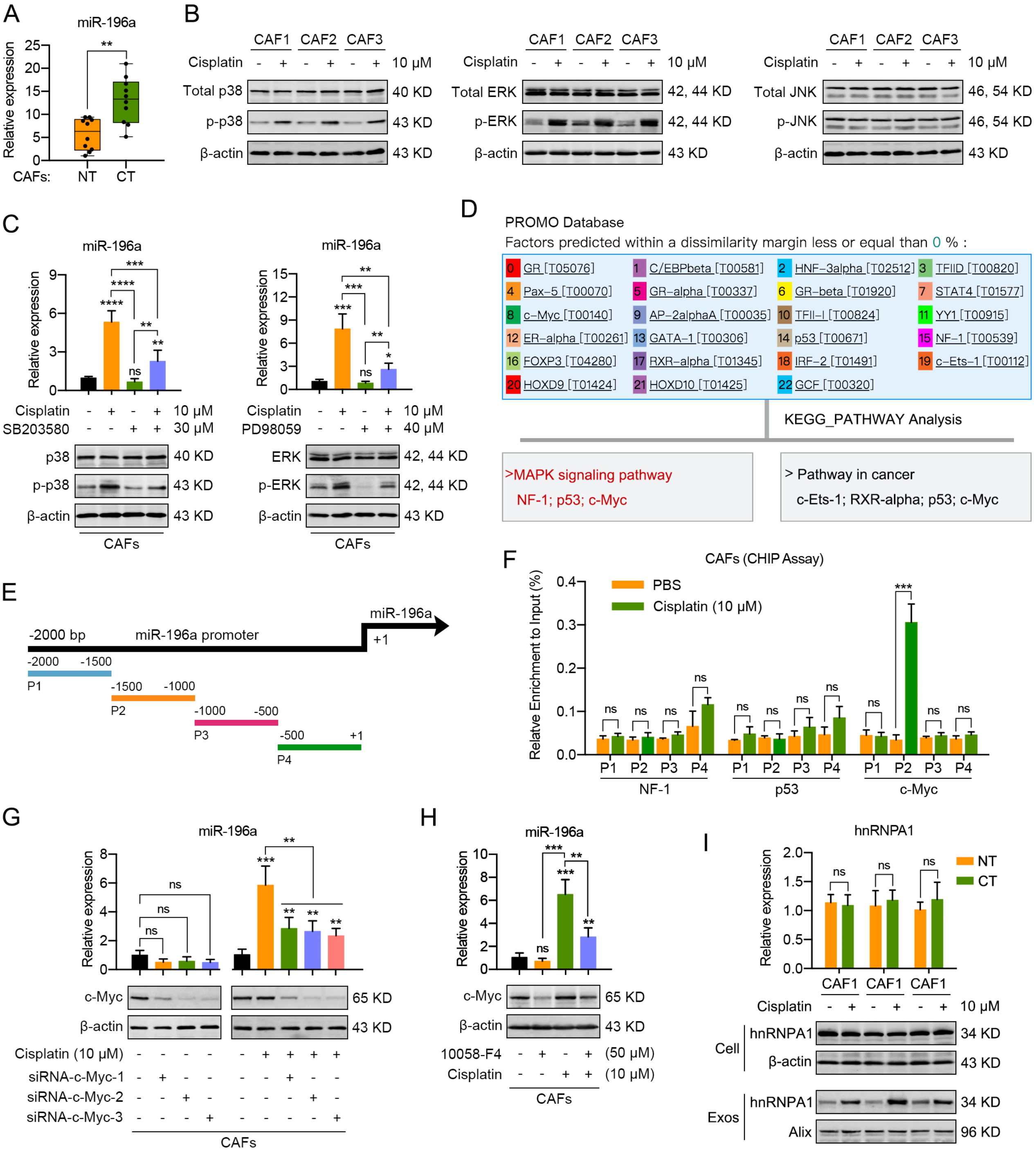

Supplementary Figure S7

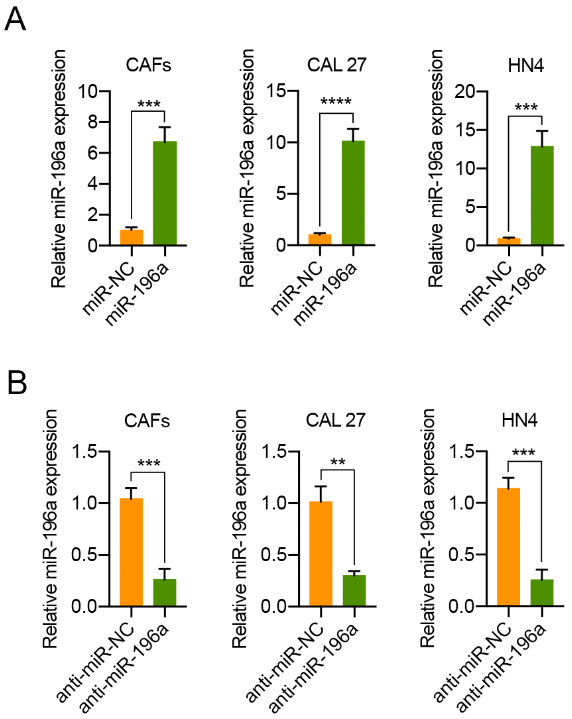

Supplementary Figure S8

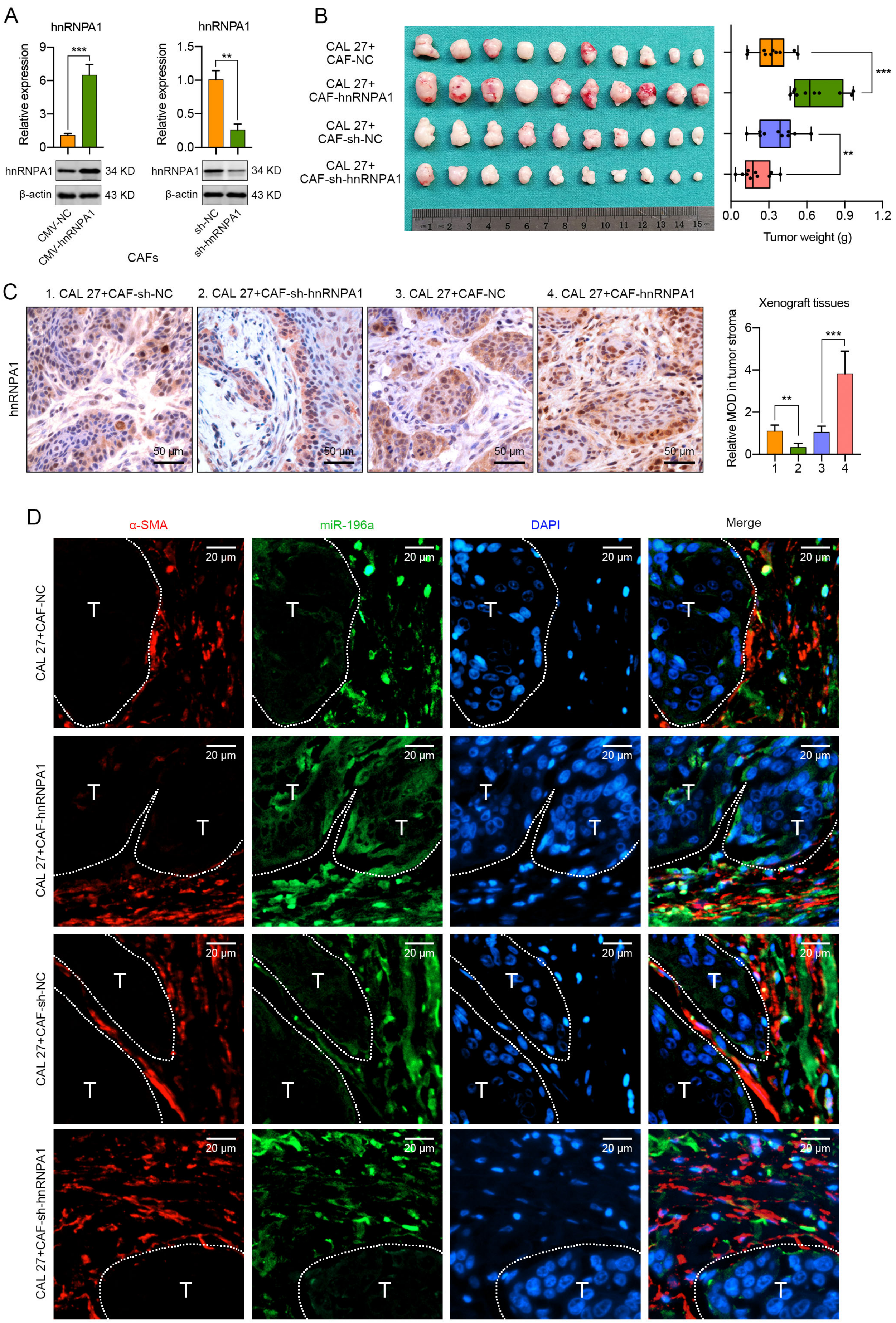

Supplementary Figure S9

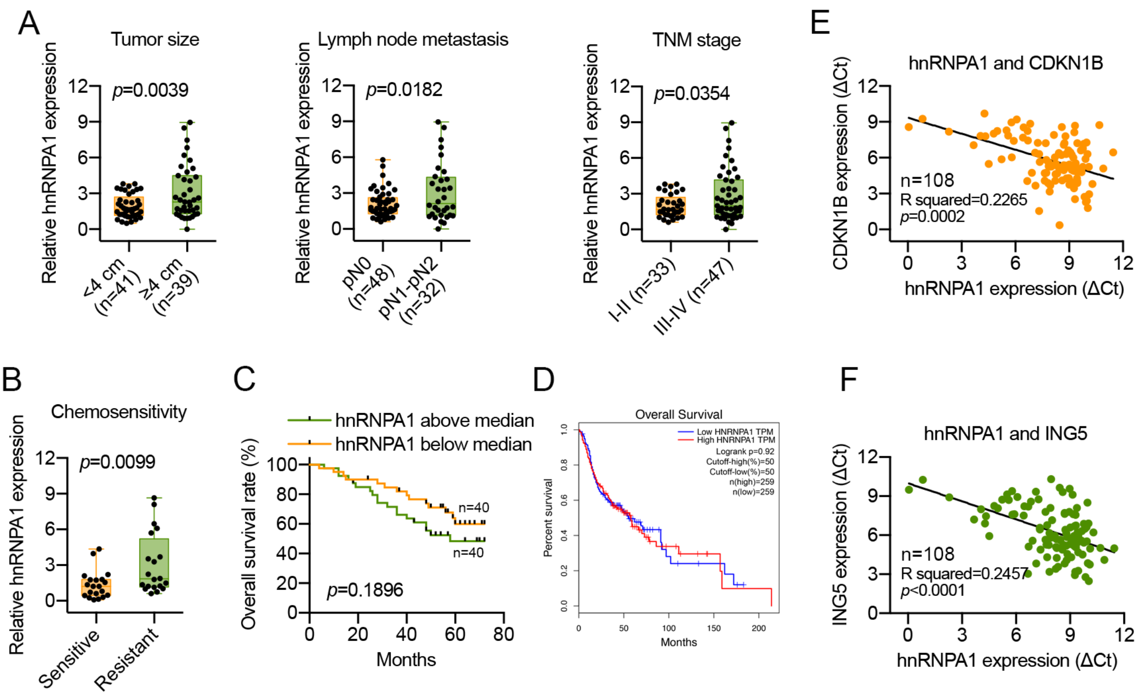

Supplementary Figure S10

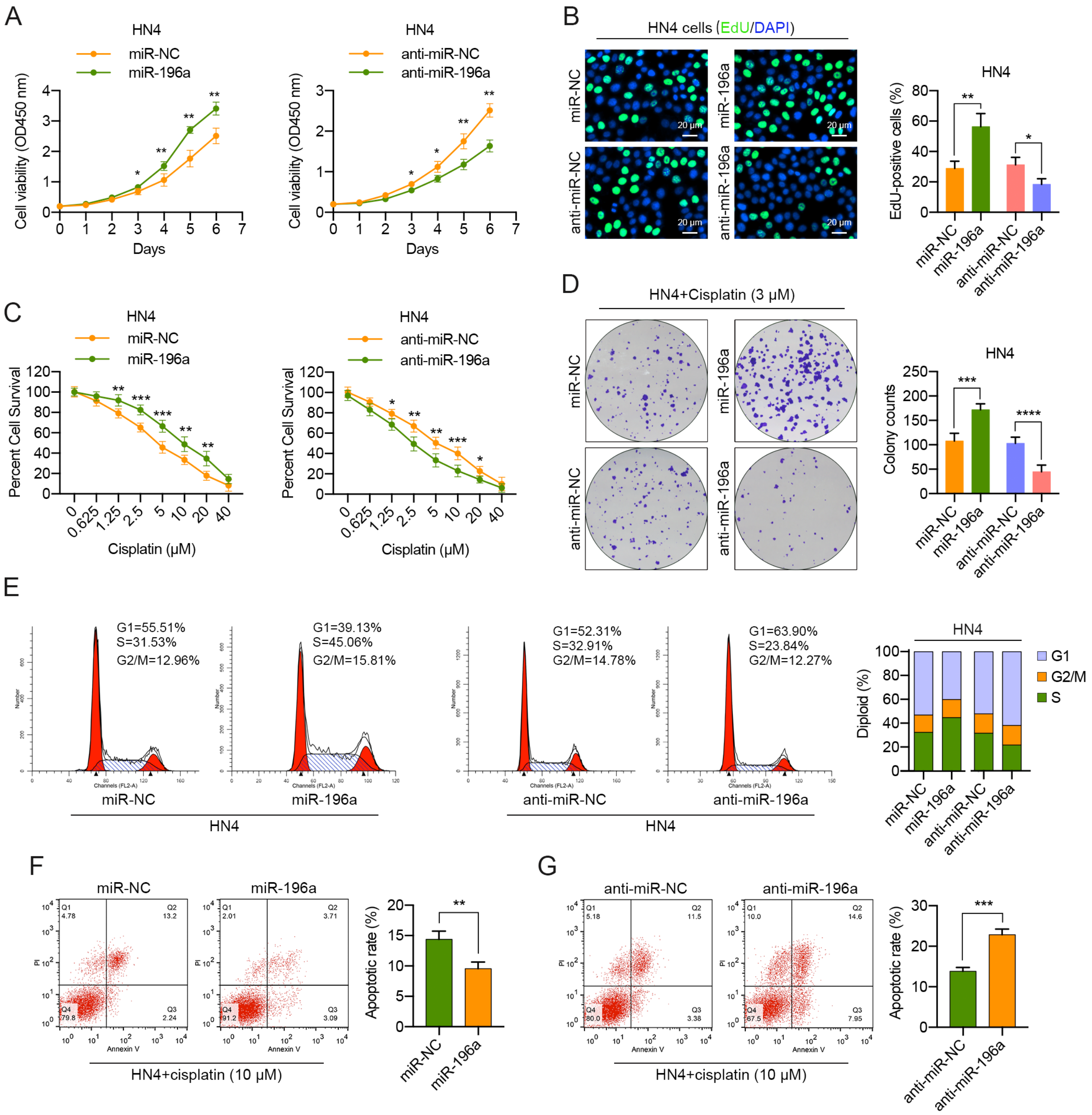

Supplementary Figure S11

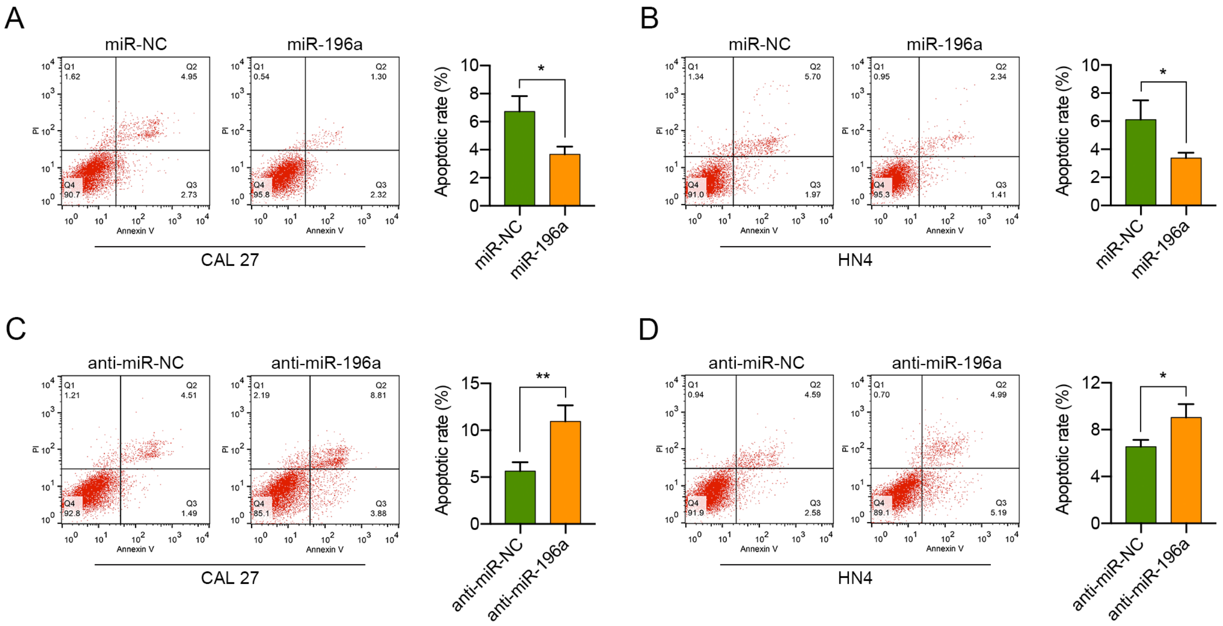

Supplementary Figure S12

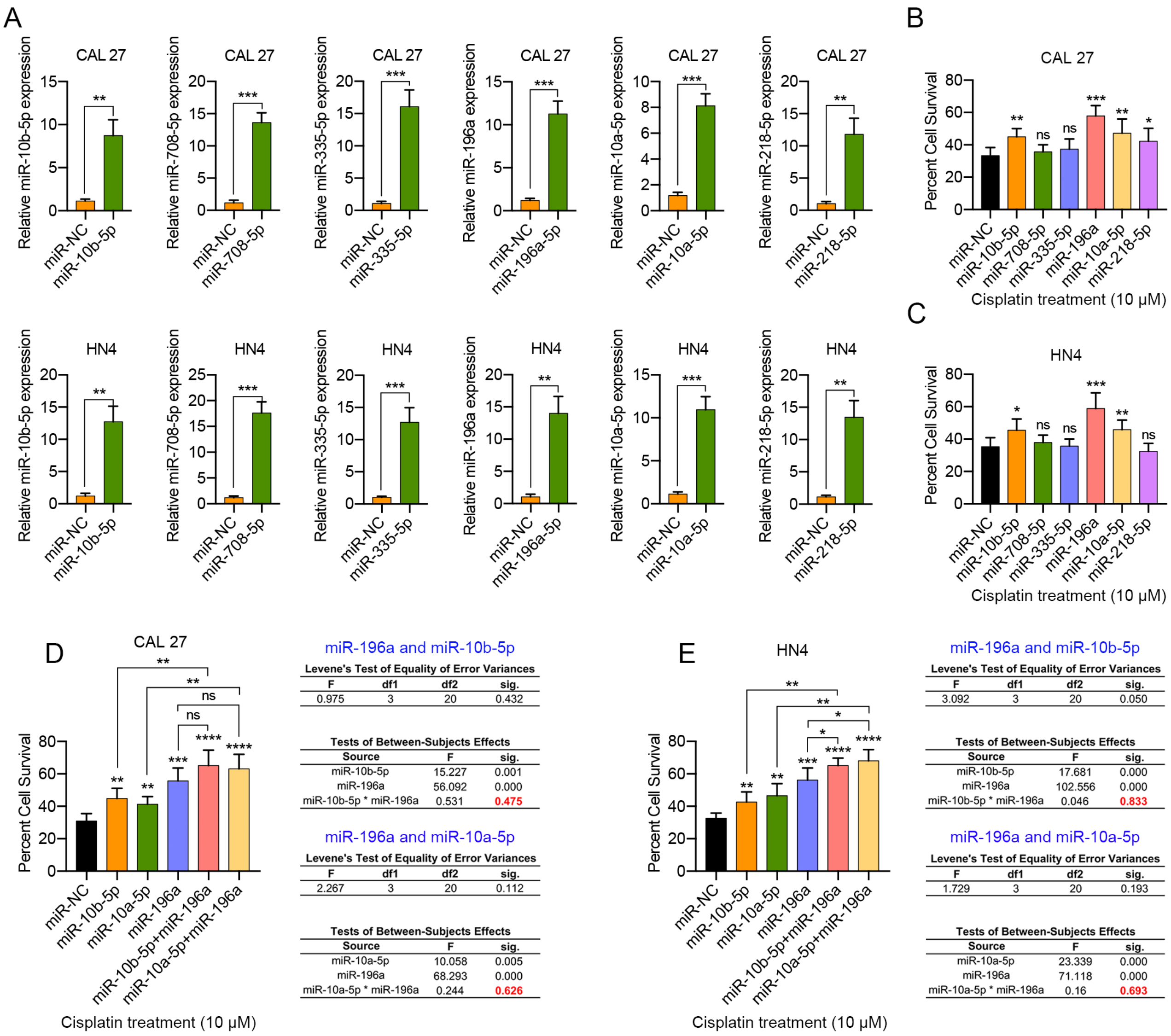

D (CAL 27)

miR-196a and miR-10b-5p

Levene's Test of Equality of Error Variances

F	df1	df2	sig.
0.975	3	20	0.432

Tests of Between-Subjects Effects

Source	F	sig.
miR-10b-5p	15.227	0.001
miR-196a	56.092	0.000
miR-10b-5p * miR-196a	0.531	0.475

miR-196a and miR-10a-5p

Levene's Test of Equality of Error Variances

F	df1	df2	sig.
2.267	3	20	0.112

Tests of Between-Subjects Effects

Source	F	sig.
miR-10a-5p	10.058	0.005
miR-196a	68.293	0.000
miR-10a-5p * miR-196a	0.244	0.626

E (HN4)

miR-196a and miR-10b-5p

Levene's Test of Equality of Error Variances

F	df1	df2	sig.
3.092	3	20	0.050

Tests of Between-Subjects Effects

Source	F	sig.
miR-10b-5p	17.681	0.000
miR-196a	102.556	0.000
miR-10b-5p * miR-196a	0.046	0.833

miR-196a and miR-10a-5p

Levene's Test of Equality of Error Variances

F	df1	df2	sig.
1.729	3	20	0.193

Tests of Between-Subjects Effects

Source	F	sig.
miR-10a-5p	23.339	0.000
miR-196a	71.118	0.000
miR-10a-5p * miR-196a	0.16	0.693

Supplementary Figure S13

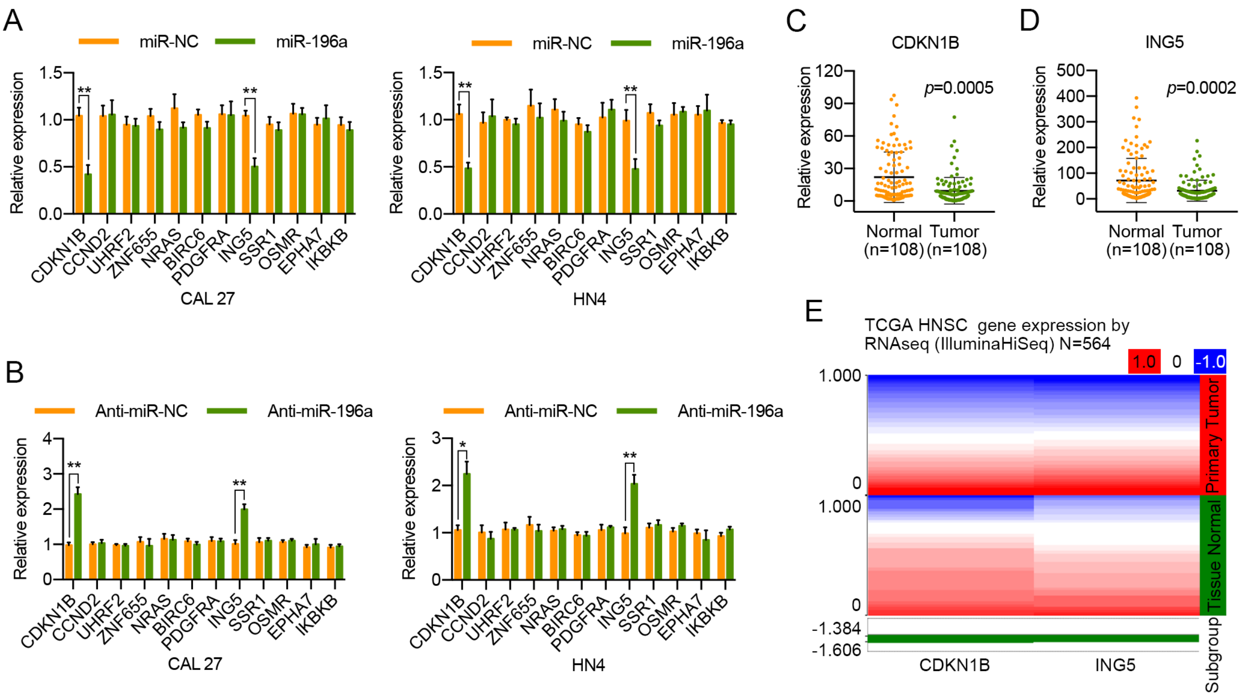

Supplementary Figure S14

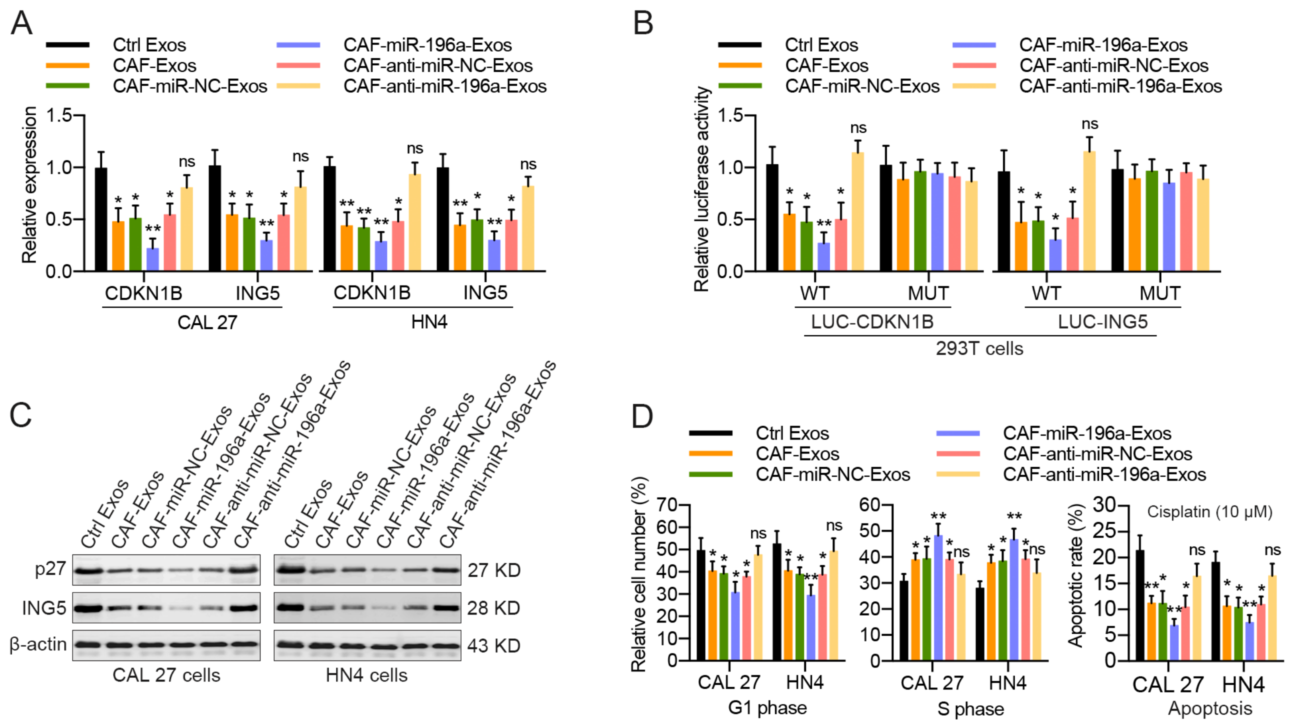

Supplementary Figure S15

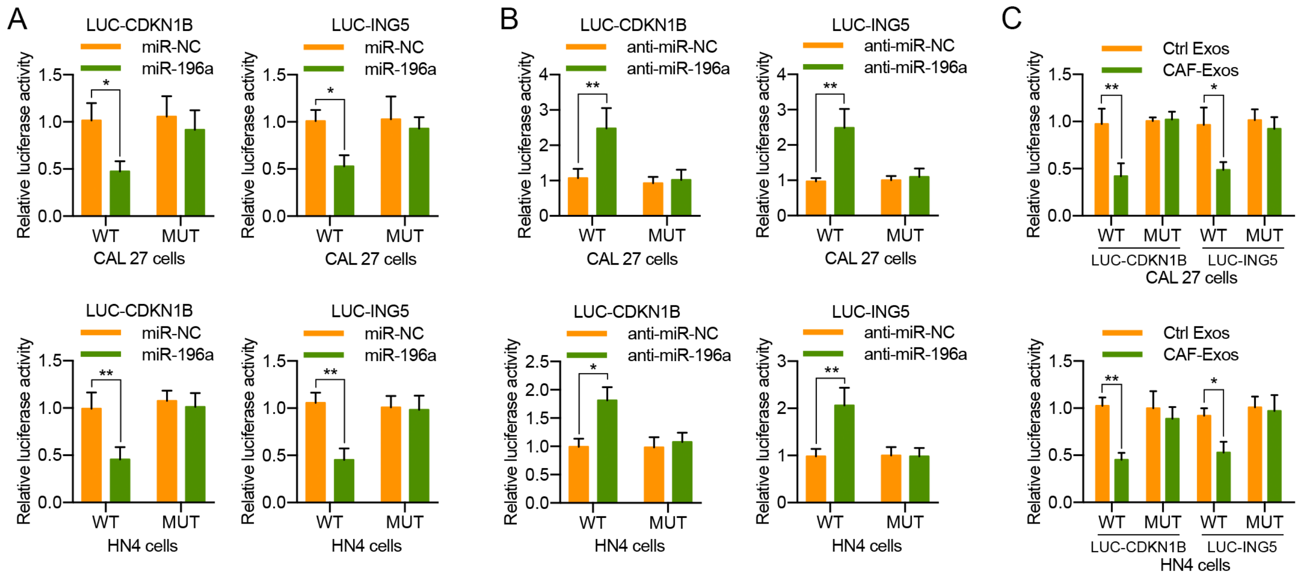

Supplementary Figure S16

A

Evolutionally conserved information (obtained from TargetScan database)

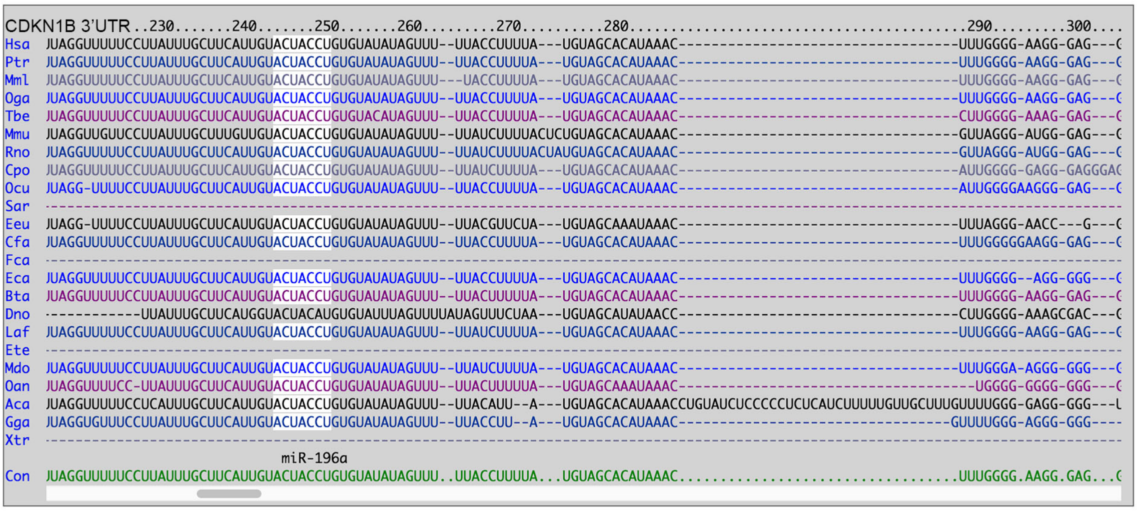

Conserved sites for miR-196a in CDKN1B 3' UTR broadly conserved among vertebrates

B

Evolutionally conserved information (obtained from TargetScan database)

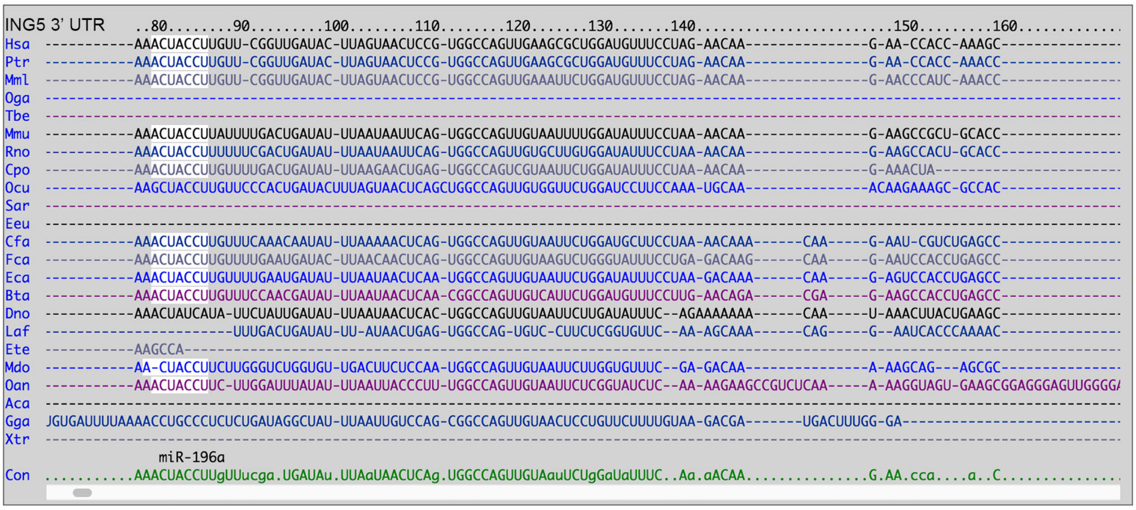

Conserved sites for miR-196a in ING5 3' UTR broadly conserved among vertebrates

Supplementary Figure S17

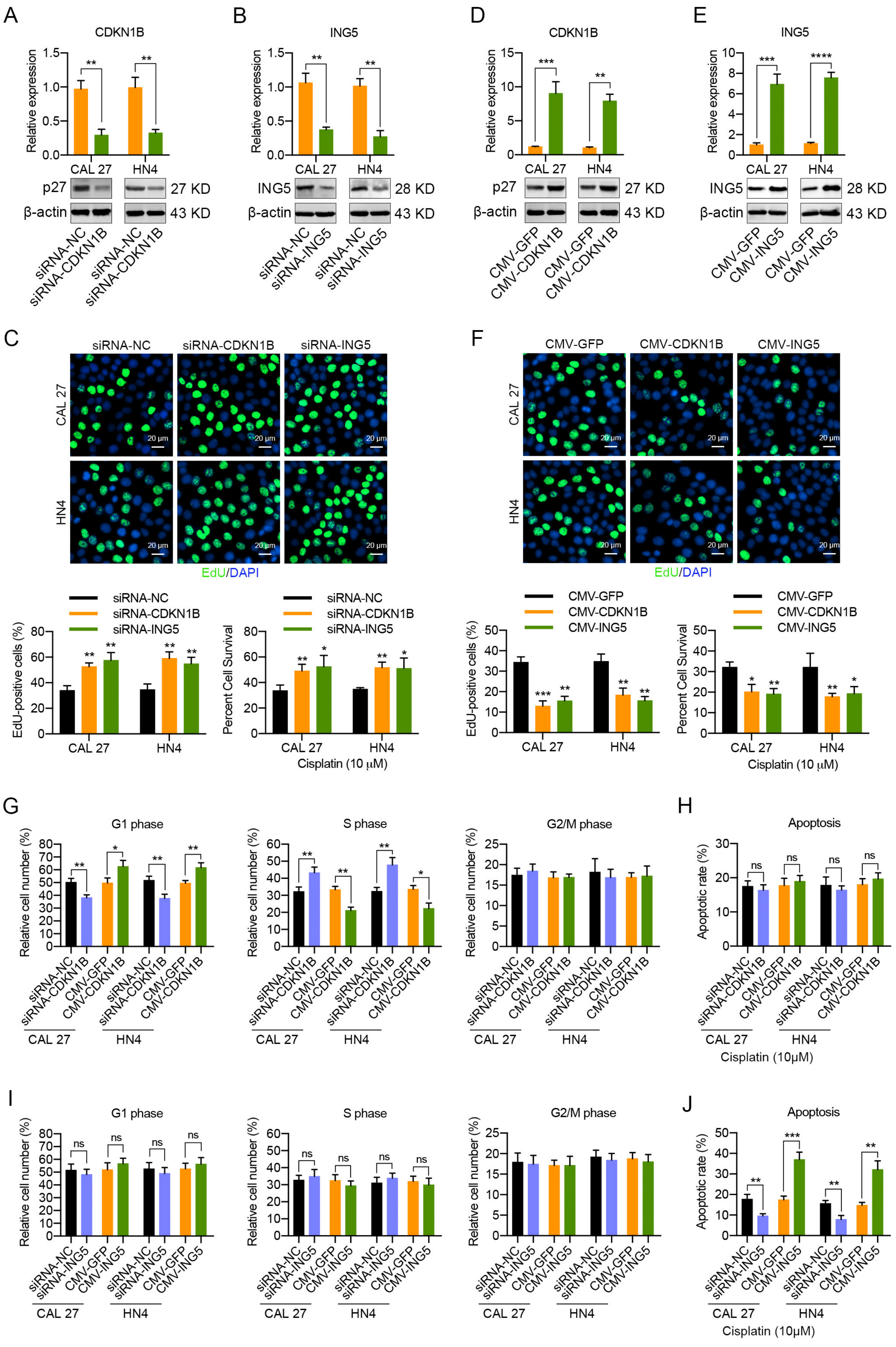

Supplementary Figure S18

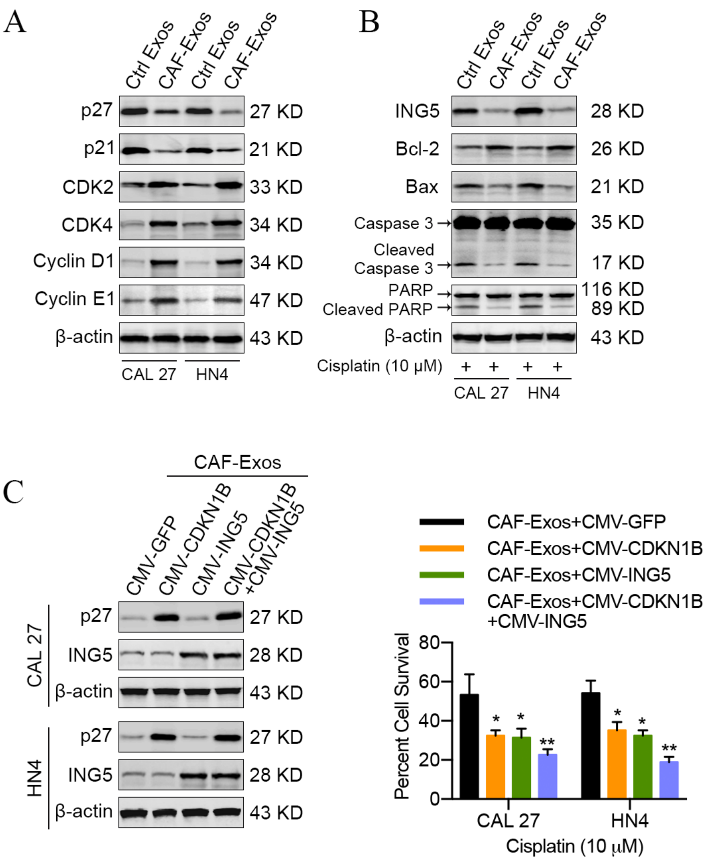

Supplementary Figure S19

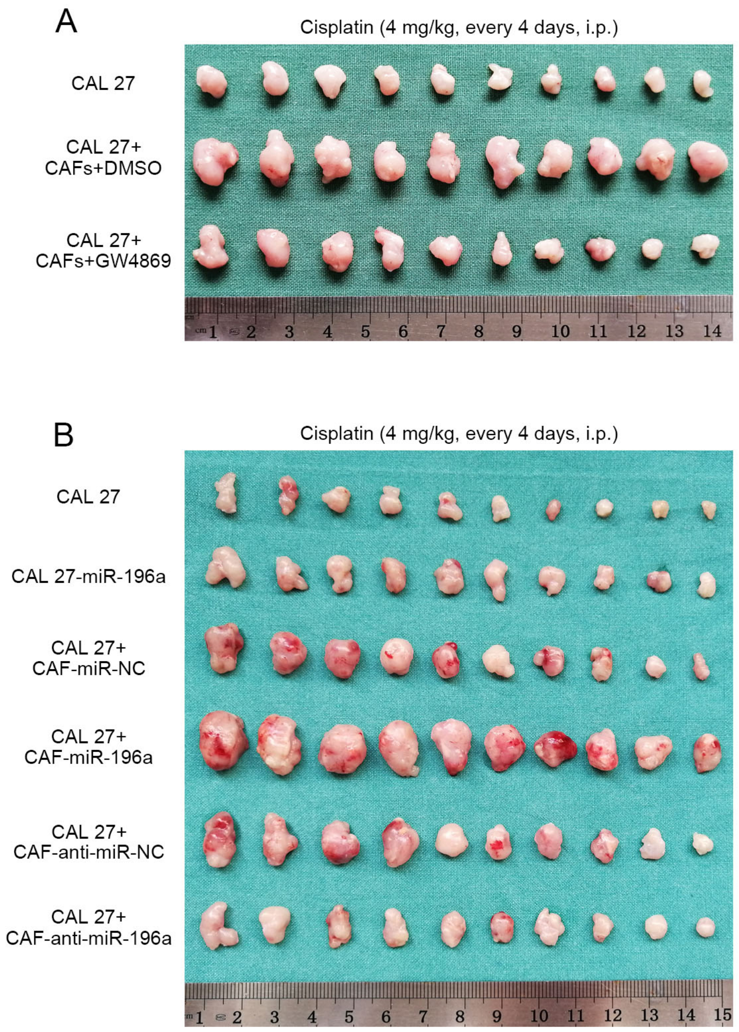

Supplementary Figure S20

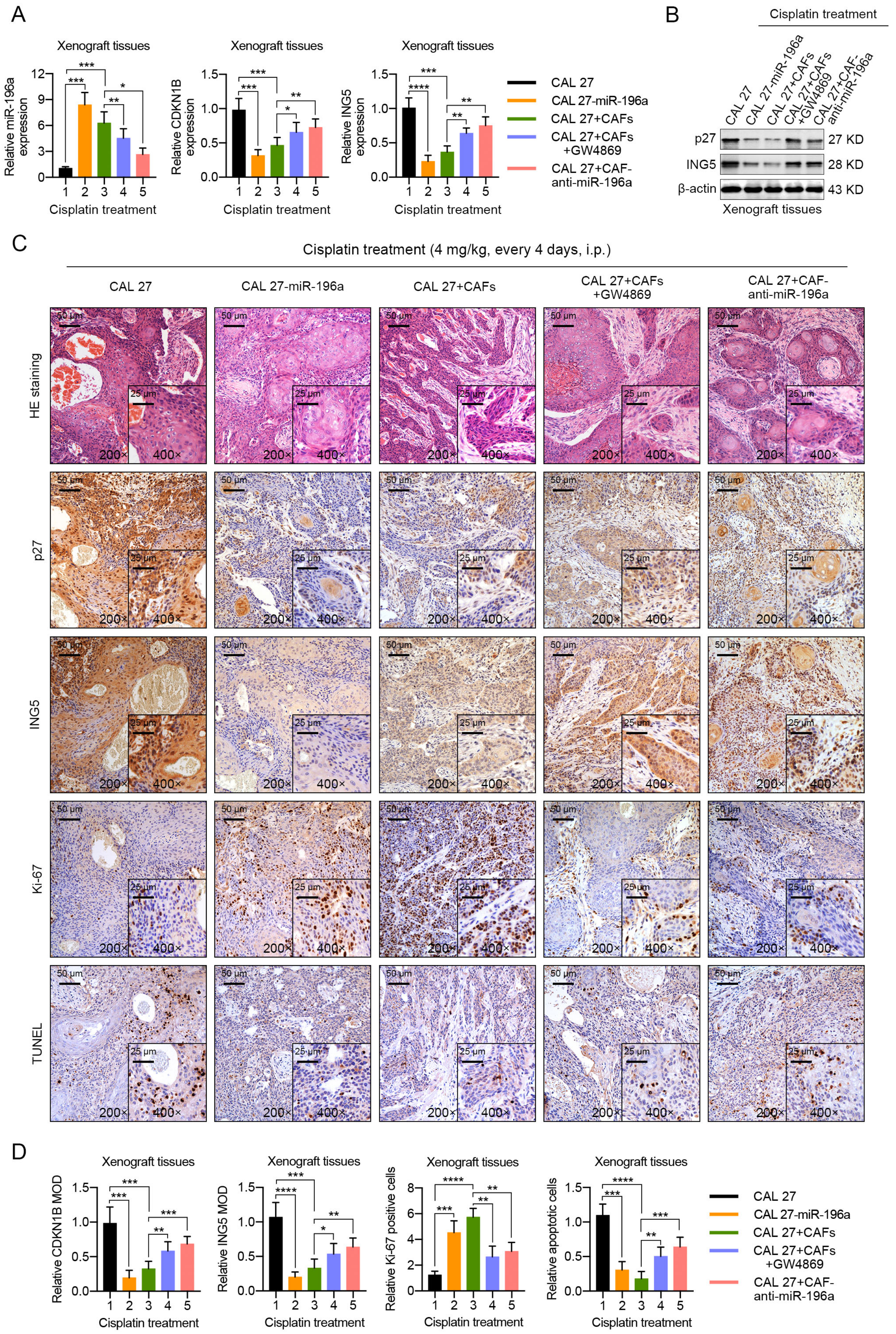

Supplementary Figure S21

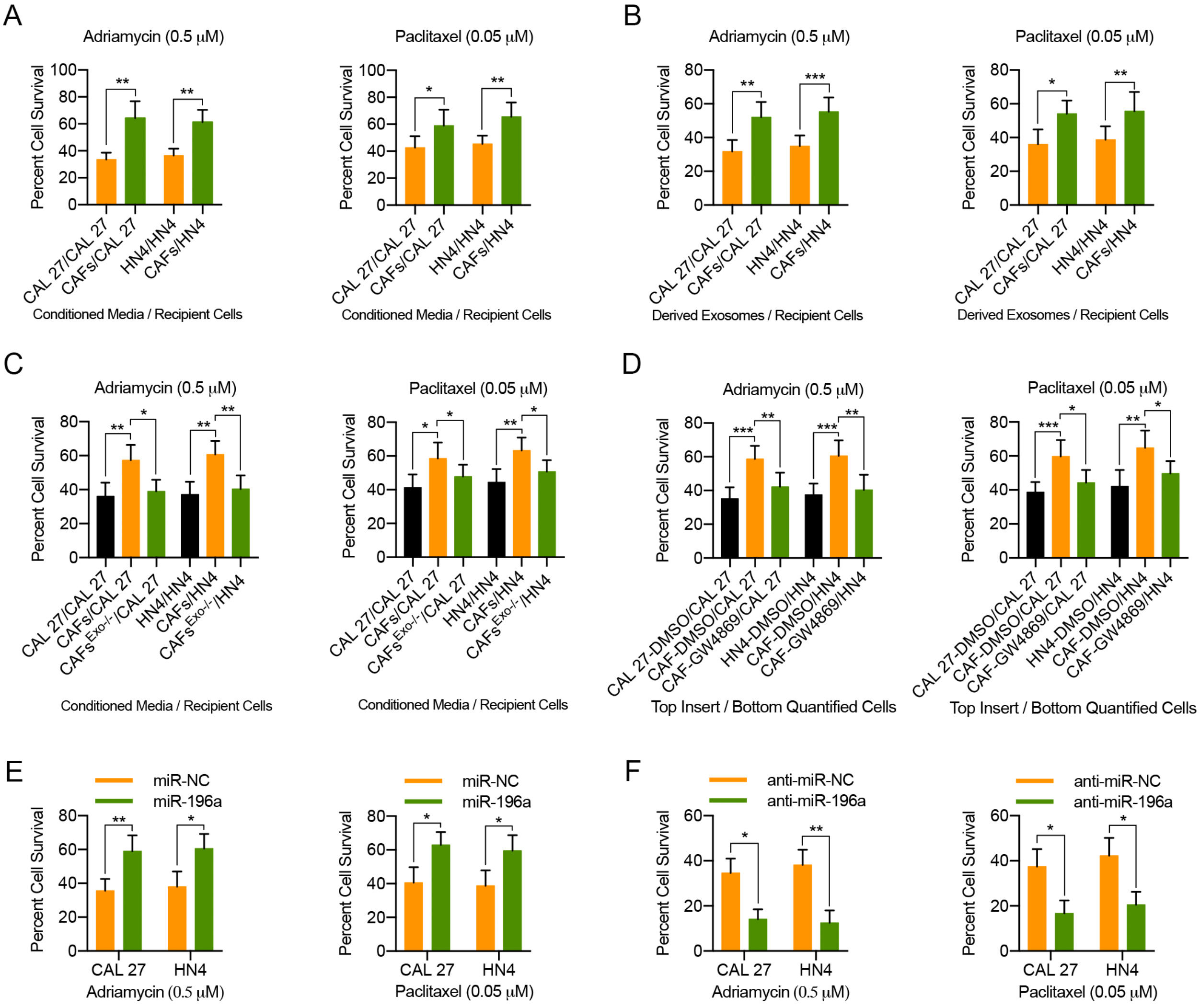

Supplementary Figure S22

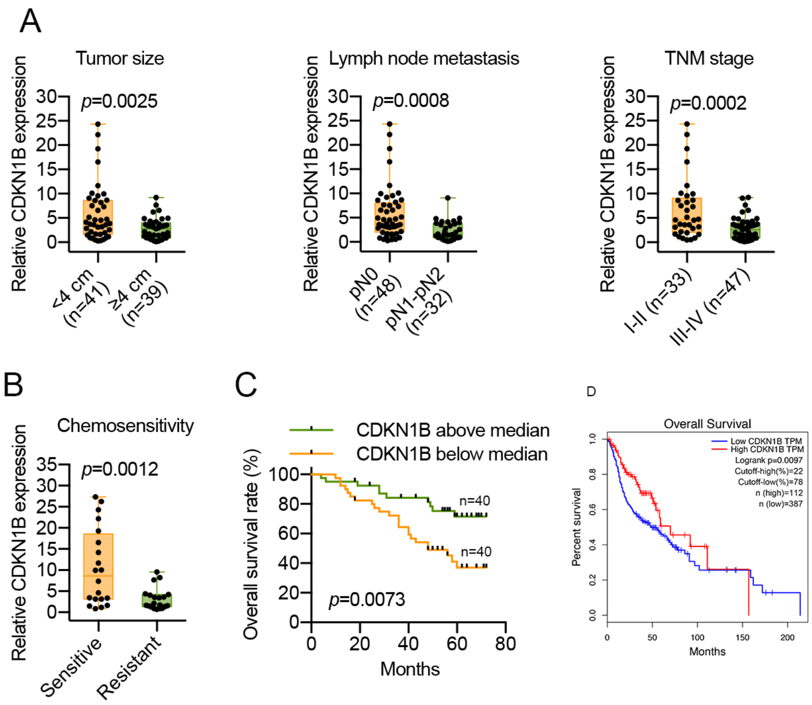

Supplementary Figure S23

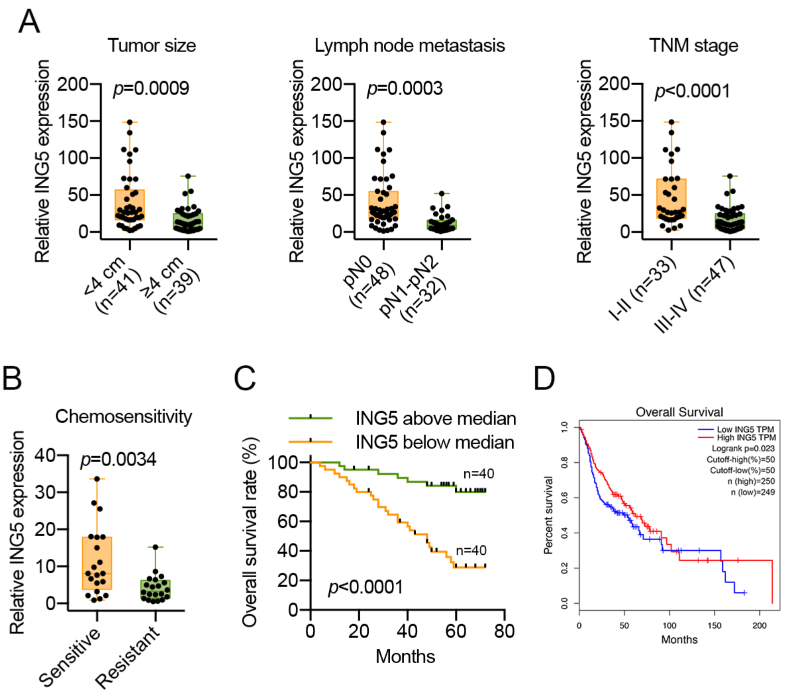

Supplementary Figure S24

Supplementary Figure legends

Figure S1. CAFs are insensitive to cisplatin treatment.

a. Immunohistochemical analysis of α-SMA protein expression in HNC tissues and matched adjacent normal tissues (Scale bar: 50 μm), and morphological images of NFs and CAFs under an inverted microscopy. (Scale bar: 20 μm). MOD, mean optical density. **b.** The IC50 of cisplatin in NFs, CAFs and primary cancer cells derived from HNC tissues (n = 12). **c.** Cisplatin IC50 of CAL 27, SCC-25, HN4 and HN4-res cells determined by constructing a dose-response curve. **d.** The mRNA expression levels of MRP2, ATP7B, CTR1, XIAP, ERCC1, ERCC4, GSTK1, Bcl-2 were detected in NFs, CAFs, HNC cells (CAL 27, SCC-25 and HN4) and HN4-res cells. **e.** Real-time PCR analysis of the expression levels of ERCC1 and ERCC4 in CAFs at 48 h after transfection with gene-specific siRNA. **f.** Western blot showing ERCC1 and ERCC4 protein levels in CAFs at 48 h after transfection with siRNAs specific for ERCC1 or ERCC4. **g.** MTT assay showing the cisplatin response of CAFs at 48 h after transfection, as indicated. (ns, no significant difference; *$p<0.05$; ***p*<0.01; ****p*<0.001; *****p*<0.0001)

Figure S2. CAFs accelerate HNC cell proliferation and cisplatin resistance.

a. CAL 27 and HN4 cells were grown in control CM, NF-CM or CAF-CM for 6 days, and cell viability was examined. MTT assays were carried out to detect the cisplatin response of these cells. **b.** The cell viability of CAL 27 and HN4 cells was assessed after incubating with control CM or HN4-res-CM for 6 days. The refractoriness of

these cells to cisplatin was evaluated by MTT assays. **c.** HNC cells were treated with exosomes (25 μg/mL) from HNC cells, cisplatin-treated (10 μM) NFs or cisplatin-treated (10 μM) CAFs for 6 days, and the cell viability was detected. The cisplatin response of CAL 27 and HN4 cells was obtained using MTT assays. **d.** The cell viability of HNC cells was examined after incubating with exosomes (25 μg/mL) from HNC cells, HN4-res cells or cisplatin-treated (10 μM) HN4-res cells for 6 days. The survival percentage of HNC cells upon cisplatin treatment was measured by MTT assays. (NT: without cisplatin treatment; CT: cisplatin treatment; ns, no significant difference; **p*<0.05; ***p*<0.01; ****p*<0.001; *****p*<0.0001)

Figure S3. GW4869 inhibits the production of exosomes derived from CAFs.

a. NanoSight particle-tracking analysis of size distribution and the number of exosomes from CAFs or GW4869-treated CAFs (20 μM). **b.** NanoSight particle-tracking analysis of the number of exosomes from CAFs or GW4869-treated CAFs (20 μM). **c.** Exosomal protein concentration in CM from CAFs or GW4869-treated CAFs (20 μM). **d, e.** CAL 27 and HN4 cells were co-cultured with CAFs expressing CD63-GFP fusion protein for 48 h. Fluorescence microscopy was used to detect green fluorescent signals in HNC cells (Scale bar: 10 μm). (***p*<0.01; ****p*<0.001)

Figure S4. Identification of CAF-derived exosomes.

a. The exosome fractions and cell lysates of CAFs (CAF1 and CAF2) were analyzed

by immunoblotting with antibodies against exosomal proteins (Alix, HSP90, HSP70, CD63, CD9 and Rab5) and cellular proteins GRP94. Equal amounts of protein from cell lysates or exosomes were used for each assay. **b.** NanoSight particle-tracking analysis of the size distributions and number of exosomes from CAFs (CAF1, CAF2 and CAF3). **c.** Representative electron microscopy images of exosomes secreted by CAFs (CAF1, CAF2 and CAF3; Scale bar, 50 nm).

Figure S5. Cisplatin promotes the biogenesis, transport and release of exosomes in CAFs.

a. Molecules shown to affect exosome biogenesis, transport or release. b. The expression levels of exosome biogenesis- or release-associated genes (HRS, TSG101, STAM1, VPS4B, CD9, CD63, nSMase2, PLD2, RAB11A, RAB35, RAB2B, RAB5A, RAB9A, RAB27A, RAB27B, RAB7, YKT6, PKM2 and ATG7) were examined in CAFs at 24 h after the treatment with or without cisplatin (10 μM) using real-time PCR analysis. (NT: without cisplatin treatment; CT: cisplatin treatment; *p<0.05; **p<0.01; ***p<0.001)

Figure S6. Enrichment of miR-196a in CAF-derived exosomes in HNC.

a. To evaluate the accuracy of miRNA array, 6 up-regulated miRNAs and 6 down-regulated miRNAs were randomly selected for further identification. The expression level of these miRNAs was detected in the exosomes from 10 pairs of NFs and CAFs using real-time PCR. **b.** The expression level of 22 up-regulated miRNAs

(Fold change > 2) in exosomes from cisplatin-treated CAFs compared with those from untreated CAFs. **c, d.** The expression of miR-10b-5p, miR-708-5p, miR-335-5p, miR-196a-5p, miR-10a-5p and miR-218-5p in CAL 27 and HN4 cells was measured using real-time PCR at 24 h after incubating with exosomes (25 μg/mL) from cisplatin-treated CAFs. **e.** miR-196a expression level in 108 pairs of HNC samples and adjacent normal tissues. **f.** miR-196a expression level in HNCs according to the Cancer Genome Atlas (TCGA; UCSC Cancer Genomics Browser, https://genome-cancer.soe.ucsc.edu/). Red indicates the up-regulated cases while the blue indicates the down-regulated cases. **g.** miR-196a expression level was determined in NFs, CAFs and primary cancer cells (derived from 6 HNC patients) using real-time PCR. (*p<0.05; **p<0.01; ***p<0.001; ****p<0.0001)

Figure S7. Cisplatin increases the production of exosomal miR-196a in CAFs.

a. Real-time PCR analysis of miR-196a expression in CAFs (n = 10) treated with or without cisplatin (10 μM). **b.** Western blot analysis of p38, p-p38, ERK, p-ERK, JNK and p-JNK protein levels in CAFs treated with or without cisplatin (10 μM) for 2 h. **c.** CAFs were pre-treated for 2 h with 30 μM p38 inhibitor (SB203580, Selleck) or for 1 h with 40 μM ERK inhibitor (PD98059, Selleck) followed by treatment with 10 μM cisplatin for 2 h. The protein levels of p38, p-p38, ERK and p-ERK were detected by western blotting, and the miR-196a expression level was measured using real-time PCR. **d.** Putative transcription factors that could bind to the miR-196a promoter were predicted using the PROMO Database and were subjected to KEGG_PATHWAY

analysis. **e.** Diagram showing the miR-196a 5' flanking DNA. A 2,000 bp miR-196a promoter segment was divided into 4 compartments and primers were designed for each compartment. **f.** CAFs were incubated with or without cisplatin (10 μM) for 2 h in advance. ChIP assays using anti-NF-1, anti-p53, anti-c-Myc or anti-IgG antibodies were performed to determine the affinity of these transcription factors for the miR-196a promoter in CAFs. **g.** CAFs were transiently transfected with c-Myc-specific siRNAs for 24 h followed by treatment with or without cisplatin (10 μM) for 48 h. The protein levels of c-Myc were detected by western blotting, and miR-196a expression was measured using real-time PCR. **h.** CAFs were pre-treated with 50 μM 10058-F4 (a c-Myc inhibitor purchased from MCE) for 24 h followed by treatment with 10 μM cisplatin for 48 h. The protein levels of c-Myc were detected by western blotting, and the miR-196a expression was measured using real-time PCR. **i.** CAFs were treated with or without 10 μM cisplatin for 48 h, and the cells or exosomes were collected from CM, respectively. Western blot analysis was performed to detect hnRNPA1 protein levels in CAFs lysates or the hnRNPA1 and Alix protein levels in exosomal lysates, and the hnRNPA1 mRNA level was measured using real-time PCR. (NT: without cisplatin treatment; CT: cisplatin treatment; ns, no significant difference; ***p*<0.01; ****p*<0.001)

Figure S8. The expression of miR-196a in CAFs and HNC cells after miR-196a knockdown or overexpression.

a. Real-time PCR analysis of miR-196a expression level in CAFs, CAL-27 and HN4

cells at 48 h after transfection with miR-196a. **b.** The results of real-time PCR showing miR-196a expression level in CAFs and HNC cells at 48 h after transfection with anti-miR-196a. (**p<0.01; ***p<0.001; ****p<0.0001)

Figure S9. hnRNPA1 mediates the transfer of miR-196a from CAFs to HNC cells *in vivo*.

a. CAL 27 and HN4 cells were transfected with an hnRNPA1-expressing or hnRNPA1-silencing plasmid for 48 h; the expression of hnRNPA1 was detected using western blotting and real-time PCR. **b.** Nude mice were subcutaneously xenografted with a mixture of CAL 27 cells plus CAFs transfected with hnRNPA1, sh-NC or sh-hnRNPA1. The tumor weights are shown. **c.** Immunohistochemical staining was performed to analyze hnRNPA1 expression in xenograft tissues, especially in tumor stroma. MOD, mean optical density (Scale bar: 50 μm). d. The distribution of miR-196a in xenograft tumors was detected using FISH assays and miR-196a expression in tumor cells was assessed. Tissues were stained with α-SMA to detect CAFs and cells were stained with DAPI to detect nuclei (Scale bar: 20 μm). (**p<0.01; ***p<0.001)

Figure 10. Upregulation of hnRNPA1 is associated with malignant transformation in HNC.

a. Upregulated hnRNPA1 levels were correlated with large tumor size, lymph node metastasis and advanced tumor stage in HNC tissues. **b.** Real-time PCR analysis

showing hnRNPA1 expression in HNC tissues from chemosensitive patients (n = 20) and chemoresistant patients (n = 20). **c.** Kaplan-Meier analyses of overall survival. There was no significant difference between the overall survival rates of high hnRNPA1 expression group and low hnRNPA1 expression group. **d**. Kaplan-Meier analysis of overall survival of 499 HNC patients in the high and low hnRNPA1 groups using Gene Expression profiling Interactive Analysis (GEPIA, http://gepia.cancer-pku.cn/index.html). **e, f.** Correlation analysis was performed between miR-196a downstream targets (CDKN1B and ING5) and hnRNPA1 (n = 108).

Figure S11. miR-196a regulates HNC cell proliferation and survival by promoting G1/S transition and apoptosis resistance.

a. Cell proliferation assays with HN4 cells at 48 h after transfection with miR-196a or anti-miR-196a. **b.** Representative micrographs and quantification of EdU-incorporating cells at 48 h after transfection with miR-196a or anti-miR-196a. **c.** MTT assay of HN4 cells transfected with miR-196a or anti-miR-196a for 48 h, followed by cisplatin treatment at the indicated concentration for 72 h. **d.** Plate colony formation assay of HN4 cells transfected with miR-196a or anti-miR-196a with cisplatin treatment (3 μM). **e.** The cell cycle distribution was analyzed by a flow cytometer in HN4 cells transfected with miR-196a or anti-miR-196a at 48 h after transfection. **f, g.** Flow cytometric analysis of cisplatin-induced (10 μM) apoptosis in HN4 cells transfected with miR-196a or anti-miR-196a at 48 h after transfection.

(*$p<0.05$; **$p<0.01$; ***$p<0.001$)

Figure S12. miR-196a regulates HNC cell apoptosis.

a, b. CAL 27 and HN4 cells were transfected with or without miR-196a for 48 h, and apoptotic rates of these cells were detected using a flow cytometer. **c, d.** Flow cytometric analysis of the apoptosis of HNC cells at 48 h after transfection with or without anti-miR-196a. (*$p<0.05$; **$p<0.01$)

Figure S13. miR-196a exhibits no synergistic effect with other upregulated miRNAs on cisplatin resistance.

a. Real-time PCR analysis of miR-10b-5p, miR-708-5p, miR-335-5p, miR-196a, miR-10a-5p and miR-218-5p expression levels in CAL 27 and HN4 cells at 48 h after transfection with or without miR-10b-5p, miR-708-5p, miR-335-5p, miR-196a, miR-10a-5p or miR-218-5p mimics, respectively. **b, c.** CAL 27 and HN4 cells were transfected with miR-NC, miR-10b-5p, miR-708-5p, miR-335-5p, miR-196a, miR-10a-5p or miR-218-5p mimics for 48 h, and then MTT assays were performed to detect the cisplatin response of these cells. **d, e.** CAL 27 and HN4 cells were transfected with miR-NC, miR-10b-5p, miR-10a-5p, miR-196a, miR-10b-5p plus miR-196a or miR-10a-5p plus miR-196a for 48 h, and then, MTT assays were performed to detect the cisplatin response of these cells. A general linear model was constructed to assess the synergistic effect between miR-10b-5p and miR-196a or between miR-10a-5p and miR-196a. (ns, no significant difference; *$p<0.05$; **$p<0.01$;

***p<0.001; ****p<0.0001)

Figure S14. CDKN1B and ING5 are negatively regulated by miR-196a.

a, b. After a 48 h transfection with miR-196a or anti-miR-196a in CAL 27 and HN4 cells, the expression level of 12 potential targets for miR-196a was analyzed using real-time PCR. **c, d.** The mRNA level of CDKN1B and ING5 in 108 pairs of HNC samples and adjacent normal tissues. **e.** The expression level of CDKN1B and ING5 in HNCs according to the Cancer Genome Atlas (TCGA; UCSC Cancer Genomics Browser, https://genome-cancer.soe.ucsc.edu/). Red indicates the up-regulated cases while the blue indicates the down-regulated cases. (*p<0.05; **p<0.01)

Figure S15. CAF-derived exosomal miR-196a regulates HNC cell cycle and apoptosis by downregulation of CDKN1B and ING5.

Exosomes were purified from the CM of HNC cells (Ctrl) or CAFs transfected with miR-196a or anti-miR-196a. Further experiments were carried out with CAL 27 and HN4 cells at 48 h after incubating with exosomes (25 μg/mL), as indicated. **a.** The mRNA level of CDKN1B and ING5 in HNC cells treated as indicated. **b.** Effects of the indicated exosomes on CDKN1B or ING5 reporter luciferase activity in 293T cells. **c.** Western blot analysis showing the protein level of p27 and ING5 in CAL 27 and HN4 cells after exosome treatment, as indicated. **d.** The results of cell cycle and cisplatin-induced apoptosis in HNC cells after incubating with indicated exosomes. (ns, no significant difference; *p<0.05; **p<0.01)

Figure S16. CDKN1B and ING5 are direct targets of exosomal miR-196a in HNC.

a. Relative CDKN1B or ING5 reporter activities in HNC cells (CAL 27 and HN4) co-transfected with miR-196a and luciferase reporters. **b.** The effects of anti-miR-196a on CDKN1B or ING5 reporter luciferase activity in HNC cells (CAL 27 and HN4). **c.** The effects of CAF-derived exosomes (25 μg/mL) on CDKN1B or ING5 reporter luciferase activity in HNC cells (CAL 27 and HN4). (*p<0.05; **p<0.01)

Figure S17. Conserved sites for miR-196a in CDKN1B and ING5 3'UTR.

a, b. Evolutionally conserved information for miR-196a in CDKN1B and ING5 3'UTR was obtained from the TargetScan Human database (http://www.targetscan.org/).

Figure S18. Effects of CDKN1B and ING5 genes on HNC cell proliferation and survival.

a, b. The results of western blotting and real-time PCR showing the knockdown of CDKN1B or ING5 in CAL 27 and HN4 cells by transfection of specific siRNAs. **c.** Silencing of CDKN1B or ING5 promoted the growth (EdU-labeling assay) and cisplatin resistance (MTT assay) of CAL 27 and HN4 cells. **d, e.** CAL 27 and HN4 cells were transfected with CDKN1B- or ING5-expressing plasmid for 48 h; the expression of CDKN1B and ING5 was detected using western blotting and real-time

PCR. **f.** Overexpression of CDKN1B or ING5 suppressed the proliferation (EdU-labeling assay) and cisplatin resistance (MTT assay) of CAL 27 and HN4 cells. **g-j.** Dysregulation of CDKN1B in HNC cells mainly affected the G1/S cell cycle transition, whereas ING5 predominantly modulated the cell apoptosis of HNC cells. (ns, no significant difference; **p*<0.05; ***p*<0.01; ****p*<0.001; *****p*<0.0001)

Figure S19. CAF-derived exosomes accelerate cisplatin resistance in HNC cells via CDKN1B and ING5 downregulation.

a. Western blot showing p27, p21, CDK2, CDK4, Cyclin D1 and Cyclin E1 protein levels in CAL 27 and HN4 cells at 48 h after incubating with or without CAF-derived exosomes (25 μg/mL). **b.** Western blot showing ING5, Bcl-2, Bax, full-length Caspase 3, cleaved Caspase 3, full-length PARP and cleaved PARP protein levels in CAL 27 and HN4 cells at 48 h after treatment with CAF-derived exosomes (25 μg/mL). **c.** CAL 27 and HN4 cells, transfected with an exogenous expression vector (CDKN1B or ING5), were treated with CAF-derived exosomes (25 μg/mL) for 48 h. Left: western blot showing p27 and ING5 protein levels in CAL 27 and HN4 cells, as indicated. Right: cisplatin response of CAL 27 and HN4 cells after treatment as indicated. (**p*<0.05; ***p*<0.01)

Figure S20. CAF-derived exosomal miR-196a enhances cisplatin resistance in HNC cells *in vivo*.

a. CAL 27 cells with or without CAFs were used to establish a xenograft mice model,

as indicated. The mice were intraperitoneally injected with cisplatin (4 mg/kg, every 4 days) with or without GW4869 (2 mg/kg, every 2 days). The tumor sizes are shown. **b.** CAL 27 cells were stably transfected with or without miR-196a, and CAFs were stably transfected with miR-196a or anti-miR-196a. Nude mice were subcutaneously xenografted with pre-transfected CAL 27 cells, CAFs or a mixture of CAL 27 cells plus pre-transfected CAFs, as indicated. The mice were intraperitoneally injected with cisplatin (4 mg/kg, every 4 days) for 5 times. The tumor sizes are shown.

Figure S21. CAF-derived exosomal miR-196a enhances cisplatin resistance of HNC *in vivo* via CDKN1B and ING5 downregulation.

Nude mice were subcutaneously xenografted with pre-transfected CAL 27 cells or CAFs as indicated. The mice were intraperitoneally injected with cisplatin (4 mg/kg, every 4 days) with or without GW4869 (2 mg/kg, every 2 days). The transplanted tumors were removed for further analysis after the mice were sacrificed. **a.** Real-time PCR analysis showing the expression level of miR-196a, CDKN1B and ING5 in tumor tissues from different groups, as indicated. **b.** Western blot analysis exhibiting p27 and ING5 protein expressions in tumor tissues from indicated groups. **c, d.** H&E staining of tumors harvested from different groups; Immunohistochemical staining was performed to analyze p27, ING5 and Ki-67 expressions in xenograft tissues, and the apoptotic cells in the tumor tissues were assessed using TUNEL assay. MOD, mean optical density (Scale bar, 200×: 50 μm; 400×: 25 μm). (*p<0.05; **p<0.01; ***p<0.001; ****p<0.0001)

Figure S22. CAF-derived exosomal miR-196a endows HNC cells with resistance to adriamycin and paclitaxel.

a. CAL 27 and HN4 cells were grown in CAF-CM or control CM for 6 days, and MTT assays were performed to detect the resistance of these cells to adriamycin or paclitaxel. **b.** CAL 27 and HN4 cells were incubated with exosomes (25 μg/mL) from HNC cells or CAFs for 6 days, and MTT assays were carried out to detect the resistance of these cells to adriamycin or paclitaxel. **c.** CAL 27 and HN4 cells were grown in control CM, CAF-CM, or exosome-depleted CAF-CM for 6 days, and MTT assays were performed to detect the tolerance of these cells to adriamycin or paclitaxel. **d.** HNC cells were co-cultured with DMSO-treated HNC cells, DMSO-treated CAFs or GW4869-treated CAFs for 6 days, and the survival percentage of co-cultured HNC cells was measure by MTT assays upon adriamycin or paclitaxel treatment. **e.** MTT assays of HNC cells transfected with or without miR-196a for 48 h followed by adriamycin or paclitaxel treatment, at indicated concentration for 72 h. **f.** HNC cells transfected with or without anti-miR-196a for 48 h, and the survival percentage of these cells were evaluated by MTT assays upon adriamycin or paclitaxel treatment. (*p<0.05; **p<0.01; ***p<0.001)

Figure S23. Downregulated CDKN1B levels were associated with malignant transformation and lower overall survival in HNC.

a. Downregulated CDKN1B levels were correlated with large tumor size, lymph node metastasis and advanced tumor stage in HNC tissues. **b.** Real-time PCR analysis

showing CDKN1B expression in HNC tissues from chemotherapy sensitive patients (n = 20) and chemotherapy resistant patients (n = 20). **c.** Kaplan-Meier analyses of overall survival. Compared with patients with high CDKN1B expression, patients with low CDKN1B expression had a significantly lower overall survival rate. **d.** Kaplan-Meier analysis of overall survival of 499 HNC patients in the high and low CDKN1B groups using Gene Expression profiling Interactive Analysis (GEPIA, http://gepia.cancer-pku.cn/index.html).

Figure S24. Downregulation of ING5 is associated with malignant transformation and lower overall survival in HNC.

a. Downregulated ING5 levels were correlated with large tumor size, lymph node metastasis and advanced tumor stage in HNC tissues. **b.** Real-time PCR analysis showing ING5 expression in HNC tissues from chemosensitive patients (n = 20) and chemoresistant patients (n = 20). **c.** Kaplan-Meier analyses of overall survival. Compared with patients with high ING5 expression, patients with low ING5 expression had a significantly lower overall survival rate. **d.** Kaplan-Meier analysis of overall survival of 499 HNC patients in the high and low ING5 groups using Gene Expression profiling Interactive Analysis (GEPIA, http://gepia.cancer-pku.cn/index.html).
