# Supplementary material for: Exosomal miR-196a derived from cancer-associated fibroblasts confers cisplatin resistance in head and neck cancer through targeting CDKN1B and ING5
Source: Genome Biol. 2019 Jan 14;20:12. doi: 10.1186/s13059-018-1604-0 (PMC6332863; doi:10.1186/s13059-018-1604-0)
Supplement: Supplementary file 2 — Table S1. Dysregulated miRNAs in CAF-derived exosomes compared with NFs. (DOC 53 kb) [file 13059_2018_1604_MOESM2_ESM.doc]

**Table S1**. Dysregulated miRNAs in CAF-derived exosomes compared with NFs.

| **miRNA ID** | **Fold Change** | **Log2 Fold Change** | **Regulation** |
| --- | --- | --- | --- |
| hsa-miR-10b-5p | 20.6227 | 4.3661613 | up |
| hsa-miR-708-5p | 19.107079 | 4.2560353 | up |
| hsa-miR-335-5p | 16.674011 | 4.0595293 | up |
| hsa-miR-196a-5p | 13.829297 | 3.789656 | up |
| hsa-miR-10a-5p | 13.265075 | 3.7295609 | up |
| hsa-miR-31-3p | 11.158469 | 3.4800673 | up |
| hsa-miR-3177-3p | 7.6160192 | 2.929037 | up |
| hsa-miR-137-3p | 6.5102954 | 2.702723 | up |
| hsa-miR-218-5p | 6.065812 | 2.6007009 | up |
| hsa-miR-551b-3p | 5.9092126 | 2.5629659 | up |
| hsa-miR-191-3p | 5.4355454 | 2.4424248 | up |
| hsa-miR-539-5p | 5.284265 | 2.401703 | up |
| hsa-miR-3945 | 4.7015715 | 2.233143 | up |
| hsa-miR-885-3p | 4.6850257 | 2.228057 | up |
| hsa-let-7b-3p | 4.616716 | 2.206867 | up |
| hsa-miR-3622a-5p | 4.4101534 | 2.1408288 | up |
| hsa-miR-4708-5p | 4.361943 | 2.124971 | up |
| hsa-miR-4706 | 4.2535696 | 2.088674 | up |
| hsa-miR-377-3p | 4.1803994 | 2.0636408 | up |
| hsa-miR-16-2-3p | 4.0483646 | 2.0173392 | up |
| hsa-miR-526b-5p | 4.024814 | 2.008922 | up |
| hsa-miR-361-3p | 4.002327 | 2.000839 | up |
| hsa-miR-4646-3p | -4.35939 | -2.1241262 | down |
| hsa-miR-2276-3p | -4.3898444 | -2.1341698 | down |
| hsa-miR-135a-3p | -4.648009 | -2.2166128 | down |
| hsa-miR-4788 | -4.8816805 | -2.2873778 | down |
| hsa-miR-17-3p | -5.5055017 | -2.460874 | down |
| hsa-miR-205-5p | -6.680713 | -2.7400022 | down |
| hsa-miR-29b-2-5p | -7.9872403 | -2.997697 | down |
