# Supplementary material for: Exosomal miR-196a derived from cancer-associated fibroblasts confers cisplatin resistance in head and neck cancer through targeting CDKN1B and ING5
Source: Genome Biol. 2019 Jan 14;20:12. doi: 10.1186/s13059-018-1604-0 (PMC6332863; doi:10.1186/s13059-018-1604-0)
Supplement: Supplementary file 3 — Table S2. Gene ontology analysis revealed 12 candidate genes for miR-196a whose altered expression could contribute to the chemoresistant phenotype. (DOC 40 kb) [file 13059_2018_1604_MOESM3_ESM.doc]

**Table S2**. Gene ontology analysis revealed 12 candidate genes for miR-196a whose altered expression could contribute to the chemoresistant phenotype.

| **Target gene** | **Representative transcript** | **Putative binding sites** | **GO and KEGG analysis** |
| --- | --- | --- | --- |
| CDKN1B | NM_004064 | 1 | cell proliferation; cell apoptosis; cell cycle |
| CCND2 | NM_001759 | 1 | cell proliferation; cell cycle |
| UHRF2 | NM_152896 | 1 | cell cycle |
| ZNF655 | NM_138494 | 1 | cell cycle |
| NRAS | NM_002524 | 2 | cell proliferation; cell apoptosis |
| BIRC6 | NM_016252 | 1 | cell proliferation; cell apoptosis |
| PDGFRA | NM_006206 | 1 | cell proliferation |
| ING5 | NM_032329 | 1 | cell proliferation; cell apoptosis |
| SSR1 | NM_003144 | 1 | cell proliferation |
| OSMR | NM_003999 | 1 | cell proliferation |
| EPHA7 | NM_004440 | 1 | cell apoptosis |
| IKBKB | NM_001556 | 1 | cell apoptosis |
