# Supplementary material for: Exosomal miR-196a derived from cancer-associated fibroblasts confers cisplatin resistance in head and neck cancer through targeting CDKN1B and ING5
Source: Genome Biol. 2019 Jan 14;20:12. doi: 10.1186/s13059-018-1604-0 (PMC6332863; doi:10.1186/s13059-018-1604-0)
Supplement: Supplementary file 4 — Table S3. Relationship between tissue miR-196a level and clinicopathologic features in HNC; Table S4. Cox proportional hazards regression models for estimating overall survival related to Table S3. (DOC 86 kb) [file 13059_2018_1604_MOESM4_ESM.doc]

**Table S3. Relationship between miR-196a level and clinicopathologic features (N =** 80)

| **Characteristics** | **No. of Patients** | | ***miR-196a* △Ct^a^** | | ***Non-parametric*** | ***P value*** |
| --- | --- | --- | --- | --- | --- | --- |
|  | **No.** | **%** | **Mean ± SD** | | ***test value*** |  |
| **Age (years)** |  |  |  | |  |  |
| ≥ 60 | 39 | 48.8 | 9.44 ± 2.17 | | *Z = -1.112* | *0.266* |
| < 60 | 41 | 51.2 | 8.87 ± 1.83 | |  |  |
| **Gender** |  |  |  | |  |  |
| Male | 43 | 53.8 | 8.77 ± 1.68 | | *Z = -1.472* | *0.141* |
| Female | 37 | 46.2 | 9.59 ± 2.28 | |  |  |
| **Smoking history** |  |  |  | |  |  |
| Nonsmoker | 50 | 62.5 | 9.35 ± 2.11 | | *Z = -0.865* | *0.387* |
| Smoker | 30 | 37.5 | 8.82 ± 1.81 | |  |  |
| **Alcohol history** |  |  |  | |  |  |
| Nondrinker | 56 | 70.0 | | 9.21 ± 2.19 | *Z = -0.094* | *0.925* |
| Drinker | 24 | 30.0 | | 9.00 ± 1.53 |  |  |
| **Tumor size (cm)** |  |  | |  |  |  |
| ≤ 4 | 41 | 51.3 | | 10.23 ± 1.69 | *Z = -5.289* | *0.000* |
| > 4 | 39 | 48.7 | | 8.01 ± 1.67 |  |  |
| **Lymph node metastasis** |  |  | |  |  |  |
| pN1 to pN2 | 32 | 40.0 | | 8.29 ± 1.39 | *Z = -3.123* | *0.002* |
| pN0 | 48 | 60.0 | | 9.73 ± 2.16 |  |  |
| **TNM stage** |  |  | |  |  |  |
| Ⅰ-Ⅱ | 33 | 41.3 | | 10.49 ± 1.70 | *Z = -5.165* | *0.000* |
| Ⅲ-Ⅳ | 47 | 58.7 | | 8.21 ± 1.65 |  |  |
| **Pathological differentiation** |  |  | |  |  |  |
| Well | 50 | 62.5 | | 9.38 ± 2.19 | *Z = -1.173* | *0.241* |
| Moderately/poorly | 30 | 37.5 | | 8.77 ± 1.62 |  |  |
| **Disease Site** |  |  | |  |  |  |
| Tongue | 30 | 37.5 | | 9.34 ± 1.98 | *H = 5.475* | *0.242* |
| Gingival | 24 | 30.0 | | 9.55 ± 2.01 |  |  |
| Cheek | 13 | 16.2 | | 8.94 ± 2.29 |  |  |
| Floor of Mouth | 10 | 12.5 | | 8.24 ± 1.72 |  |  |
| Oropharynx | 3 | 3.8 | | 7.97 ± 1.59 |  |  |
| **Recurrence** |  |  | |  |  |  |
| Yes | 18 | 22.5 | | 8.80 ± 2.29 | *Z = -0.899* | *0.369* |
| No | 62 | 77.5 | | 9.25 ± 1.93 |  |  |
| **Local invasion** |  |  | |  |  |  |
| Yes | 38 | 47.5 | | 8.75 ± 1.94 | *Z = -1.742* | *0.082* |
| No | 42 | 52.5 | | 9.54 ± 2.02 |  |  |

Abbreviations: SD, standard deviation; pN, pathological lymph node status; TNM stage, tumor-lymph node-metastasis stage.

^a^△Ct indicates the difference in the cycle number at which a sample’s fluorescent signal passes a given threshold above baseline (Ct) derived from a specific gene compared with that of β-actin in tumor tissues.

**Table S4. Univariate and multivariate cox proportional hazards**

**regression models for estimating overall survival (N =** 80)

| **Characteristics** | **HR** | **95% CI** | ***P*** |
| --- | --- | --- | --- |
| **Univariate analysis** |  |  |  |
| Overall survival |  |  |  |
| Age (< 60 y vs ≥ 60 y) | 0.489 | 0.237 to 1.008 | 0.053 |
| Gender (male vs female) | 1.089 | 0.548 to 2.163 | 0.808 |
| Smoking history (smoker vs nonsmoker) | 1.483 | 0.746 to 2.947 | 0.261 |
| Alcohol history (drinker vs nondrinker) | 0.837 | 0.440 to 1.945 | 0.925 |
| Tumor size (≤ 4 cm vs > 4 cm) | 3.834 | 1.773 to 8.291 | 0.001 |
| Lymph node metastasis  (pN0 vs pN1 to pN2) | 9.390 | 4.006 to 22.011 | 0.000 |
| TNM stage (I-II vs III-IV) | 7.065 | 2.471 to 20.203 | 0.000 |
| Pathological differentiation  (Well vs Moderately to poorly) | 1.607 | 0.807 to 3.197 | 0.177 |
| Disease Site | 1.197 | 0.886 to 1.616 | 0.241 |
| Recurrence | 0.963 | 0.418 to 2.222 | 0.930 |
| Local invasion | 2.211 | 1.085 vs 4.502 | 0.029 |
| miR-196a expression (high vs low) | 3.187 | 1.507 to 6.743 | 0.002 |
| **Multivariate analysis** |  |  |  |
| Overall survival |  |  |  |
| Tumor size (≤ 4 cm vs > 4 cm) | 2.080 | 0.692 to 6.254 | 0.192 |
| Lymph node metastasis  (pN0 vs pN1 to pN2) | 7.875 | 2.397 to 25.865 | 0.001 |
| TNM stage (I-II vs III-IV) | 1.732 | 1.125 to 5.276 | 0.729 |
| Local invasion | 1.426 | 0.689 to 2.949 | 0.339 |
| miR-196a expression (high vs low) | 2.175 | 1.455 to 4.034 | 0.039 |

Abbreviations: CI, confidence interval; HR, hazard ratio; T, tumor stage;

pN, pathological lymph node status; TNM, tumor-lymph node-metastasis classification.
