# Supplementary material for: Exosomal miR-196a derived from cancer-associated fibroblasts confers cisplatin resistance in head and neck cancer through targeting CDKN1B and ING5
Source: Genome Biol. 2019 Jan 14;20:12. doi: 10.1186/s13059-018-1604-0 (PMC6332863; doi:10.1186/s13059-018-1604-0)
Supplement: Supplementary file 5 — Table S5. Relationship between plasma exosomal miR-196a level and clinicopathologic features in HNC; Table S6. Cox proportional hazards regression models for estimating overall survival related to Table S5. (DOC 86 kb) [file 13059_2018_1604_MOESM5_ESM.doc]

**Table S5. Relationship between plasma exosomal miR-196a level**

and clinicopathologic features (N = 74)

| **Characteristics** | **No. of Patients** | | ***miR-196a* △Ct^a^** | ***Non-parametric*** | ***P value*** |
| --- | --- | --- | --- | --- | --- |
|  | **No.** | **%** | **Mean ± SD** | ***test value*** |  |
| **Age (years)** |  |  |  |  |  |
| ≥ 60 | 40 | 54.1 | 16.54 ± 3.49 | *Z = -0.618* | *0.536* |
| < 60 | 34 | 45.9 | 16.29 ± 3.09 |  |  |
| **Gender** |  |  |  |  |  |
| Male | 55 | 74.3 | 16.42 ± 3.32 | *Z = -0.229* | *0.819* |
| Female | 19 | 25.6 | 16.43 ± 3.31 |  |  |
| **Smoking history** |  |  |  |  |  |
| Nonsmoker | 32 | 43.2 | 16.56 ± 3.28 | *Z = -0.426* | *0.670* |
| Smoker | 42 | 56.8 | 16.33 ± 3.34 |  |  |
| **Alcohol history** |  |  |  |  |  |
| Nondrinker | 48 | 64.9 | 16.71 ± 3.32 | *Z = -1.234* | *0.217* |
| Drinker | 26 | 35.1 | 15.90 ± 3.24 |  |  |
| **Tumor size (cm)** |  |  |  |  |  |
| ≤ 4 | 42 | 56.8 | 18.45 ± 2.606 | *Z = -5.990* | *0.000* |
| > 4 | 32 | 43.2 | 13.77 ± 2.66 |  |  |
| **Lymph node metastasis** |  |  |  |  |  |
| pN1 to pN2 | 33 | 44.6 | 14.33 ± 2.49 | *Z = -5.214* | *0.000* |
| pN0 | 41 | 55.4 | 18.12 ± 2.87 |  |  |
| **TNM stage** |  |  |  |  |  |
| Ⅰ-Ⅱ | 32 | 43.2 | 19.26 ± 1.36 | *Z = -6.667* | *0.000* |
| Ⅲ-Ⅳ | 42 | 56.8 | 14.27 ± 2.62 |  |  |
| **Pathological differentiation** |  |  |  |  |  |
| Well | 34 | 45.9 | 17.07 ± 3.03 | *Z = -1.464* | *0.143* |
| Moderately/poorly | 40 | 54.1 | 15.88 ± 3.44 |  |  |
| **Disease Site** |  |  |  |  |  |
| Tongue | 25 | 33.8 | 16.50 ± 3.67 | *H = 3.845* | *0.427* |
| Gingival | 10 | 13.5 | 16.65 ± 2.80 |  |  |
| Cheek | 14 | 18.9 | 17.69 ± 2.61 |  |  |
| Floor of Mouth | 12 | 16.2 | 16.00 ± 3.50 |  |  |
| Oropharynx | 13 | 17.6 | 15.14 ± 3.26 |  |  |
| **Recurrence** |  |  |  |  |  |
| Yes | 8 | 10.8 | 15.74 ± 3.35 | *Z = -0.557* | *0.577* |
| No | 66 | 89.2 | 16.51 ± 3.30 |  |  |
| **Local invasion** |  |  |  |  |  |
| Yes | 41 | 55.4 | 15.67 ± 3.19 | *Z = -2.202* | *0.028* |
| No | 33 | 44.6 | 17.37 ± 3.22 |  |  |

Abbreviations: SD, standard deviation; pN, pathological lymph node status; TNM stage, tumor-lymph node-metastasis stage.

^a^△Ct indicates the difference in the cycle number at which a sample’s fluorescent signal passes a given threshold above baseline (Ct) derived from a specific gene compared with that of β-actin in tumor tissues.

**Table S6. Univariate and multivariate cox proportional hazards**

regression models for estimating overall survival (N = 74)

| **Characteristics** | **HR** | **95% CI** | ***P*** |
| --- | --- | --- | --- |
| **Univariate analysis** |  |  |  |
| Overall survival |  |  |  |
| Age (< 60 y vs ≥ 60 y) | 0.835 | 0.386 to 1.806 | 0.646 |
| Gender (male vs female) | 1.261 | 0.506 to 3.145 | 0.618 |
| Smoking history (smoker vs nonsmoker) | 1.615 | 0.730 to 3.570 | 0.261 |
| Alcohol history (drinker vs nondrinker) | 1.244 | 0.553 to 2.797 | 0.597 |
| Tumor size (≤ 4 cm vs > 4 cm) | 7.185 | 2.690 to 19.189 | 0.000 |
| Lymph node metastasis  (pN0 vs pN1 to pN2) | 12.577 | 4.195 to 37.708 | 0.000 |
| TNM stage (I-II vs III-IV) | 5.069 | 1.021 to 14.883 | 0.001 |
| Pathological differentiation  (Well vs Moderately to poorly) | 1.494 | 0.665 to 3.359 | 0.331 |
| Disease Site | 1.201 | 0.927 to 1.555 | 0.165 |
| Recurrence | 1.824 | 0.626 to 5.320 | 0.271 |
| Local invasion | 1.976 | 0.856 vs 4.561 | 0.110 |
| Plasma exosomal miR-196a | 3.333 | 1.393 to 7.975 | 0.007 |
| expression (high vs low) |
| **Multivariate analysis** |  |  |  |
| Overall survival |  |  |  |
| Tumor size (≤ 4 cm vs > 4 cm) | 2.383 | 0.790 to 7.183 | 0.123 |
| Lymph node metastasis  (pN0 vs pN1 to pN2) | 6.926 | 2.074 to 23.128 | 0.002 |
| TNM stage (I-II vs III-IV) | 5.570 | 1.373 to 16.556 | 0.027 |
| Plasma exosomal miR-196a | 2.248 | 1.091 to 5.675 | 0.006 |
| expression (high vs low) |

Abbreviations: CI, confidence interval; HR, hazard ratio; T, tumor stage;

pN, pathological lymph node status; TNM, tumor-lymph node-metastasis classification.
