# Supplementary material for: Exosomal miR-196a derived from cancer-associated fibroblasts confers cisplatin resistance in head and neck cancer through targeting CDKN1B and ING5
Source: Genome Biol. 2019 Jan 14;20:12. doi: 10.1186/s13059-018-1604-0 (PMC6332863; doi:10.1186/s13059-018-1604-0)
Supplement: Supplementary file 6 — Table S7. Primary antibodies used in this study. (DOC 58 kb) [file 13059_2018_1604_MOESM6_ESM.doc]

**Table S7. Primary antibodies used in this study.**

| **Antigens** | **Manufacturer** | **Application** |
| --- | --- | --- |
| α-SMA [1A4] | Abcam | 1:200 for IF; 1:300 for WB; 1:50 for IHC |
| FAP | Abcam | 1:100 for IF; 1:1,000 for WB |
| FSP1 [EPR2761(2)] | Abcam | 1:100 for IF; 1:1,000 for WB |
| MRP2 | Proteintech | 1:1,000 for WB |
| ATP7B | Proteintech | 1:1,000 for WB |
| CTR1 | Cell Signaling Technology | 1:1,000 for WB |
| XIAP | Proteintech | 1:1,000 for WB |
| ERCC1 | Proteintech | 1:1,000 for WB |
| ERCC4 | SAB | 1:2,000 for WB |
| GSTK1 | Proteintech | 1:1,000 for WB |
| Alix | Invitrogen | 1:500 for WB |
| HSP90 | Boster | 1:400 for WB |
| HSP70 | Boster | 1: 400 for WB |
| CD63 [EPR5702] | Abcam | 1:1,000 for WB |
| CD9 | Proteintech | 1:1,000 for WB |
| Rab5 | Abcam | 1:500 for WB |
| GRP94 [1H10B7] | Proteintech | 1:1,000 for WB |
| p38 MAPK (D13E1) | Cell Signaling Technology | 1:1,000 for WB |
| Phospho-p38 MAPK (Thr180/Tyr182) (D3F9) | Cell Signaling Technology | 1:1,000 for WB |
| p44/42 MAPK (Erk1/2) (137F5) | Cell Signaling Technology | 1:1,000 for WB |
| Phospho-p44/42 MAPK (Erk1/2) (Thr202/Tyr204) (D13.14.4E) | Cell Signaling Technology | 1:1,000 for WB |
| JNK (D-2) | SANTA CRUZ | 1:500 for WB |
| Phospho-JNK (Thr183/Tyr185) (G-7) | SANTA CRUZ | 1:500 for WB |
| c-Myc (D3N8F) | Cell Signaling Technology | 1:1,000 for WB; 1:100 for ChIP |
| p53 (DO-7) | Cell Signaling Technology | 1:1,000 for WB; 1:100 for ChIP |
| NF-1 (D7R7D) | Cell Signaling Technology | 1:1,000 for WB; 1:50 for ChIP |
| ZRANB2 | Proteintech | 1:1,000 for WB |
| hnRNPA1 [D21H11] | Cell Signaling Technology | 1:1,000 for WB; 1:100 for RIP |
| ELAVL1 | Proteintech | 1:1,000 for WB |
| p27kip1 | Proteintech | 1:1,000 for WB; 1:200 for IHC |
| ING5 | Proteintech | 1:1,000 for WB; 1:200 for IHC |
| CDK2 | Proteintech | 1:1,000 for WB |
| CDK4 | Proteintech | 1:1,000 for WB |
| Cyclin D1 | Proteintech | 1:1,000 for WB |
| Cyclin E1 | Proteintech | 1:1,000 for WB |
| Bcl-2 | Proteintech | 1:1,000 for WB |
| Bax | Proteintech | 1:1,000 for WB |
| Caspase 3 | Proteintech | 1:1,000 for WB |
| PARP [46D11] | Cell Signaling Technology | 1:1,000 for WB |
| Ki-67 [20Raj1] | eBioscience | 1:200 for IHC |
| β-actin [2D4H5] | Proteintech | 1:5,000 for WB |
| H3 histone | Beyotime | 1:2,000 for WB |

Abbreviation: WB, western blot; IF, immunofluorescence; IHC, inmmunohistochemistry; RIP, RNA immunoprecipitation.
