# Supplementary material for: Exosomal miR-196a derived from cancer-associated fibroblasts confers cisplatin resistance in head and neck cancer through targeting CDKN1B and ING5
Source: Genome Biol. 2019 Jan 14;20:12. doi: 10.1186/s13059-018-1604-0 (PMC6332863; doi:10.1186/s13059-018-1604-0)
Supplement: Supplementary file 7 — Tables S8–S10. Sequences of primers and siRNAs used in this study. (DOC 152 kb) [file 13059_2018_1604_MOESM7_ESM.doc]

**Table S8.** Sequences of primers used for plasmid construction in this study.

| **Gene (Human)** | **Primer sequence (5’ to 3’)** |
| --- | --- |
| hnRNPA1 | F: GACTCAGATCTCGAGCTCAAGCTTCGAATTCGCCAC  CATGTCTAAGTCAGAGTCTCCT |
|  | R: CCTTGTCATCGTCATCCTTGTAGTCGGATCCAAATCT  TCTGCCACTGCCATAGC |
| CDKN1B  (without 3’ UTR) | F: CCGGAATTCGCCACCATGTCAAACGTGCGAGTGTCT |
|  | R: CCGCTCGAGTTACGTTTGACGTCTTCTGAGGC |
| CDKN1B 3' UTR  (with 3’ UTR) | F: GCCCCGCGGGTTTATCAGATACATCACTGCTTGAT |
|  | R: CCGCTCGAGTTACGTTTGACGTCTTCTGAGGC |
| ING5  (without 3’ UTR) | F: CCGGAATTCGCCACCATGGGAGCAAGAGTCACTCCACG |
|  | R: CCGCTCGAGTCAGGATGACCCGTCTGCC |
| ING5 3' UTR  (with 3’ UTR) | F: GGCTCTAGAGAGGAGCTGTGTGCCCGGATC |
|  | R: CCGCTCGAGTCAGGATGACCCGTCTGCC |
| **Gene 3’UTRs (Human)** | **Primer sequence (5’ to 3’)** |
| CDKN1B-3’UTR-WT | F: GCCCCGCGGGTTTATCAGATACATCACTGCTTGAT |
|  | R: GCCCTGCAGAGCCATATTATTTAACAAAAGAGGG |
| CDKN1B-3’UTR-MUT | F: GCCCCGCGGGTTTATCAGATACATCACTGCAAGAT |
|  | R: GCCCTGCAGAGCCATATTATTTAACAAAAGAGGG |
| ING5-3’UTR-WT | F: GGCTCTAGAGAGGAGCTGTGTGCCCGGATC |
|  | R: TCCGAAGATCTCGAGTGTGAGGAGGGCAGCCG |
| ING5-3’UTR-MUT | F: TTAAACAGCAAGTGTTCGGTTGATACTTAGTAACTCCG |
|  | R: GAACACTTGCTGTTTAAAAGGAAGGGAAATATTGCG |

**Table S9.** The small interfering RNA (siRNA) sequences used in this study.

| **Name** | **siRNA sequence** |
| --- | --- |
| siRNA-NC | Sense 5’-UAAGGCUAUGAAGAGAUACdTdT-3’ |
|  | Antisense 5’-GUAUCUCUUCAUAGCCUUAdTdT-3’ |
| siRNA-ERCC1 | Sense 5’-CAGGCGGCCCCUCAGACCUACdTdT-3’ |
|  | Antisense 5’-GUAGGUCUGAGGGGCCGCCUGdTdT-3’ |
| siRNA-ERCC4 | Sense 5’-CCCAUCGCUUGAAGUGGAAGAUUdTdT-3’ |
|  | Antisense 5’-AAUCUUCCACUUCAAGCGAUGGGdTdT-3’ |
| siRNA-c-Myc-1 | Sense 5’-GGAUGCUAUUGCUGUUCUAAUdTdT-3’ |
|  | Antisense 5’-UAGAACAGCAAUAGCAUCCUUdTdT-3’ |
| siRNA-c-Myc-2 | Sense 5’-GAAUUUCAAUCCUAGUAUAUAdTdT-3’ |
|  | Antisense 5’-UAUACUAGGAUUGAAAUUCUGdTdT-3’ |
| siRNA-c-Myc-3 | Sense 5’-GGAACUAUGACCUCGACUACGdTdT-3’ |
|  | Antisense 5’-UAGUCGAGGUCAUAGUUCCUGdTdT-3’ |
| siRNA-ZRANB2-1 | Sense 5’-GGAAGUUGAAGAUAAAGAAUCdTdT-3’ |
|  | Antisense 5’-UUCUUUAUCUUCAACUUCCUUdTdT-3’ |
| siRNA-ZRANB2-2 | Sense 5’-CCCUCAAGUUCAAGGUCUAGGdTdT-3’ |
|  | Antisense 5’-UAGACCUUGAACUUGAGGGGGdTdT-3’ |
| siRNA-hnRNPA1-1 | Sense 5’-CAGCUGAGGAAGCUCUUCAdTdT-3’ |
|  | Antisense 5’-UGAAGAGCUUCCUCAGCUGdTdT-3’ |
| siRNA-hnRNPA1-2 | Sense 5’-GCCGAAGAAGCAUCGUUAAAGdTdT-3’ |
|  | Antisense 5’-UUAACGAUGCUUCUUCGGCGGdTdT-3’ |
| siRNA-ELAVL1-1 | Sense 5’-AAGAGGCAAUUACCAGUUUCAdTdT-3’ |
|  | Antisense 5’-UGAAACUGGUAAUUGCCUCUUdTdT-3’ |
| siRNA-ELAVL1-2 | Sense 5’-UCAAAGACGCCAACUUGUAdTdT-3’ |
|  | Antisense 5’-UACAAGUUGGCGUCUUUGAdTdT-3’ |
| siRNA-CDKN1B | Sense 5’-GCAACCGACGAUUCUUCUAdTdT-3’ |
|  | Antisense 5’-UAGAAGAAUCGUCGGUUGCdTdT-3’ |
| siRNA-ING5 | Sense 5’-CCUACGAGAUGGUGGAUAAdTdT-3’ |
|  | Antisense 5’-UUAUCCACCAUCUCGUAGGdTdT-3’ |

**Table S10. The primers used for real-time PCR analysis.**

| **Gene** | **Primer sequences** |
| --- | --- |
| hsa-miR-10b-5p | forward 5’-TACCCTGTAGAACCGAATTTGTG-3’ |
| hsa-miR-708-5p | forward 5’-AAGGAGCTTACAATCTAGCTGGG-3’ |
| hsa-miR-335-5p | forward 5’-TCAAGAGCAATAACGAAAAATGT-3’ |
| hsa-miR-196a-5p | forward 5’-GGTAGGTAGTTTCATGTTGTTGGG-3’ |
| hsa-miR-10a-5p | forward 5’-TACCCTGTAGATCCGAATTTGTG-3’ |
| hsa-miR-31-3p | forward 5’-TGCTATGCCAACATATTGCCAT-3’ |
| hsa-miR-3177-3p | forward 5’-TGCACGGCACTGGGGACACGT-3’ |
| hsa-miR-137-3p | forward 5’-TTATTGCTTAAGAATACGCGTAG-3’ |
| hsa-miR-218-5p | forward 5’-TTGTGCTTGATCTAACCATGT-3’ |
| hsa-miR-551b-3p | forward 5’-GCGACCCATACTTGGTTTCAG-3’ |
| hsa-miR-191-3p | forward 5’-GCTGCGCTTGGATTTCGTCCCC-3’ |
| hsa-miR-539-5p | forward 5’-GGAGAAATTATCCTTGGTGTGT-3’ |
| hsa-miR-3945 | forward 5’-AGGGCATAGGAGAGGGTTGATAT-3’ |
| hsa-miR-885-3p | forward 5’-AGGCAGCGGGGTGTAGTGGATA-3’ |
| hsa-let-7b-3p | forward 5’-CTATACAACCTACTGCCTTCCC-3’ |
| hsa-miR-3622a-5p | forward 5’-CAGGCACGGGAGCTCAGGTGAG-3’ |
| hsa-miR-4708-5p | forward 5’-AGAGATGCCGCCTTGCTCCTT-3’ |
| hsa-miR-4706 | forward 5’-AGCGGGGAGGAAGTGGGCGCTGCTT-3’ |
| hsa-miR-377-3p | forward 5’-ATCACACAAAGGCAACTTTTGT-3’ |
| hsa-miR-16-2-3p | forward 5’-CCAATATTACTGTGCTGCTTTA-3’ |
| hsa-miR-526b-5p | forward 5’-CTCTTGAGGGAAGCACTTTCTGT-3’ |
| hsa-miR-361-3p | forward 5’-TCCCCCAGGTGTGATTCTGATTT-3’ |
| hsa-miR-4646-3p | forward 5’-ATTGTCCCTCTCCCTTCCCAG-3’ |
| hsa-miR-135a-3p | forward 5’-TATAGGGATTGGAGCCGTGGCG-3’ |
| hsa-miR-4788 | forward 5’-TTACGGACCAGCTAAGGGAGGC-3’ |
| hsa-miR-17-3p | forward 5’-ACTGCAGTGAAGGCACTTGTAG-3’ |
| hsa-miR-205-5p | forward 5’-TCCTTCATTCCACCGGAGTCTG-3’ |
| hsa-miR-29b-2-5p | forward 5’-CTGGTTTCACATGGTGGCTTAG-3’ |
| cel-miR-39 | forward 5’-GGTCACCGGGTGTAAATCAGCTTG-3’ |
| Human U6 snRNA | forward 5’-CTCGCTTCGGCAGCACA-3’ |
| Human MRP2 | forward 5’-CCCTGCTGTTCGATATACCAATC-3' |
|  | reverse 5’-TCGAGAGAATCCAGAATAGGGAC-3’ |
| Human ATP7B | forward 5’-ATATTGAGCGGTTACAAAGCACT-3' |
|  | reverse 5’-TGCCCCAAGGTCTCAGAATTA-3’ |
| Human CTR1 | forward 5’-GGGGATGAGCTATATGGACTCC-3' |
|  | reverse 5’-TCACCAAACCGGAAAACAGTAG-3’ |
| Human XIAP | forward 5’-AATAGTGCCACGCAGTCTACA-3' |
|  | reverse 5’-CAGATGGCCTGTCTAAGGCAA-3’ |
| Human ERCC1 | forward 5’-CTACGCCGAATATGCCATCTC-3' |
|  | reverse 5’-GTACGGGATTGCCCCTCTG-3’ |
| Human ERCC4 | forward 5’-CCTCTTTCGCCAGAAAAACAAAC-3' |
|  | reverse 5’-TTTACTGCTACATGGAACCTTGG-3’ |
| Human GSTK1 | forward 5’-TCTGGAAAAGATCGCAACGC-3' |
|  | reverse 5’-GCCCAAAGGCTCCGTATCTG-3’ |
| Human Bcl-2 | forward 5’-GGTGGGGTCATGTGTGTGG-3' |
|  | reverse 5’-CGGTTCAGGTACTCAGTCATCC-3’ |
| Human HRS | forward 5’-AGTGGCTGTCGGGTATTCATC-3' |
|  | reverse 5’-CCGTCCATATCCCTTGAAGAATC-3’ |
| Human TSG101 | forward 5’-GAGAGCCAGCTCAAGAAAATGG-3' |
|  | reverse 5’-TGAGGTTCATTAGTTCCCTGGA-3’ |
| Human STAM1 | forward 5’-AATCCCTTCGATCAGGATGTTGA-3' |
|  | reverse 5’-CGAGACTGACCAACTTTATCACA-3’ |
| Human VPS4B | forward 5’-ATGTCATCCACTTCGCCCAAC-3' |
|  | reverse 5’-TTGCTTGGCTTTATCACCCTG-3’ |
| Human CD9 | forward 5’-TCCACTATGCGTTGAACTGCT-3' |
|  | reverse 5’-GGTTTCGAGTACGTCCTTCTTG-3’ |
| Human CD63 | forward 5’-ATGCAGGCAGATTTTAAGTGCT-3' |
|  | reverse 5’-GTTCTTCGACATGGAAGGGATTT-3’ |
| Human nSMase2 | forward 5’-GCTGCCCTTTGCGTTTCTC-3' |
|  | reverse 5’-TCCAGCCGTGAATAGATGTAGG-3’ |
| Human PLD2 | forward 5’-CAGATGGAGTCCGATGAGGTG-3' |
|  | reverse 5’-CCGCTGGTATATCTTTCGGTG-3’ |
| Human RAB11A | forward 5’-CAACAAGAAGCATCCAGGTTGA-3' |
|  | reverse 5’-GCACCTACAGCTCCACGATAAT-3’ |
| Human RAB35 | forward 5’-TACTGTTGCGTTTTGCAGACA-3' |
|  | reverse 5’-CCCCGATAATACGTGGAGGTG-3’ |
| Human RAB2B | forward 5’-GTACGACATTACAAGGCGTGA-3' |
|  | reverse 5’-ATGTTGGAACTAGAGTGCTGC-3’ |
| Human RAB5A | forward 5’-CAAGGCCGACCTAGCAAATAA-3' |
|  | reverse 5’-GATGTTTTAGCGGATGTCTCCAT-3’ |
| Human RAB9A | forward 5’-AGGGACAACGGCGACTATC-3' |
|  | reverse 5’-TCTGACCTATCCTCGGTAGCA-3’ |
| Human RAB27A | forward 5’-GCTTTGGGAGACTCTGGTGTA-3' |
|  | reverse 5’-TCAATGCCCACTGTTGTGATAAA-3’ |
| Human RAB27B | forward 5’-TAGACTTTCGGGAAAAACGTGTG-3' |
|  | reverse 5’-AGAAGCTCTGTTGACTGGTGA-3’ |
| Human RAB7 | forward 5’-GTGTTGCTGAAGGTTATCATCCT-3' |
|  | reverse 5’-GCTCCTATTGTGGCTTTGTACTG-3’ |
| Human YKT6 | forward 5’-TGGTCACCTCAGTAGATACCAG-3' |
|  | reverse 5’-CTCGCTCTAACAGAGACTCCA-3’ |
| Human PKM2 | forward 5’-ATAACGCCTACATGGAAAAGTGT-3' |
|  | reverse 5’-TAAGCCCATCATCCACGTAGA-3’ |
| Human ATG7 | forward 5’-ATGATCCCTGTAACTTAGCCCA-3' |
|  | reverse 5’-CACGGAAGCAAACAACTTCAAC-3’ |
| Human CDKN1B | forward 5’-ATCACAAACCCCTAGAGGGCA-3' |
|  | reverse 5’-GGGTCTGTAGTAGAACTCGGG-3’ |
| Human CCND2 | forward 5’-CTGTCTCTGATCCGCAAGCAT-3' |
|  | reverse 5’-GGTGGGTACATGGCAAACTTAAA-3’ |
| Human UHRF2 | forward 5’-ATTGAGGACGTGTCTCGCAAA-3' |
|  | reverse 5’-GGTCTGGGCGAACTAGCAG-3’ |
| Human ZNF655 | forward 5’-GGCTGGTATTTCCTCCATCA-3' |
|  | reverse 5’-GCGAGTGTGTAGGTGACAGG-3’ |
| Human NRAS | forward 5’-TGAGAGACCAATACATGAGGACA-3' |
|  | reverse 5’-CCCTGTAGAGGTTAATATCCGCA-3’ |
| Human BIRC6 | forward 5’-TAGTGTATGCCTCGTTTGTTGG-3' |
|  | reverse 5’-TTCTGTGTGTGCTCACCTTTC-3’ |
| Human PDGFRA | forward 5’-TTTTTGTGACGGTCTTGGAAGT-3' |
|  | reverse 5’-TGTCTGAGTGTGGTTGTAATAGC-3’ |
| Human ING5 | forward 5’-ACCGCCATGTACTTGGAGC-3' |
|  | reverse 5’-CTGCTTTCTTATCTTCCGTCCTC-3’ |
| Human SSR1 | forward 5’-CTGCTTCTCTTACTCGTGTTCC-3' |
|  | reverse 5’-TCTTCTTCTACCTCGGCTTCAT-3’ |
| Human OSMR | forward 5’-AATGTCAGTGAAGGCATGAAAGG-3' |
|  | reverse 5’-GAAGGTTGTTTAGACCACCCC-3’ |
| Human EPHA7 | forward 5’-TCCGCTGCAGTACTCTCTCC-3' |
|  | reverse 5’-AGAGGCTCTTTGCTGCTGTC-3’ |
| Human IKBKB | forward 5’-CACTGCTTGATGGCAATCTG-3' |
|  | reverse 5’-AAATGAAAGAGCGCCTTGG-3’ |
| Human β-actin | forward 5’-TCACCCACACTGTGCCCATCTACGA-3' |
|  | reverse 5’-CAGCGGAACCGCTCATTGCCAATGG-3’ |
| Human miR-196a P1 | forward 5’-CTGTAATCCCAGCACTTT-3' |
|  | reverse 5’-GCTCACTGCAACCTCC-3’ |
| Human miR-196a P2 | forward 5’-TTCTTTTCAGGACAGGAGGGGATG-3' |
|  | reverse 5’-GCAGGGAAGTCCGAGGTAGCG-3’ |
| Human miR-196a P3 | forward 5’-CCGAAGGTCAGGAAGGAG-3' |
|  | reverse 5’-AGAATGGGATTTGGAGGG-3’ |
| Human miR-196a P4 | forward 5’-CTGAGTATAGATGCACCTTCG-3' |
|  | reverse 5’-AGACATTTGCTGCCTTCC-3’ |
| Human GAPDH | forward 5’-TACTAGCGGTTTTACGGGCG-3' |
|  | reverse 5’-TCGAACAGGAGGAGCAGAGAGCGA-3’ |
