# Supplementary material for: Exosomal miR-196a derived from cancer-associated fibroblasts confers cisplatin resistance in head and neck cancer through targeting CDKN1B and ING5
Source: Genome Biol. 2019 Jan 14;20:12. doi: 10.1186/s13059-018-1604-0 (PMC6332863; doi:10.1186/s13059-018-1604-0)
Supplement: Supplementary file 8 — Supplementary methods. (DOC 54 kb) [file 13059_2018_1604_MOESM8_ESM.doc]

**Supplementary Methods**

**Immunohistochemical analysis**

The detailed protocol was carried out as previously described [1]. After the incubation with primary antibodies, the target protein expressions were visualized using the DAKO ChemMate Envision Kit/HRP (Dako-Cytomation, USA). Five targeted areas of each section were randomly selected under the same conditions for further analysis. The protein expressions were quantitatively determined using Image-Pro Plus 6.0 software and calculated with the following formula: MOD = IOD SUM / area SUM (IOD: integrated optical density; MOD: mean optical density; IOD SUM: the accumulative IOD of targeted areas in one photo; area SUM: the sum of targeted areas). See **Additional file 6: Table S7** for antibodies used.

**miRNA array analysis**

The miRNA expression profiling was performed in two exosome samples (NF-derived exosomes and CAF-derived exosomes) using the Affymetrix miRNA array platform (oebiotech, Shanghai, China). Briefly, exosomal RNA was extracted using mirVana^TM^ miRNA Isolation Kit (Ambion, USA) and quantified by the NanoDrop ND-2100 (Thermo Scientific, USA). The resulting total RNA samples were tailed with Poly A, labeled with a FlashTag Biotin HSR RNA Labeling Kit (Affymetrix P/N 901910, Thermo Scientific, USA) and hybridized on an Affymetrix miRNA 3.0 array (Affymetrix). After washing, the microarrays were scanned by the Affymetrix Scanner 3000 (Affymetrix, Thermo Scientific, USA) and analyzed using Affymetrix GeneChip Command Console software (version 4.0, Affymetrix). The intensity of the signals was calculated after background subtraction, and replicated spots on the same slide were averaged to obtain the median intensity.

**Lentivirus package**

The 293T cells were transfected with 3 μg pLenti-miR-196a, pLenti-anti-miR-196a, pLenti-hnRNPA1 or pLenti-sh-hnRNPA1, 1 μg pCMV-VSV-G, and 3 μg pCMV-Delta8.9 using Lipofectamine 3000 reagent (Invitrogen, USA). After 20 h, the medium was replaced with 12 mL medium supplemented with 5% FBS. About 48 h later, the virus-containing supernatants were collected, filtered using a 0.45 μm cellulose acetate filter (Merck Millipore, USA) and stored at -80°C.

For the construction of miR-196-expressing, anti-miR-196a-expressing, hnRNPA1-expressing or hnRNPA1-silencing cell lines, the virus-containing supernatants were diluted 3 times with serum-free DMEM containing 10 μg/mL polybrene (YEASEN, China). The target cells at the density of 40% were incubated with the virus-containing mixture for 8 h. Thereafter, the medium was exchanged with DMEM containing 10% FBS and the cells were further selected with 10 μg/mL puromycin (Sigma-Aldrich, USA) for 4 weeks before experiments.

**EdU labelling assay**

The EdU labelling assay was implemented with the Cell-Light™ EdU Apollo®488 In Vitro Imaging kit (Ribobio, China) according to the manufacturer’s instructions. Cells were pretreated as indicated and seeded in 96-well plate at 70% confluence. After incubated with 5’-Ethynyl-2’-deoxyuridine (EdU) labelling medium for 2 h, the cells were stained with Apollo reagent for 30 min. Thereafter, the cells were washed and stained with Hoechst to detect nuclei. Five non-overlapping images were acquired under a fluorescence microscope (Axio Scope A1, Carl Zeiss, Germany) for further analysis.

**Plate colony formation assay**

After being pretreated as indicated, about 800 tumor cells were cultured in 6-well plate. Twenty-four hours later, the culture medium was replaced by fresh DMEM medium containing 10% FBS and 3 μM cisplatin, and the cells were culture for two weeks. Colonies were fixed and stained with crystal violet (YEASEN, China). The colony formation ability was assessed referring to the size and density of the colonies.

**Cell cycle analysis**

The cells were pretreated or transfected in advance, cultured in serum-free DMEM for 24 h and incubated with DMEM containing 10% FBS for 24 h. The cells were then harvested, washed with ice-cold PBS and fixed in ice-cold 70% ethanol overnight. Thereafter, the fixed cells were stained with PI staining buffer (BD Biosciences, USA) for 20 minutes on ice in the dark. Finally, the cell cycle of these cells were analyzed using a flow cytometer supplemented with the FlowJo software (FACS Calibur, BD Biosciences, USA).

**Cell apoptosis analysis**

Tumor cells were incubated with or without 10 μM cisplatin for 24 h after pretreatment or transfection. Cells were collected by trypsinization and washed twice with ice-cold PBS. As described in previous study [2], these cells were then stained using the FITC Annexin V Apoptosis Detection Kit (BD Pharmingen^TM^, USA) and quantified by flow cytometry (BD Biosciences, USA).

**Chromatin immunoprecipitation (ChIP) analysis**

As described in our previous study [1, 3], DNA was sheared to an average fragment size of 500 to 1,000 bp by sonication after the experimental cells (5×10^6^) were fixed in 1 % formaldehyde for 30 min at room temperature. Thereafter, chromatin was immunoprecipitated with NF-1, p53 and c-Myc antibodies, separately. The efficacy of the kit reagents (P-2025-48, Epigentek Group, USA) was identified with a positive control antibody (RNA polymerase II/RPII), a negative control normal rabbit IgG and GAPDH primers. A real-time PCR analysis was used to quantify the purified chromatin, and Signals obtained from the ChIP assay were divided by signals obtained from an input sample. In this study, 1 % of starting chromatin was used as the input, and then a dilution factor of 100 or 6.644 cycles (log2 of 100) was subtracted from the Ct value of diluted input (See primers of miR-196a promoter in Additional file 7: Table S10).

**Fluorescence in situ hybridization (FISH) assay for miR-196a**

A FISH assay was performed to detect the distribution of miR-196a in xenograft tumors. Tumor tissues were removed from the mice, fixed immediately in 10% formalin for up to 24 h, paraffin-embedded using a standard overnight procedure, and cut into 4-μm-thick sections. As reported previously [4, 5], the sections were deparaffinized at 60°C for 30 min, dewaxed in xylenes for 15 min, rehydrated in an ethanol dilution series (100%, 100%, 100%, 96%, 96%, 70%, 70% and 70%; each solution was incubated for 5 min) and submerged into PBS for 5 min. The tissue sections were digested with 10 μg/mL of proteinase K for 40 min at 37°C in a horizontal humidifying chamber to facilitate probe penetration and exposure of miRNA species, followed by incubation with freshly prepared 3% hydrogen peroxide temperature for 10 min. Subsequently, the sections were probed with 20 nM denatured double-FAM-labeled miR-196a LNA^TM^ probe (QIAGEN, Germany) solution at 55°C for 60 min in the hybridizer, washed thrice for 10 min using 55°C pre-warmed 0.1× SSC buffer and submerged in PBST for 5 min. Thereafter, the samples were blocked for 15 min, probed with sheep anti-FAM-POD (Roche, Switzerland; 1:400) for 60 min and incubated with TSA-FITC reagent (PerkinElmer, USA; 1:50) for 15 min at room temperature. Detection of the protein markers α-SMA was performed by incubation with an α-SMA primary antibody (Abcam, USA; 1:50), followed by an Alexa Fluor 549-conjugated anti-mouse IgG F(abʹ)2 fragment (Invitrogen, USA; 1:200). The cells were co-stained DAPI (Invitrogen, USA; 1:300) to detect nuclei. The LNA U6 snRNA probe was used as a positive control. Fluorescence signals were observed, imaged and analyzed using a TCS SP2 laser-scanning confocal microscope (Leica Microsystems, Germany).

**TUNEL assay**

The apoptotic cells in xenograft tumors were identified by terminal deoxynucleotidyl transferase-mediated dUTP nick end-labeling (TUNEL) staining using the in Situ Cell Detection Kit-POD method (Roche Diagnostics, USA) according to the manufacturer’s introductions. The positive cells were identified, counted (five random fields per slides) and analyzed.

**References**

1. Qin X, Yan M, Wang X, Xu Q, Wang X, Zhu X, Shi J, Li Z, Zhang J, Chen W. Cancer-associated Fibroblast-derived IL-6 Promotes Head and Neck Cancer Progression via the Osteopontin-NF-kappa B Signaling Pathway. Theranostics*.* 2018; 8:921-40.

2. Wu X, Cao W, Wang X, Zhang J, Lv Z, Qin X, Wu Y, Chen W. TGM3, a candidate tumor suppressor gene, contributes to human head and neck cancer. Mol Cancer*.* 2013; 12:151.

3. Zhang J, Qin X, Sun Q, Guo H, Wu X, Xie F, Xu Q, Yan M, Liu J, Han Z, Chen W. Transcriptional control of PAX4-regulated miR-144/451 modulates metastasis by suppressing ADAMs expression. Oncogene*.* 2015; 34:3283-95.

4. Nielsen BS, Holmstrom K. Combined microRNA in situ hybridization and immunohistochemical detection of protein markers. Methods Mol Biol*.* 2013; 986:353-65.

5. Sempere LF, Preis M, Yezefski T, Ouyang H, Suriawinata AA, Silahtaroglu A, Conejo-Garcia JR, Kauppinen S, Wells W, Korc M. Fluorescence-based codetection with protein markers reveals distinct cellular compartments for altered MicroRNA expression in solid tumors. Clin Cancer Res*.* 2010; 16:4246-55.
